# Exaggerated false positives by popular differential expression methods when analyzing human population samples

**DOI:** 10.1186/s13059-022-02648-4

**Published:** 2022-03-15

**Authors:** Yumei Li, Xinzhou Ge, Fanglue Peng, Wei Li, Jingyi Jessica Li

**Affiliations:** 1 Division of Computational Biomedicine, Department of Biological Chemistry, School of Medicine, University of California, Irvine, Irvine, CA 92697 USA; 2 Department of Statistics, University of California, Los Angeles, CA 90095 USA; 3 Department of Molecular and Cellular Biology, Baylor College of Medicine, Houston, TX 77030 USA; 4 Interdepartmental Program in Bioinformatics, University of California, Los Angeles, CA 90095 USA; 5 Department of Human Genetics, University of California, Los Angeles, CA 90095 USA; 6 Department of Computational Medicine, University of California, Los Angeles, CA 90095 USA; 7 Department of Biostatistics, University of California, Los Angeles, CA 90095 USA

## Abstract

When identifying differentially expressed genes between two conditions using human population RNA-seq samples, we found a phenomenon by permutation analysis: two popular bioinformatics methods, DESeq2 and edgeR, have unexpectedly high false discovery rates. Expanding the analysis to limma-voom, NOISeq, dearseq, and Wilcoxon rank-sum test, we found that FDR control is often failed except for the Wilcoxon rank-sum test. Particularly, the actual FDRs of DESeq2 and edgeR sometimes exceed 20% when the target FDR is 5%. Based on these results, for population-level RNA-seq studies with large sample sizes, we recommend the Wilcoxon rank-sum test.

**Supplementary Information:**

The online version contains supplementary material available at 10.1186/s13059-022-02648-4.

## Background

RNA-seq is a transcriptome profiling approach using deep-sequencing technologies [1–3]. Since RNA-seq was developed over a decade ago, it has become an indispensable tool for genome-wide transcriptomic studies. One primary research task in these studies is the identification of differentially expressed genes (DEGs) between two conditions (e.g., tumor and normal samples) [3]. This task’s long-standing, core challenge is the small sample size, typically two or three replicates per condition. Many statistical methods have been developed to address this issue by making parametric distributional assumptions on RNA-seq data, and the two most popular methods of this type are DESeq2 [4] and edgeR [5]. However, as sample sizes have become large in population-level RNA-seq studies, where dozens to thousands of samples were collected from individuals [6, 7], a natural question to ask is whether DESeq2 and edgeR remain appropriate.

## Results and discussion

To evaluate the performance of DESeq2 and edgeR on identifying DEGs between two conditions, we applied the two methods to 13 population-level RNA-seq datasets with total sample sizes ranging from 100 to 1376 (Additional file 1: Table S1). We found that DESeq2 and edgeR had large discrepancies in the DEGs they identified on these datasets (Additional file 1: Fig. S1). In particular, 23.71–75% of the DEGs identified by DESeq2 were missed by edgeR. The most surprising result is from an immunotherapy dataset (including 51 pre-nivolumab and 58 on-nivolumab anti-PD-1 therapy patients) [8]: DESeq2 and edgeR had only an 8% overlap in the DEGs they identified (DESeq2 and edgeR identified 144 and 319 DEGs, respectively, with a union of 427 DEGs but only 36 DEGs in common). This phenomenon raised a critical question: did DESeq2 and edgeR reliably control their false discovery rates (FDRs) to the target 5% on this dataset?

From the literature, we found that several studies had reported the anticonservative behavior of DESeq2 and edgeR [9–11]; however, they were restricted to using simulated datasets with small sample sizes. Hence, for our large-sample-size scenario, their findings did not provide a direct answer to our question. Nevertheless, large sample sizes allowed us to generate permuted datasets to evaluate the FDRs without relying on model assumptions.

**Fig. 1:**
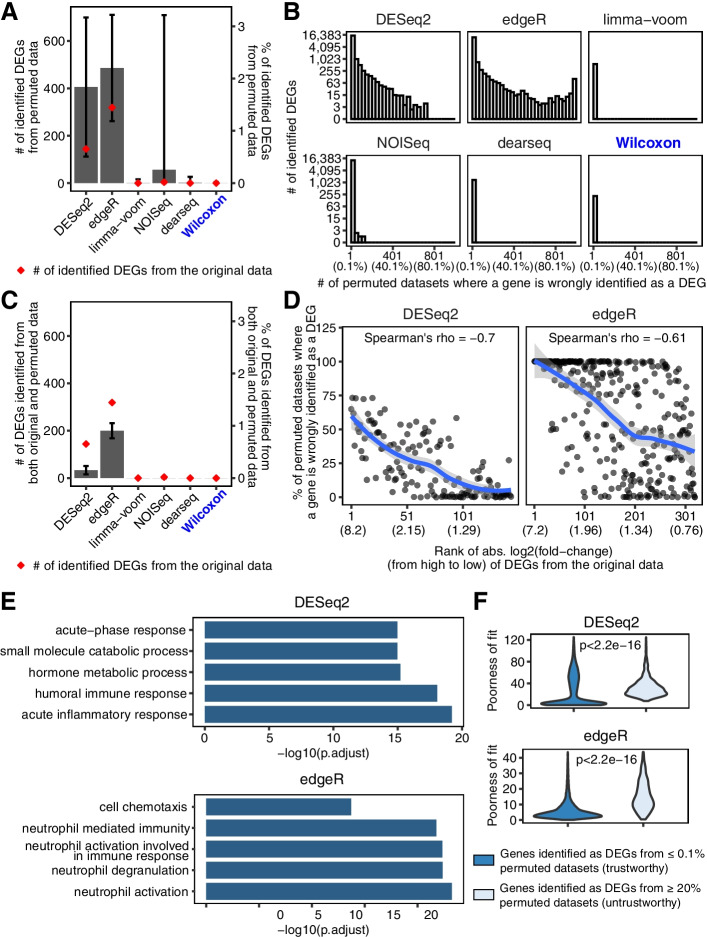
Exaggerated false DEGs identified by DESeq2 and edgeR from anti-PD-1 therapy RNA-seq datasets. **A** Barplot showing the average numbers of DEGs (left *y*-axis) and the proportion of DEGs out of all genes (right y-axis) identified from 1000 permuted datasets. The error bars represent the standard deviations of 1000 permutations. The red dots indicate the numbers of DEGs identified from the original dataset. **B** The distributions of the number of permuted datasets where a gene was mistakenly identified as a DEG. The percentages corresponding to the numbers are listed in parentheses below the numbers. **C** Barplot showing the average numbers of DEGs (left *y*-axis) and the proportion of DEGs out of all genes (right *y*-axis) identified from both the original dataset and any of the 1000 permuted datasets. The error bars represent the standard deviations of 1000 permutations. The red dots indicate the numbers of DEGs identified from the original dataset. **D** Percentage of permuted datasets where a DEG identified from the original dataset was also identified as a DEG. The genes are sorted by absolute log2(fold-change) in the original dataset in decreasing order. The absolute log2(fold-change) values corresponding to the ranks are listed in parentheses below the ranks. The line is fitted using the loess method, and the shaded areas represent 95% confidential intervals. **E** GO term enrichment for the DEGs identified from at least 10% permuted datasets. The top 5 enriched biological processes GO terms are shown. The analyses were performed using R package clusterProfiler. P.adjust represents the adjusted *p*-value using the Benjamini & Hochberg method. **F** Violin plots showing the poorness of fitting the negative binomial model to the genes identified by DESeq2 or edgeR as DEGs from ≥ 20% vs. ≤ 0.1% permuted datasets. The poorness of fit for each gene is defined as its negative log_10_(*p*-value) from the goodness-of-fit test for the negative binomial distributions estimated by DESeq2 or edgeR. The *p*-value in each panel was calculated by the Wilcoxon rank-sum test to compare the two groups of genes' poorness-of-fit values

To answer this question, we first generated 1000 negative-control datasets by randomly permuting the two-condition labels (pre-nivolumab and on-nivolumab) of the 109 RNA-seq samples in this immunotherapy dataset (Methods). Since any DEGs identified from these permuted datasets are considered as false positives, we used these permuted datasets to evaluate the FDRs of DESeq2 and edgeR. Surprisingly, DESeq2 and edgeR had 84.88% and 78.89% chances, respectively, to identity more DEGs from the permuted datasets than from the original dataset (Fig. 1A). In particular, DESeq2 and edgeR mistakenly identified 30 and 233 genes as DEGs, respectively, from 50% permuted datasets (Fig. 1B). Even more, among the 144 and 319 DEGs that DEseq2 and edgeR identified respectively from the original dataset, 22 (15.3%) and 194 (60.8%) DEGs were identified from at least 50% of permuted datasets, suggesting that these DEGs were spurious (Fig. 1C). These results raised the caution about exaggerated false positives found by DESeq2 and edgeR on the original dataset.

What is more counter-intuitive, the genes with larger fold changes estimated by DESeq2 and edgeR (between the two conditions in the original dataset) were more likely to be identified as DEGs by the two methods from the permuted datasets (Fig. 1D and Additional file 1: Fig. S2). This finding is consistent with a recent paper, which also reported that selecting the genes with the largest estimated differences between the two conditions would inflate the FDR [12]. As biologists tend to believe that these large-fold-change genes are more likely true DEGs (which is not necessarily true because a dataset may contain no true DEGs at all), the fact that these genes are false positives would likely waste experimental validation efforts.

Out of curiosity and as a means of verification, we investigated the biological functions of the spurious DEGs identified by DESeq2 or edgeR from the permuted datasets. Unexpectedly, these spurious DEGs' top 5 enriched gene ontology (GO) terms included immune-related terms (Fig. 1E). Hence, if these spurious DEGs were not removed by FDR control, they would mislead researchers to believe that there was an immune response difference between pre-nivolumab and on-nivolumab patients, an undoubtedly undesirable consequence that DEG analysis must avoid.

Then, a question followed: why did DESeq2 and edgeR make so many false-positive discoveries from this immunotherapy dataset? Our immediate hypothetical reason was the violation of the negative binomial model assumed by both DESeq2 and edgeR [13]. To check this hypothesis, we selected two groups of genes: (1) the genes identified as DEGs from ≥ 20% permuted datasets and (2) the genes identified as DEGs from ≤ 0.1% permuted datasets; then, we evaluated how well the negative binomial model fit the genes in each group. In line with our hypothesis, the model fitting was worse for the genes in the first group, consistent with the fact that these genes were spurious DEGs (Fig. 1F and Additional file 1: Fig. S3). Further checking of the spurious DEGs enriched in immune-related GO terms revealed that these genes also had worse model fitting than those genes in the second group (Additional file 1: Fig. S4). Considering that a likely cause of the model violation is the existence of outliers, we examined all the genes that were mistakenly identified as DEGs in at least 10% of permuted datasets, and we detected the existence of outliers in all these genes’ measurements relative to the assumed negative binomial model (Additional file 1: Fig. S5). It is well known that estimating the mean is not informative in the existence of outliers. However, in parametric methods like edgeR and DESeq2, the null hypothesis is that a gene has the same mean under the two conditions. Hence, it is expected that the testing result would be severely affected by the existence of outliers. In contrast, the Wilcoxon rank-sum test is more robust to outliers due to its different null hypothesis: a gene’s measurement under one condition has equal chances of being less or greater than its measurement under the other condition. That is, the Wilcoxon rank-sum test concerns more about the ranks than the magnitudes of measurements, making it robust to outliers.

Motivated by these findings, we further benchmarked DESeq2 and edgeR along with four other representative DEG identification methods on this immunotherapy dataset and the other 12 population-level RNA-seq datasets from two large consortia, the Genotype-Tissue Expression (GTEx) project [7] and the Cancer Genome Atlas (TCGA) [6] (Additional file 1: Table S1). In particular, on GTEx datasets, differential expression analysis can be performed between two tissues or cell types; on TCGA datasets, differential expression analysis can be performed between two disease statuses or biological conditions. The four representative methods include two popular methods limma-voom [14, 15] and NOISeq [16], a new method dearseq [11] (which claimed to overcome the FDR inflation issue of DESeq2 and edgeR on large-sample-size data), and the classic Wilcoxon rank-sum test [17]. Note that DESeq2, edgeR, and limma-voom are parametric methods that assume parametric models for data distribution, while NOISeq, dearseq, and the Wilcoxon rank-sum test are non-parametric methods that are less restrictive but require large sample sizes to have good power. (Also note that the GTEx project used DESeq2 and NOISeq for DEG identification.) Using permutation analysis on these datasets, we found that DESeq2 and edgeR consistently showed exaggerated false positives (reflected by their actual FDRs far exceeding the target FDR thresholds) compared to the other four methods (Additional file 1: Figs. S6-S17).

**Fig. 2:**
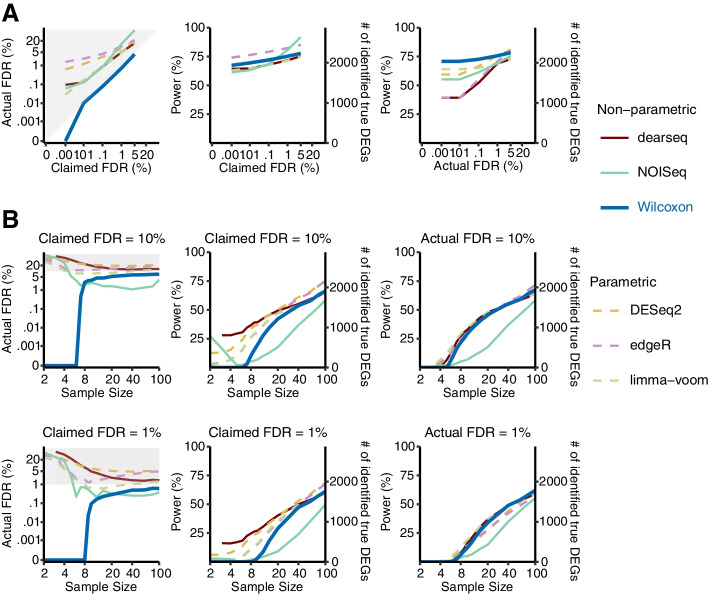
The Wilcoxon rank-sum test has the best FDR control and power on heart left ventricle vs. atrial appendage GTEx datasets with semi-synthetic ground truths. **A** The FDR control (left panel), power (middle panel) given the claimed FDRs, and power given the actual FDRs (right panel) under a range of FDR thresholds from 0.001 to 5%. **B** The FDR control (left), power given the claimed FDRs (middle), and power given the actual FDRs (right) for a range of per-condition sample sizes from 2 to 100, under FDR thresholds 10% (top panels) and 1% (bottom panels). The claimed FDRs, actual FDRs, and power were all calculated as the averages of 50 randomly down-sampled datasets

While the permutation analysis created true negatives (non-DEGs) to allow FDR evaluation, it did not allow the evaluation of DEG identification power, which would require true positives (DEGs) to be known. Hence, we generated 50 (identically and independently distributed) semi-synthetic datasets with known true DEGs and non-DEGs from each of the 12 GTEx and TCGA datasets. Then, we used these semi-synthetic datasets to evaluate the FDRs and power of the six DEG identification methods (Methods). In comparing 386 heart left ventricle samples and 372 atrial appendage samples in a GTEx dataset, only the Wilcoxon rank-sum test consistently controlled the FDR under a range of thresholds from 0.001 to 5% (Fig. 2A). In contrast, the other five methods, especially DESeq2 and edgeR, failed to control the FDR consistently. Moreover, we compared the power of the six methods conditional on their actual FDRs (Methods). (Due to the tradeoff between FDR and power, power comparison is only valid when the actual FDRs are equal.) As shown in Fig. 2A, the Wilcoxon rank-sum test outperformed the other five methods in terms of power.

Finally, to investigate how sample sizes would influence the performance of the six methods, we down-sampled each semi-synthetic dataset to obtain per-condition sample sizes ranging from 2 to 100. Again, only the Wilcoxon rank-sum test consistently controlled the FDR at all sample sizes (Fig. 2B, Additional file 1: Fig. S18). Granted, at the FDR threshold of 1%, the Wilcoxon rank-sum test had almost no power when the per-condition sample size was smaller than 8—an expected phenomenon for its nonparametric nature. However, when the per-condition sample size exceeded 8, the Wilcoxon rank-sum test achieved comparable or better power than the three parametric methods (DESeq2, edgeR, and limma-voom) and the new method dearseq, and it clearly outpowered NOIseq (Fig. 2B, Additional file 1: Fig. S18). Considering that the proportion of DEGs might affect the performance of DEG identification methods [10], we next generated semi-synthetic datasets with five proportions of DEGs (1%, 3%, 5%, 9%, and 20%) and evaluated the performance of DEG identification methods accordingly. The results show that the Wilcoxon rank-sum test consistently controlled the FDR and achieved the highest power (conditional on the actual FDRs) across all proportions of DEGs (Additional file 1: Fig. S19). In contrast, other methods consistently failed to control the FDR across all proportions. These observations were consistent across all 600 semi-synthetic datasets (Additional file 1: Figs. S20-S30).

The three parametric methods—DESeq2, edgeR, and limma—have long been dominant in transcriptomic studies. For example, the GTEx project, a consortium effort studying gene expression and regulation in normal human tissues, used DESeq2 coupled with NOISeq to find DEGs between tissues [18]; several studies applied edgeR or limma to TCGA RNA-seq data to find DEGs between tumor and normal samples [19–21]; moreover, researchers used DESeq2 to detect DEGs between responders and non-responders of the immunotherapy [8, 22]. However, while the three parametric methods were initially designed to address the small-sample-size issue, these population-level studies had much larger sample sizes (at least dozens) and thus no longer needed restrictive parametric assumptions. Moreover, violation of parametric assumptions would lead to ill-behaved *p*-values and likely failed FDR control [23], an issue independent of the sample size.

Although several studies had evaluated the performance of various DEG identification methods before our study [9, 10, 24–34], they had been restricted to using simulated datasets with small sample sizes (Additional file 2). Unlike all previous studies, our study evaluated the FDRs of DEG identification methods by permuting real RNA-seq datasets, without relying on any model assumptions. We could use real datasets for FDR evaluation because our datasets have large sample sizes, allowing us to generate a large number of permuted datasets to ensure accurate FDR estimation. In contrast, previous studies had focused on small sample sizes, a scenario where FDR cannot be reliably estimated by permutation because the number of possible permutations is too small; that is why they all had to use model-based simulation for FDR estimation, leaving the doubt if their simulated data resembled real data.

Another novelty of our study is the recommendation of the classical Wilcoxon rank-sum test, which had been ignored by all existing benchmark studies (Additional file 2). For the first time, we found that the Wilcoxon rank-sum test consistently controlled the FDR and achieved good power for DEG identification from large-sample-size RNA-seq data. Although the recently developed dearseq method [11] was claimed to overcome the inflated FDR issue of DESeq2 and edgeR, our evaluation shows that dearseq still had the issue and did not outperform the Wilcoxon rank-sum test.

In summary, our study shows the superiority of the Wilcoxon rank-sum test, a powerful and robust non-parametric test also known as the Mann-Whitney test developed in the 1940s [17, 35–38], for two-condition comparisons on large-sample-size RNA-seq datasets. The Wilcoxon rank-sum test is known to be powerful for skewed distributions, as is the case with gene expression counts measured by RNA-seq. Our results also echo the importance of verifying FDR control by permutation analysis. Beyond RNA-seq data analysis, our study suggests that, for population-level studies with large sample sizes, classic non-parametric statistical methods should be considered as the baseline for data analysis and new method benchmarking.

We did not include the two-sample t test or the Welch’s correction as an alternative to the Wilcoxon rank-sum test for three reasons. First, if a gene’s two sets of observations under the two conditions (i.e., two samples) are from non-Gaussian distributions (which is the case for RNA-seq data), the *t* test is only valid when the two sample sizes are large and the central limit theorem holds (then the gene's two sample means are approximately Gaussian distributed); however, the central limit theorem only holds when the two samples are from distributions with finite second moments, excluding the possibility that samples may come from heavy-tailed distributions with infinite second moments [39]. Second, the *t* test is not invariant to non-linear monotone transformations on the observations; for example, the two samples may have the same population mean on the original scale (i.e., the null hypothesis is true) but different population means on the log scale (i.e., the null hypothesis is false); there is no consensus on what transformation is optimal for RNA-seq data. In contrast, the Wilcoxon rank-sum test is invariant to monotone transformations. Third, the *t* test only concerns the mean difference between the two samples’ distributions, but the mean parameter is not informative if a distribution is heavily skewed, and estimating the mean parameter is not robust to the existence of outliers. Unlike the *t* test, the Wilcoxon rank-sum test has no requirement on the data distributions. It concerns the null hypothesis that a random observation from one distribution has equal chances of being less or greater than a random observation from the other distribution, an informative null hypothesis to test against regardless of the skewness of distributions. Admittedly, the DE genes found by the Wilcoxon rank-sum test may have distributional differences other than the mean difference between the two conditions [40], but we do not regard this as a notable drawback of the Wilcoxon rank-sum test. The reason is that genes may still be of biological interest if they have the same mean but different spread under the two conditions.

Finally, we note that, unlike DESeq2, edgeR, limma-voom, and dearseq, the Wilcoxon rank-sum test is a non-regression-based method, making it unable to adjust for confounders. Hence, to use the Wilcoxon rank-sum test for DEG identification, researchers can normalize RNA-seq samples to remove batch effects or use the probabilistic index models to adjust the Wilcoxon rank-sum test for covariates [41, 42].

## Conclusions

In conclusion, when the per-condition sample size is less than 8, parametric methods may be used because their power advantage may outweigh their possibly exaggerated false positives. However, if users are concerned about FDR control, our recent method Clipper provides a *p*-value-free FDR control solution for small-sample-size data [43]; for large-sample-size data, the Wilcoxon rank-sum test is our recommended choice for its solid FDR control and good power.

## Methods

### Selection of DEG identification methods

We selected the three parametric methods for identifying differentially expressed gene (DEGs) from RNA-seq data based on popularity: DESeq2 [4], edgeR [5], and limma-voom [15] (with 33,969, 24,037, and 15,786 citations, respectively, in Google Scholar as of 24 February 2022). We chose the non-parametric method NOISeq [16] because the Genotype-Tissue Expression (GTEx) consortium used it to identify DEGs between tissues, and we used GTEx RNA-seq datasets in our study. We also chose the newly developed non-parametric method dearseq [11], which claimed that it overcomes the FDR control issue of DESeq2, edgeR, and limma-voom on large-sample-size data. Moreover, we included the Wilcoxon rank-sum test, a classical non-parametric statistical test that compares two samples (i.e., a gene’s two sets of expression levels measured under two conditions).

### Datasets

Since we focused on large samples, we chose RNA-seq datasets from spotlight population-level studies (where samples are from healthy individuals or patients) including GTEx [7], TCGA [6], and an immunotherapy study [8]. We did not select datasets by any criteria other than the sample size (we required at least 50 samples per condition). In particular, GTEx and TCGA are two consortia that generated large-scale RNA-seq datasets. On GTEx datasets, differential expression analysis can be performed between two tissues or cell types. On TCGA datasets, differential expression analysis can be performed between two disease statuses or biological conditions. The immunotherapy dataset represents an important research topic where differential expression is performed to understand the effectiveness of immunotherapy treatment.

- For the immunotherapy study, we selected one dataset with a total sample size of 109, including 51 pre-nivolumab and 58 on-nivolumab anti-PD-1 therapy melanoma samples (https://www.ncbi.nlm.nih.gov/geo/query/acc.cgi?acc=GSE91061).
- For TCGA data, we selected RNA-seq datasets of six cancer types, which have paired normal tissues and sample sizes greater than 50 for both cancer and normal tissues. Then, we downloaded the gene read count matrices of these selected datasets from GDC Xena Hub (https://xenabrowser.net/datapages/?hub=https://gdc.xenahubs.net:443, release v18.0).
- For GTEx data, we selected six pairs of tissues with sample sizes ranging from 126 to 706. Then we downloaded the gene read count matrices of these tissue samples from the GTEx Portal (https://storage.googleapis.com/gtex_analysis_v8/rna_seq_data/GTEx_Analysis_2017-06-05_v8_RNASeQCv1.1.9_gene_reads.gct.gz, GTEx Analysis V8).

Additional file 1: Table S1 lists the detailed information of the datasets used in this study.

### The identification of DEGs

All six methods (DESeq2, edgeR, limma-voom, NOISeq, dearseq, and the Wilcoxon rank-sum test) took a read count matrix and a condition label vector as input. The parameters were set based on the user guides of these methods’ software packages.

- For DESeq2 (v1.28.1), we used the *DESeq* function to perform differential analysis, followed by generating the results using the *results* function.
- For edgeR (v3.30.3), we first filtered out genes with very low counts using the *filterByExpr* function, followed by normalization using the trimmed mean of M values (TMM) method. Then, the quasi-likelihood F-test was used for differential analysis.
- For limma-voom (v3.44.3), we filtered genes and calculated the normalization factor in the same way as we did for edgeR. Then we applied the voom transformation to the normalized and filtered count matrix and performed the differential analysis using the *lmFit* and *eBayes* functions.
- For NOISeq (v2.31.0), we used the *noiseqbio* function to identify DEGs.
- For dearseq, the filtering and normalization steps were the same as those for edgeR. Then, we used the *dear_seq* function with the asymptotic test to identify DEGs.
- For the Wilcoxon rank-sum test, the filtering and normalization steps were the same as those for edgeR. For *p*-value calculation, we input each gene’s counts-per-million (CPM) values into the *wilcox.test* function in R (v4.0.2). Then, we set a *p*-value cutoff based on an FDR threshold using the Benjamini & Hochberg method.

Specifically, DEGs were selected based on the corresponding FDR threshold for all the six methods (FDR < 0.05 for the immunotherapy dataset; FDR < 0.01 for GTEx and TCGA datasets).

### The generation of permuted and semi-synthetic data from the original RNA-seq data

From each original RNA-seq dataset, we generated permuted datasets between two conditions (pre-therapy and on-therapy samples for the immunotherapy data; two tissue types for GTEx data; normal and tumor samples for TCGA data). We used ***M*** to denote a gene-by-sample read count matrix (with genes as rows and samples as columns) and ***C*** to denote the vector of sample conditions labels (corresponding to the columns of ***M***). Then, we generated a permuted dataset by randomly permuting all values in ***C*** and keeping the original order of samples in ***M***. We repeated this permutation procedure for 1000 times to generate 1000 permuted datasets.

The semi-synthetic datasets were generated based on original RNA-seq samples from GTEx and TCGA. We first used all six DEG identification methods to identify DEGs from each original dataset containing two conditions. We then defined *true DEGs* as the genes identified as DEGs by all six methods at a very small FDR threshold (0.0001%). We used ***X*** and ***Y*** to denote the read count matrices from the two conditions, and ***X***_***i***_ and ***Y***_***i***_ to denote the read counts of gene *i* from the two conditions (i.e., the *i*-th row of ***X*** and ***Y***). Then, we generated semi-synthetic datasets ***X***^′^ and ***Y***^′^ in the following way: for each semi-synthetic datasets, we first randomly sampled *k* selected true DEGs from all true DEGs and preserved the read counts of these selected true DEGs; for each of the remaining genes, we randomly permuted its read counts between the two conditions. That is, ${\boldsymbol{X}}_{\boldsymbol{i}}^{\prime }={\boldsymbol{X}}_{\boldsymbol{i}}$ and ${\boldsymbol{Y}}_{\boldsymbol{i}}^{\prime }={\boldsymbol{Y}}_{\boldsymbol{i}}$ if gene *i* is a selected true DEG, $\left({\boldsymbol{X}}_{\boldsymbol{i}}^{\prime },{\boldsymbol{Y}}_{\boldsymbol{i}}^{\prime}\right)={\sigma}_i\left({\boldsymbol{X}}_{\boldsymbol{i}},{\boldsymbol{Y}}_{\boldsymbol{i}}\right)$ if gene *i* is not a selected true DEG, where *σ*_*i*_ is a random permutation of values in ***X***_***i***_ and ***Y***_***i***_. We repeated this procedure independently for 50 times to generate 50 semi-synthetic datasets. To generate down-sampled semi-synthetic datasets with a per-condition sample size of *n*, we randomly sampled *n* columns from ***X***^′^ and ***Y***^′^ each. The number of selected true DEGs *k* is 50% of the number of all true DEGs in most results. For results in first 4 rows of Additional file 1: Fig. S19, we also varied the percentages of selected true DEGs: 10%, 30%, and 90% of all true DEGs (1%, 3%, 9% of all genes). For the results in the last row of Additional file 1: Fig. S19, we defined selected true DEGs as the genes identified as DEGs by all six methods at FDR threshold = 1%.

### Calculation of FDR, power, and poorness of model fit

The FDR is defined as the expectation of the false discovery proportion (FDP), the proportion of false positives among all the discoveries. The FDR cannot be directly observed, but the FDP can be calculated from benchmark datasets with known true positives and negatives. In our semi-synthetic data analysis, we defined true DEGs as true positives and the remaining genes as true negatives. First, we calculated the FDP of each DEG identification method (e.g., DESeq2) on each semi-synthetic dataset. Second, we calculated the method’s (approximate) FDR by taking the average of its FDPs on the 50 semi-synthetic datasets.

The power of a DEG identification method is defined as the probability of identifying a gene as a DEG conditional on that the gene is a true DEG. It can also be considered as the expectation of the empirical power, which is the proportion of true DEGs being identified as DEGs. In our semi-synthetic datasets with true DEGs and true non-DEGs, we calculated the power of a method by taking the average of its empirical power on the 50 semi-synthetic datasets, similar to how we calculated the FDR.

We used the goodness-of-fit test to evaluate how well a gene’s read counts under a condition can be fit by the negative binomial models estimated by DESeq2 and edgeR. To remove batch effects, we used the normalized read counts output by DESeq2 and edgeR. For each gene under each condition and each method (DESeq2 or edgeR), we conducted the goodness-of-fit test on the method’s normalized read counts, with the method’s estimated dispersion parameter of the negative binomial distribution. The goodness-of-fit test was implemented using the function *goodfit* in the R package *vcd* as 

summary(goodfit(round(normalized_counts), type = "nbinomial", par = list(size = 1/dispersion)))

which returns a *p*-value. A smaller *p*-value indicates a poorer fit. Hence, we defined the poorness of fit as the negative log_10 _(*p*-value).

## Supplementary Information

**Additional file 1.** Figs. S1 to S30 and Table S1.

**Additional file 2.** Summary of studies comparing DEG analysis methods.

**Additional file 3.** Review history.

## Data Availability

All the source code and permuted and semi-synthetic datasets used to generated results can be found at Zenodo [44]. All the codes used to generate results can be found at GitHub via URL https://github.com/xihuimeijing/DEGs_Analysis_FDR [45]. A tutorial for identifying DEGs using the Wilcoxon rank-sum test can be found at https://rpubs.com/LiYumei/806213.
