# Supplementary material for: Exaggerated false positives by popular differential expression methods when analyzing human population samples
Source: Genome Biol. 2022 Mar 15;23:79. doi: 10.1186/s13059-022-02648-4 (PMC8922736; doi:10.1186/s13059-022-02648-4)
Supplement: Supplementary file 1 — Additional file 1. Figs. S1 to S30 and Table S1. [file 13059_2022_2648_MOESM1_ESM.pdf]

Fig. S1

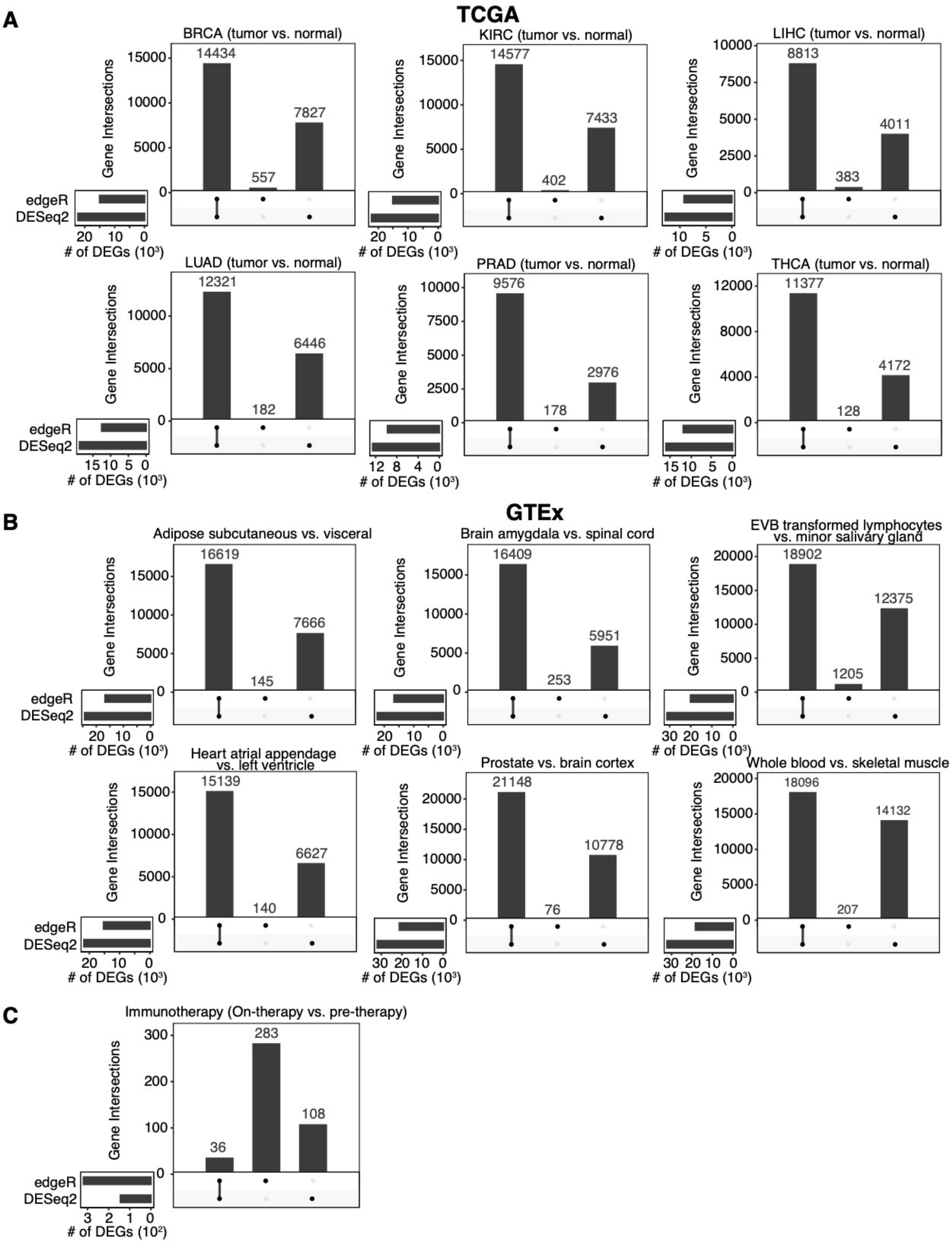
A
TCGA
BRCA (tumor vs. normal)
KIRC (tumor vs. normal)
LIHC (tumor vs. normal)
LUAD (tumor vs. normal)
PRAD (tumor vs. normal)
THCA (tumor vs. normal)
Gene Intersections
edgeR
DESeq2
# of DEGs (10³)
14434
557
7827
14577
402
7433
8813
383
4011
12321
182
6446
9576
178
2976
11377
128
4172
B
GTEx
Adipose subcutaneous vs. visceral
Brain amygdala vs. spinal cord
EVB transformed lymphocytes vs. minor salivary gland
Heart atrial appendage vs. left ventricle
Prostate vs. brain cortex
Whole blood vs. skeletal muscle
16619
145
7666
16409
253
5951
18902
1205
12375
15139
140
6627
21148
76
10778
18096
207
14132
C
Immunotherapy (On-therapy vs. pre-therapy)
36
283
108
# of DEGs (10²)

Fig. S1. The large discrepancies in the DEGs identified by DESeq2 and edgeR.

(A-C) Upset plots showing the intersections of the DEGs identified by DESeq2 and edgeR from TCGA (**A**), GTEx (**B**), and immunotherapy (**C**) RNA-seq datasets.

Fig. S2

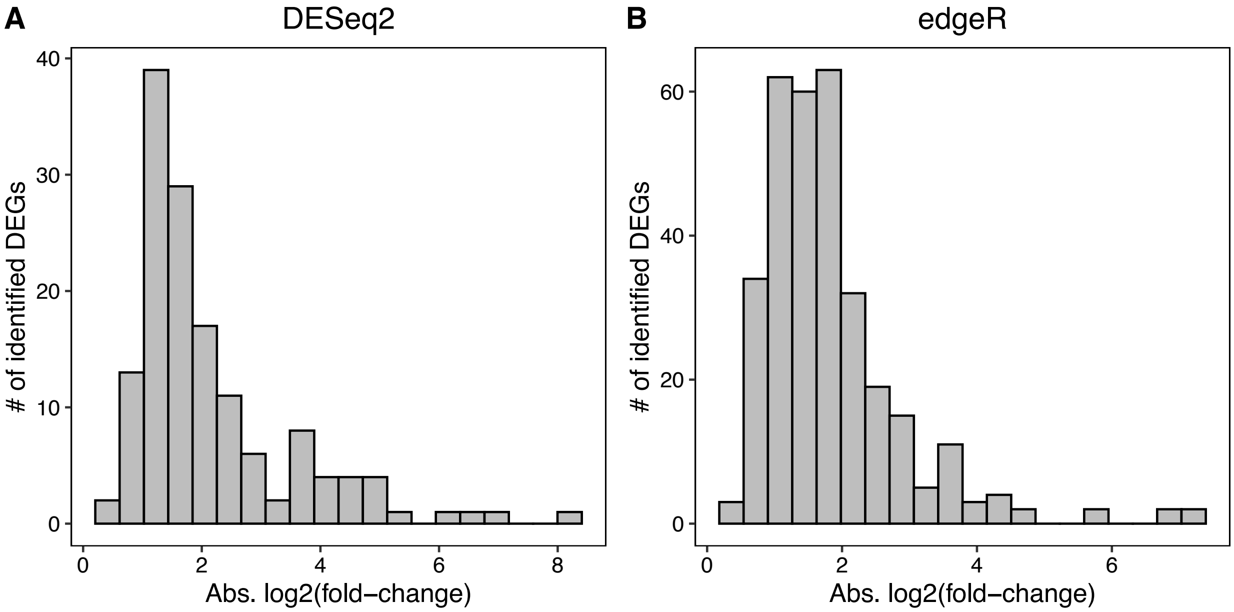

Fig. S2. The distribution of gene expression fold changes between pre-therapy and on-therapy samples in the original immunotherapy dataset calculated by DESeq2 (A) and edgeR (B).

Fig. S3

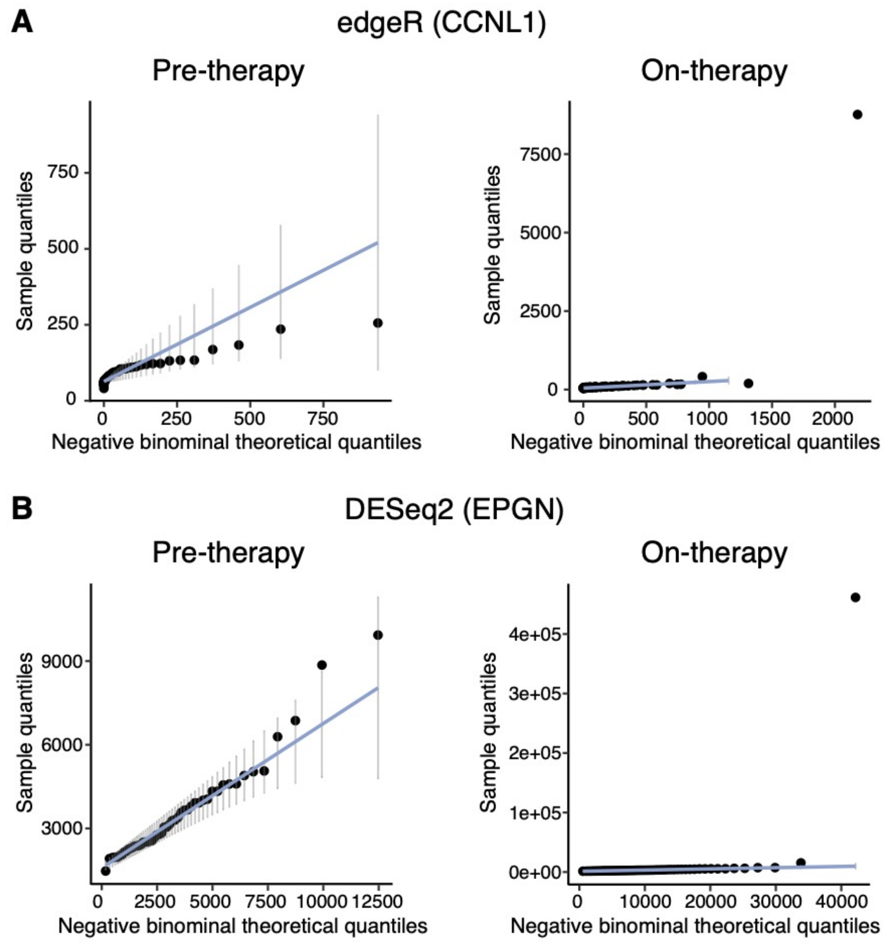

Fig. S3. Quantile-quantile (Q-Q) plots showing the discrepancy between observed read counts and the negative binominal theoretical quantiles estimated by edgeR and DESeq2.

A. The Q-Q plot for *CCNL1* with theoretical read counts in two conditions (pre-therapy and on-therapy) estimated by edgeR.

B. The Q-Q plot for *EPGN* with theoretical read counts in two conditions (pre-therapy and on-therapy) estimated by DESeq2.

Fig. S4

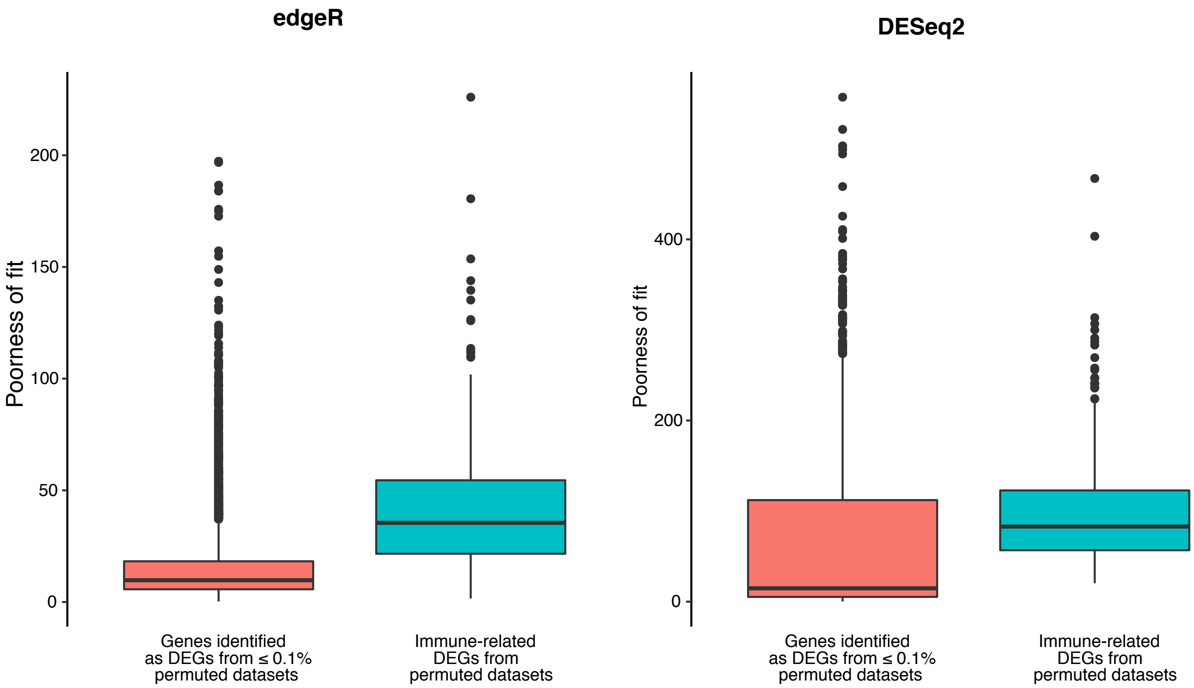

Fig. S4. Boxplots showing the poorness of fitting the negative binomial model to the immune-related spurious DEGs identified by DESeq2 or edgeR ("Immune-related DEGs from permuted datasets" in green) vs. the genes that were identified as DEGs from ≤ 0.1% permuted datasets ("Non-DEGs from permuted datasets" in red). Immune-related spurious DEGs are the genes that were falsely identified as DEGs from at least 10% of the permuted datasets and were associated with the immune-related GO terms in Fig. 1E. The poorness of fit of each gene is defined as the negative log10(p-value) from the goodness-of-fit test for the negative binomial distribution based on estimated parameters by DESeq2 or edgeR. The p-value in each panel was calculated by the Wilcoxon rank-sum test to compare the two groups of genes' poorness-of-fit values.

Fig. S5

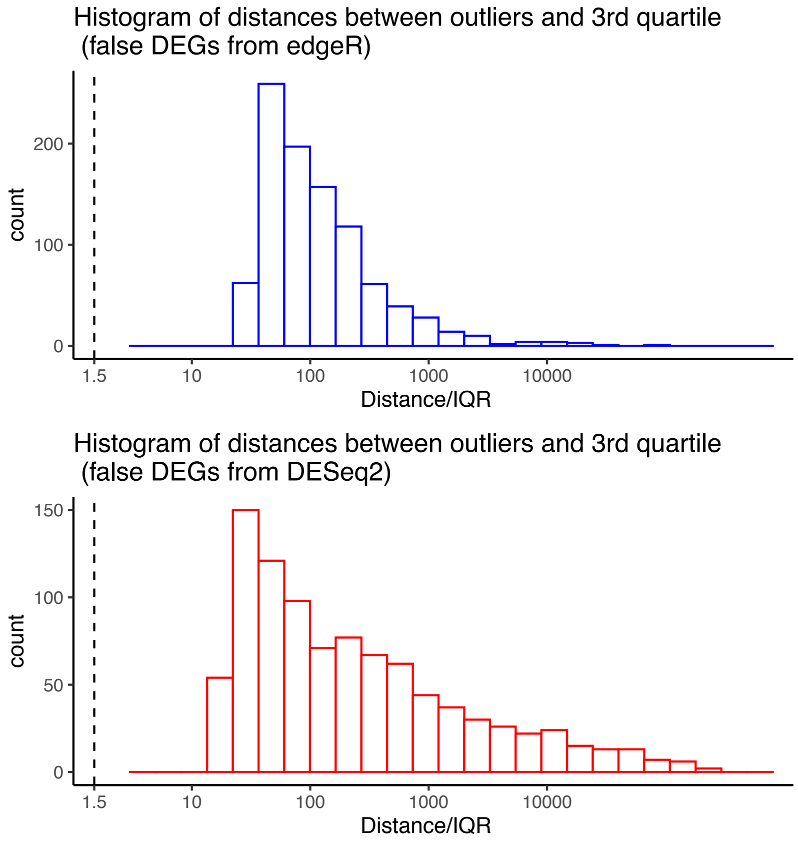

Fig. S5. Histograms showing the distances between the outliers and the third quartile of normalized gene expression values reported by edgeR (upper panel) or DESeq2 (lower panel). The results are for the genes that were falsely identified by each method as DEGs in at least 10 permuted datasets. The x-axis is (the distance between the largest outlier and the 3rd quartile of each gene) / (the inter-quartile range (IQR) of each gene).

Fig. S6

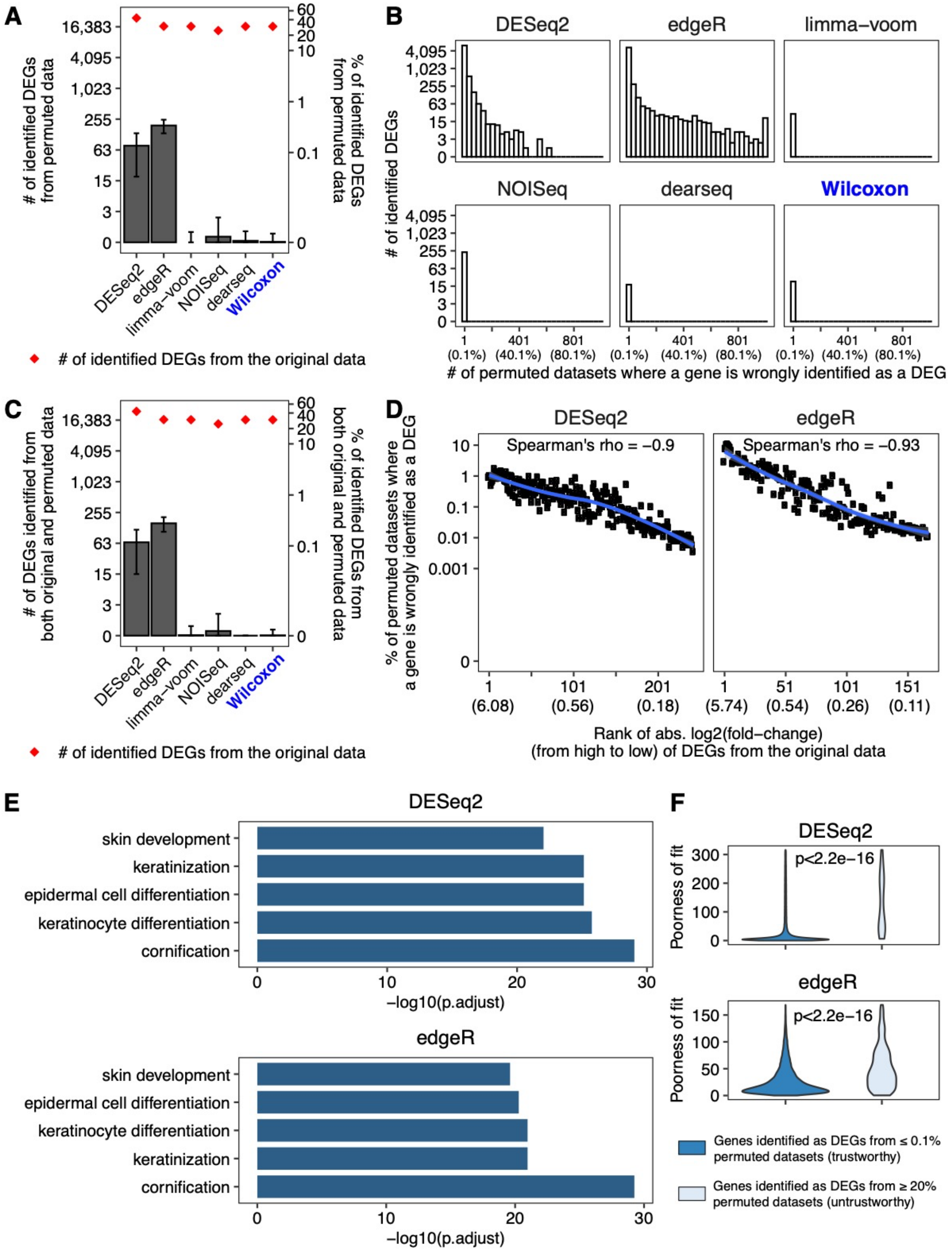
A
# of identified DEGs from permuted data
% of identified DEGs from permuted data
DESeq2
edgeR
limma−voom
NOISeq
dearseq
Wilcoxon
# of identified DEGs from the original data
B
# of identified DEGs
# of permuted datasets where a gene is wrongly identified as a DEG
C
# of DEGs identified from both original and permuted data
% of identified DEGs from both original and permuted data
D
Spearman's rho = −0.9
Spearman's rho = −0.93
% of permuted datasets where a gene is wrongly identified as a DEG
Rank of abs. log2(fold−change) (from high to low) of DEGs from the original data
E
skin development
keratinization
epidermal cell differentiation
keratinocyte differentiation
cornification
−log10(p.adjust)
F
Poorness of fit
p<2.2e−16
Genes identified as DEGs from ≤ 0.1% permuted datasets (trustworthy)
Genes identified as DEGs from ≥ 20% permuted datasets (untrustworthy)

Fig. S6. Exaggerated false DEGs identified by DESeq2 and edgeR from adipose (subcutaneous vs. visceral) GTEx RNA-seq datasets.

A. Barplot showing the average numbers of DEGs (left y-axis) and the proportion of DEGs out of all genes (right y-axis) identified from 1000 permuted datasets. The error bars represent the standard deviations of 1000 permutations. The red dots indicate the numbers of DEGs identified from the original dataset.

B. The distributions of the number of permuted datasets where a gene was mistakenly identified as a DEG. The percentages corresponding to the numbers are listed in parentheses below the numbers.

C. Barplot showing the average numbers of DEGs (left y-axis) and the proportion of DEGs out of all genes (right y-axis) identified from both the original dataset and any of the 1000 permuted datasets. The error bars represent the standard deviations of 1000 permutations. The red dots indicate the numbers of DEGs identified from the original dataset.

D. Percentage of permuted datasets where a DEG identified from the original dataset was also identified as a DEG. The genes are sorted by absolute log2(fold-change) in the original dataset in decreasing order and the average values of each 100 genes are shown. The absolute log2(fold-change) values corresponding to the ranks are listed in parentheses below the ranks. The line is fitted using the loess method, and the shaded areas represent 95% confidential intervals.

E. GO term enrichment for the DEGs identified from at least 10% permuted datasets. The top 5 enriched biological processes GO terms are shown. The analyses were performed using R package clusterProfiler. P.adjust represents the adjusted p-value using the Benjamini & Hochberg method.

F. Violin plots showing the poorness of fitting the negative binomial model to the genes identified by DESeq2 or edgeR as DEGs from ≥ 20% vs. ≤ 0.1% permuted datasets. The poorness of fit for each gene is defined as its negative $\log_{10}$(p-value) from the Pearson's chi-squared test for the negative binomial distribution. The p-value in each panel was calculated by the Wilcoxon rank-sum test to compare the two groups of genes' poorness-of-fit values.

Fig. S7

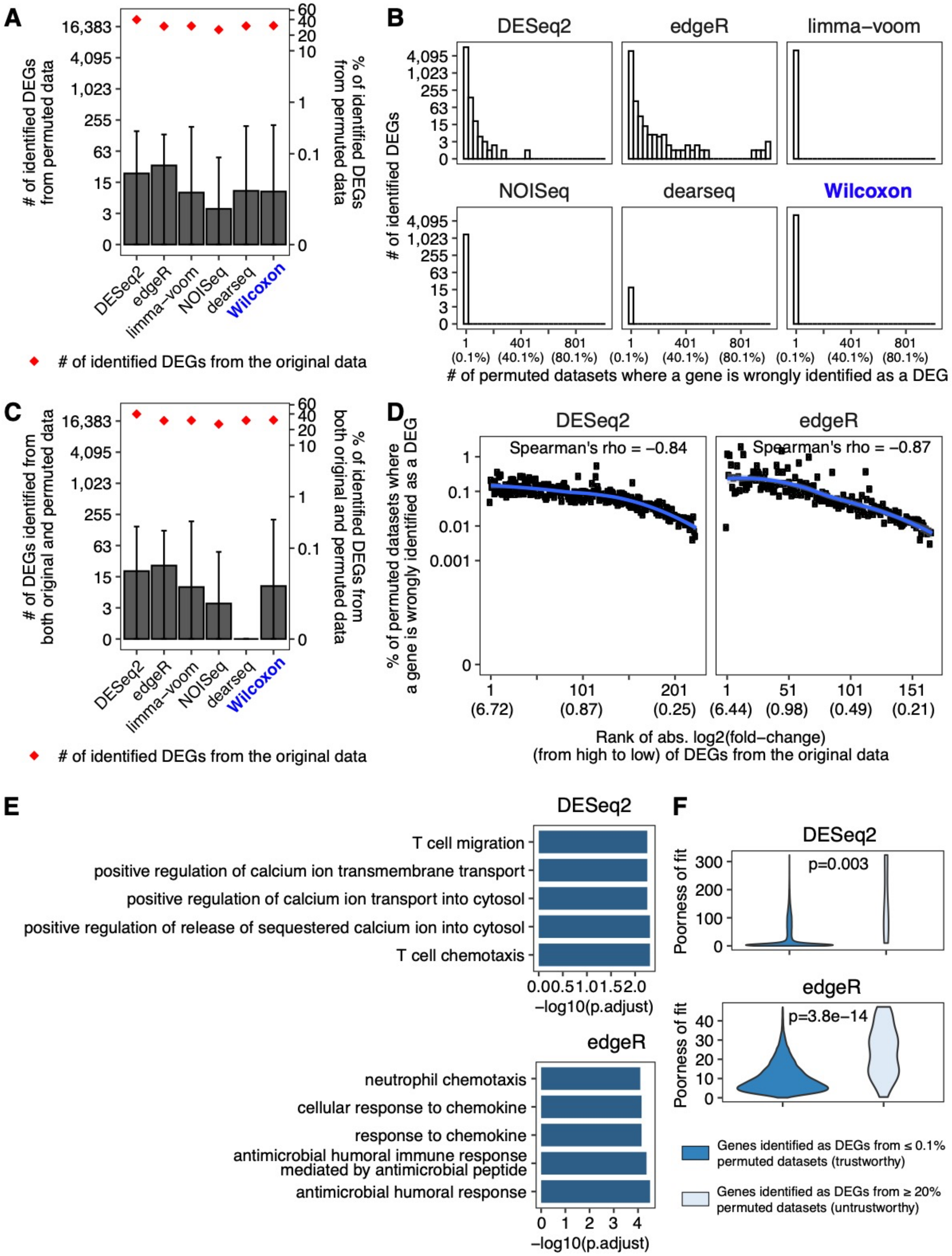

A
# of identified DEGs from permuted data
% of identified DEGs from permuted data
DESeq2
edgeR
limma−voom
NOISeq
dearseq
Wilcoxon
# of identified DEGs from the original data
B
# of identified DEGs
# of permuted datasets where a gene is wrongly identified as a DEG
C
# of DEGs identified from both original and permuted data
% of identified DEGs from both original and permuted data
D
Spearman's rho = −0.84
Spearman's rho = −0.87
% of permuted datasets where a gene is wrongly identified as a DEG
Rank of abs. log2(fold−change) (from high to low) of DEGs from the original data
E
T cell migration
positive regulation of calcium ion transmembrane transport
positive regulation of calcium ion transport into cytosol
positive regulation of release of sequestered calcium ion into cytosol
T cell chemotaxis
−log10(p.adjust)
neutrophil chemotaxis
cellular response to chemokine
response to chemokine
antimicrobial humoral immune response mediated by antimicrobial peptide
antimicrobial humoral response
F
Poorness of fit
p=0.003
p=3.8e−14
Genes identified as DEGs from ≤ 0.1% permuted datasets (trustworthy)
Genes identified as DEGs from ≥ 20% permuted datasets (untrustworthy)

Fig. S7. Exaggerated false DEGs identified by DESeq2 and edgeR from brain (amygdala vs. spinal cord) GTEx RNA-seq datasets.

A. Barplot showing the average numbers of DEGs (left y-axis) and the proportion of DEGs out of any genes (right y-axis) identified from 1000 permuted datasets. The error bars represent the standard deviations of 1000 permutations. The red dots indicate the numbers of DEGs identified from the original dataset.

B. The distributions of the number of permuted datasets where a gene was mistakenly identified as a DEG. The percentages corresponding to the numbers are listed in parentheses below the numbers.

C. Barplot showing the average numbers of DEGs (left y-axis) and the proportion of DEGs out of any genes (right y-axis) identified from both the original dataset and any of the 1000 permuted datasets. The error bars represent the standard deviations of 1000 permutations. The red dots indicate the numbers of DEGs identified from the original dataset.

D. Percentage of permuted datasets where a DEG identified from the original dataset was also identified as a DEG. The genes are sorted by absolute log2(fold-change) in the original dataset in decreasing order and the average values of each 100 genes are shown. The absolute log2(fold-change) values corresponding to the ranks are listed in parentheses below the ranks. The line is fitted using the loess method, and the shaded areas represent 95% confidential intervals.

E. GO term enrichment for the DEGs identified from at least 10% permuted datasets. The top 5 enriched biological processes GO terms are shown. The analyses were performed using R package clusterProfiler. P.adjust represents the adjusted p-value using the Benjamini & Hochberg method.

F. Violin plots showing the poorness of fitting the negative binomial model to the genes identified by DESeq2 or edgeR as DEGs from ≥ 20% vs. ≤ 0.1% permuted datasets. The poorness of fit for each gene is defined as its negative $\log_{10}$(p-value) from the Pearson's chi-squared test for the negative binomial distribution. The p-value in each panel was calculated by the Wilcoxon rank-sum test to compare the two groups of genes' poorness-of-fit values.

Fig. S8

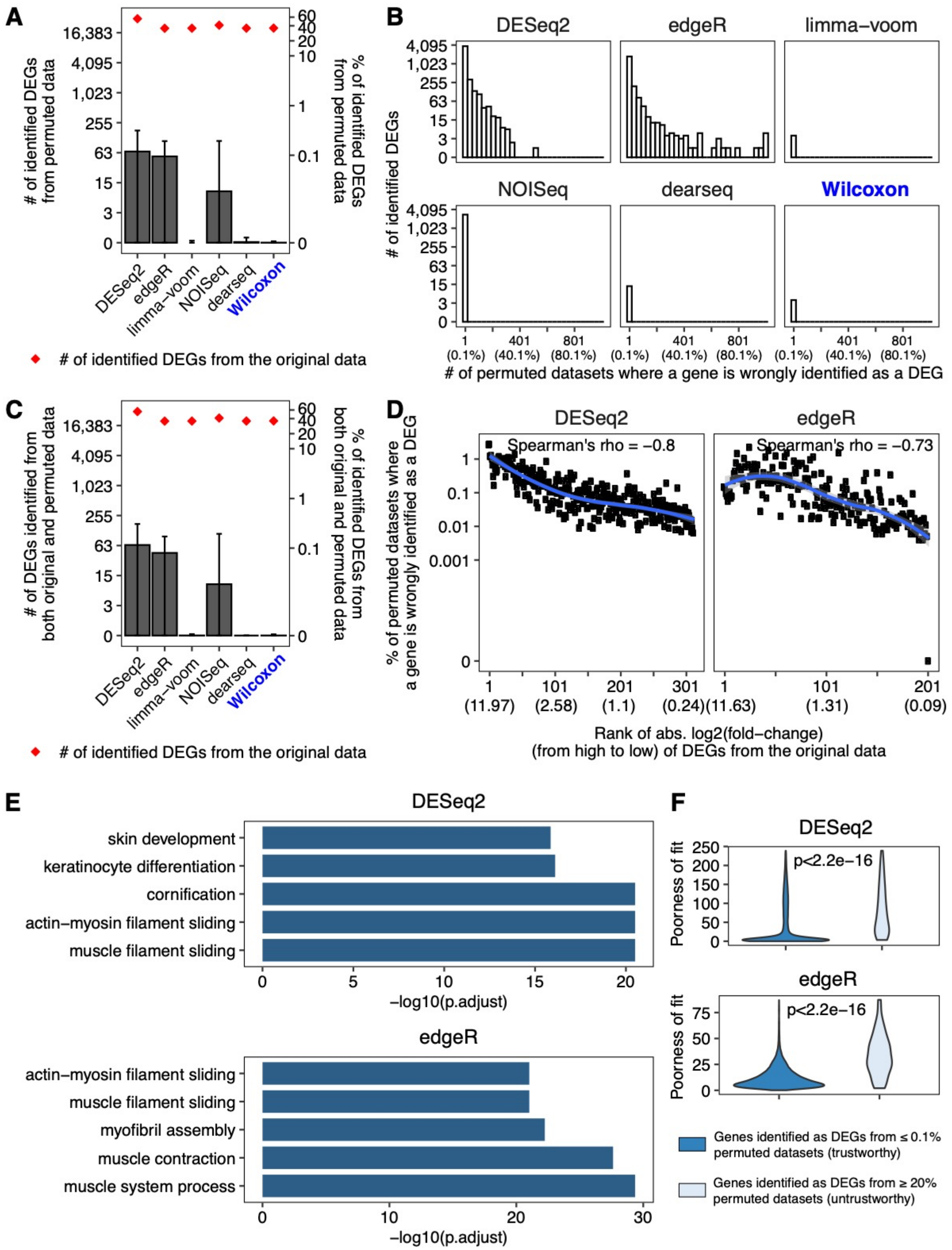
A
# of identified DEGs from permuted data
% of identified DEGs from permuted data
DESeq2
edgeR
limma−voom
NOISeq
dearseq
Wilcoxon
# of identified DEGs from the original data
B
# of identified DEGs
# of permuted datasets where a gene is wrongly identified as a DEG
C
# of DEGs identified from both original and permuted data
% of identified DEGs from both original and permuted data
D
Spearman's rho = −0.8
Spearman's rho = −0.73
% of permuted datasets where a gene is wrongly identified as a DEG
Rank of abs. log2(fold−change) (from high to low) of DEGs from the original data
E
skin development
keratinocyte differentiation
cornification
actin−myosin filament sliding
muscle filament sliding
−log10(p.adjust)
myofibril assembly
muscle contraction
muscle system process
F
Poorness of fit
p<2.2e−16
Genes identified as DEGs from ≤ 0.1% permuted datasets (trustworthy)
Genes identified as DEGs from ≥ 20% permuted datasets (untrustworthy)

Fig. S8. Exaggerated false DEGs identified by DESeq2 and edgeR from EVB transformed lymphocytes vs. minor salivary gland GTEx RNA-seq datasets.

A. Barplot showing the average numbers of DEGs (left y-axis) and the proportion of DEGs out of all genes (right y-axis) identified from 1000 permuted datasets. The error bars represent the standard deviations of 1000 permutations. The red dots indicate the numbers of DEGs identified from the original dataset.

B. The distributions of the number of permuted datasets where a gene was mistakenly identified as a DEG. The percentages corresponding to the numbers are listed in parentheses below the numbers.

C. Barplot showing the average numbers of DEGs (left y-axis) and the proportion of DEGs out of all genes (right y-axis) identified from both the original dataset and any of the 1000 permuted datasets. The error bars represent the standard deviations of 1000 permutations. The red dots indicate the numbers of DEGs identified from the original dataset.

D. Percentage of permuted datasets where a DEG identified from the original dataset was also identified as a DEG. The genes are sorted by absolute log2(fold-change) in the original dataset in decreasing order and the average values of each 100 genes are shown. The absolute log2(fold-change) values corresponding to the ranks are listed in parentheses below the ranks. The line is fitted using the loess method, and the shaded areas represent 95% confidential intervals.

E. GO term enrichment for the DEGs identified from at least 10% permuted datasets. The top 5 enriched biological processes GO terms are shown. The analyses were performed using R package clusterProfiler. P.adjust represents the adjusted p-value using the Benjamini & Hochberg method.

F. Violin plots showing the poorness of fitting the negative binomial model to the genes identified by DESeq2 or edgeR as DEGs from ≥ 20% vs. ≤ 0.1% permuted datasets. The poorness of fit for each gene is defined as its negative $\log_{10}$(p-value) from the Pearson's chi-squared test for the negative binomial distribution. The p-value in each panel was calculated by the Wilcoxon rank-sum test to compare the two groups of genes' poorness-of-fit values.

Fig. S9

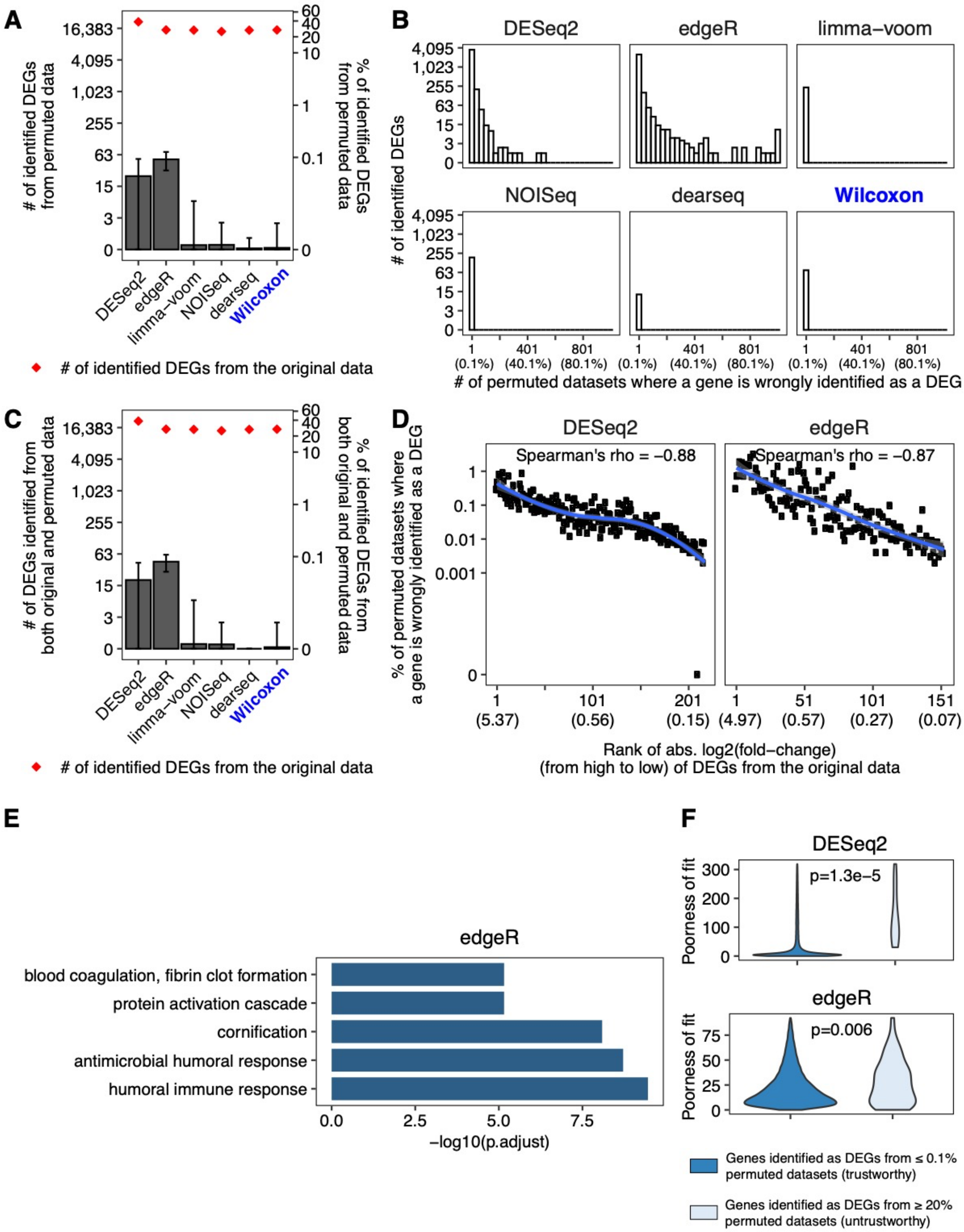

A
# of identified DEGs from permuted data
% of identified DEGs from permuted data
DESeq2
edgeR
limma−voom
NOISeq
dearseq
Wilcoxon
# of identified DEGs from the original data
B
DESeq2
edgeR
limma−voom
NOISeq
dearseq
Wilcoxon
# of identified DEGs
# of permuted datasets where a gene is wrongly identified as a DEG
C
# of DEGs identified from both original and permuted data
% of identified DEGs from both original and permuted data
# of identified DEGs from the original data
D
DESeq2
edgeR
Spearman's rho = −0.88
Spearman's rho = −0.87
% of permuted datasets where a gene is wrongly identified as a DEG
Rank of abs. log2(fold−change) (from high to low) of DEGs from the original data
E
edgeR
blood coagulation, fibrin clot formation
protein activation cascade
cornification
antimicrobial humoral response
humoral immune response
−log10(p.adjust)
F
DESeq2
p=1.3e−5
edgeR
p=0.006
Poorness of fit
Genes identified as DEGs from ≤ 0.1% permuted datasets (trustworthy)
Genes identified as DEGs from ≥ 20% permuted datasets (untrustworthy)

Fig. S9. Exaggerated false DEGs identified by DESeq2 and edgeR from heart (atrial appendage vs. left ventricle) GTEx RNA-seq datasets.

A. Barplot showing the average numbers of DEGs (left y-axis) and the proportion of DEGs out of all genes (right y-axis) identified from 1000 permuted datasets. The error bars represent the standard deviations of 1000 permutations. The red dots indicate the numbers of DEGs identified from the original dataset.

B. The distributions of the number of permuted datasets where a gene was mistakenly identified as a DEG. The percentages corresponding to the numbers are listed in parentheses below the numbers.

C. Barplot showing the average numbers of DEGs (left y-axis) and the proportion of DEGs out of all genes (right y-axis) identified from both the original dataset and any of the 1000 permuted datasets. The error bars represent the standard deviations of 1000 permutations. The red dots indicate the numbers of DEGs identified from the original dataset.

D. Percentage of permuted datasets where a DEG identified from the original dataset was also identified as a DEG. The genes are sorted by absolute log2(fold-change) in the original dataset in decreasing order and the average values of each 100 genes are shown. The absolute log2(fold-change) values corresponding to the ranks are listed in parentheses below the ranks. The line is fitted using the loess method, and the shaded areas represent 95% confidential intervals.

E. GO term enrichment for the DEGs identified from at least 10% permuted datasets. The top 5 enriched biological processes GO terms are shown. The analyses were performed using R package clusterProfiler. P.adjust represents the adjusted p-value using the Benjamini & Hochberg method.

F. Violin plots showing the poorness of fitting the negative binomial model to the genes identified by DESeq2 or edgeR as DEGs from ≥ 20% vs. ≤ 0.1% permuted datasets. The poorness of fit for each gene is defined as its negative $\log_{10}$(p-value) from the Pearson's chi-squared test for the negative binomial distribution. The p-value in each panel was calculated by the Wilcoxon rank-sum test to compare the two groups of genes' poorness-of-fit values.

Fig. S10

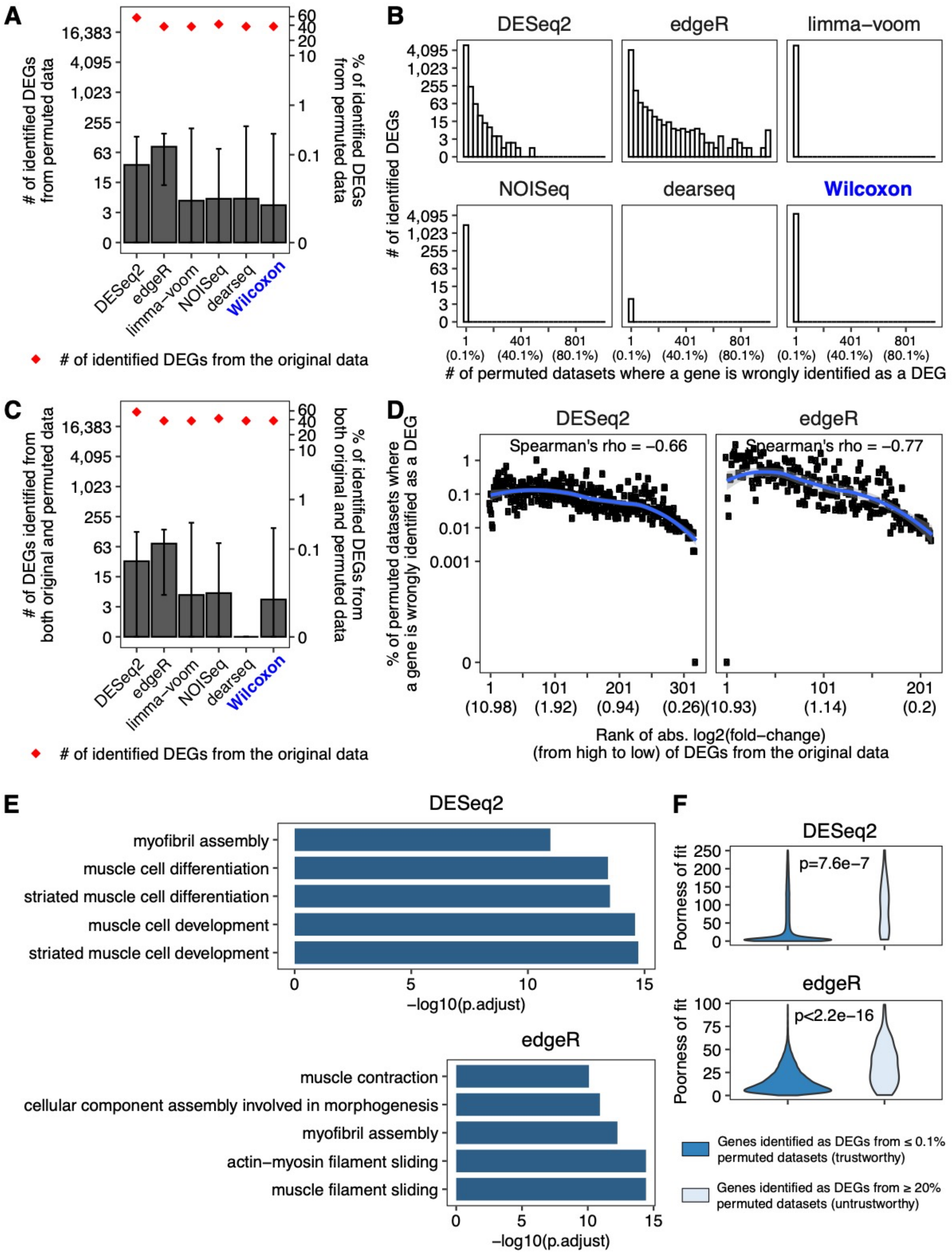
A
# of identified DEGs from permuted data
% of identified DEGs from permuted data
DESeq2
edgeR
limma−voom
NOISeq
dearseq
Wilcoxon
# of identified DEGs from the original data
B
DESeq2
edgeR
limma−voom
NOISeq
dearseq
Wilcoxon
# of identified DEGs
# of permuted datasets where a gene is wrongly identified as a DEG
C
# of DEGs identified from both original and permuted data
% of identified DEGs from both original and permuted data
# of identified DEGs from the original data
D
Spearman's rho = −0.66
Spearman's rho = −0.77
% of permuted datasets where a gene is wrongly identified as a DEG
Rank of abs. log2(fold−change) (from high to low) of DEGs from the original data
E
DESeq2
myofibril assembly
muscle cell differentiation
striated muscle cell differentiation
muscle cell development
striated muscle cell development
−log10(p.adjust)
edgeR
muscle contraction
cellular component assembly involved in morphogenesis
myofibril assembly
actin−myosin filament sliding
muscle filament sliding
−log10(p.adjust)
F
DESeq2
p=7.6e−7
edgeR
p<2.2e−16
Poorness of fit
Genes identified as DEGs from ≤ 0.1% permuted datasets (trustworthy)
Genes identified as DEGs from ≥ 20% permuted datasets (untrustworthy)

Fig. S10. Exaggerated false DEGs identified by DESeq2 and edgeR from prostate vs. brain cortex GTEx RNA-seq datasets.

A. Barplot showing the average numbers of DEGs (left y-axis) and the proportion of DEGs out of all genes (right y-axis) identified from 1000 permuted datasets. The error bars represent the standard deviations of 1000 permutations. The red dots indicate the numbers of DEGs identified from the original dataset.

B. The distributions of the number of permuted datasets where a gene was mistakenly identified as a DEG. The percentages corresponding to the numbers are listed in parentheses below the numbers.

C. Barplot showing the average numbers of DEGs (left y-axis) and the proportion of DEGs out of all genes (right y-axis) identified from both the original dataset and any of the 1000 permuted datasets. The error bars represent the standard deviations of 1000 permutations. The red dots indicate the numbers of DEGs identified from the original dataset.

D. Percentage of permuted datasets where a DEG identified from the original dataset was also identified as a DEG. The genes are sorted by absolute log2(fold-change) in the original dataset in decreasing order and the average values of each 100 genes are shown. The absolute log2(fold-change) values corresponding to the ranks are listed in parentheses below the ranks. The line is fitted using the loess method, and the shaded areas represent 95% confidential intervals.

E. GO term enrichment for the DEGs identified from at least 10% permuted datasets. The top 5 enriched biological processes GO terms are shown. The analyses were performed using R package clusterProfiler. P.adjust represents the adjusted p-value using the Benjamini & Hochberg method.

F. Violin plots showing the poorness of fitting the negative binomial model to the genes identified by DESeq2 or edgeR as DEGs from ≥ 20% vs. ≤ 0.1% permuted datasets. The poorness of fit for each gene is defined as its negative $\log_{10}$(p-value) from the Pearson's chi-squared test for the negative binomial distribution. The p-value in each panel was calculated by the Wilcoxon rank-sum test to compare the two groups of genes' poorness-of-fit values.

Fig. S11

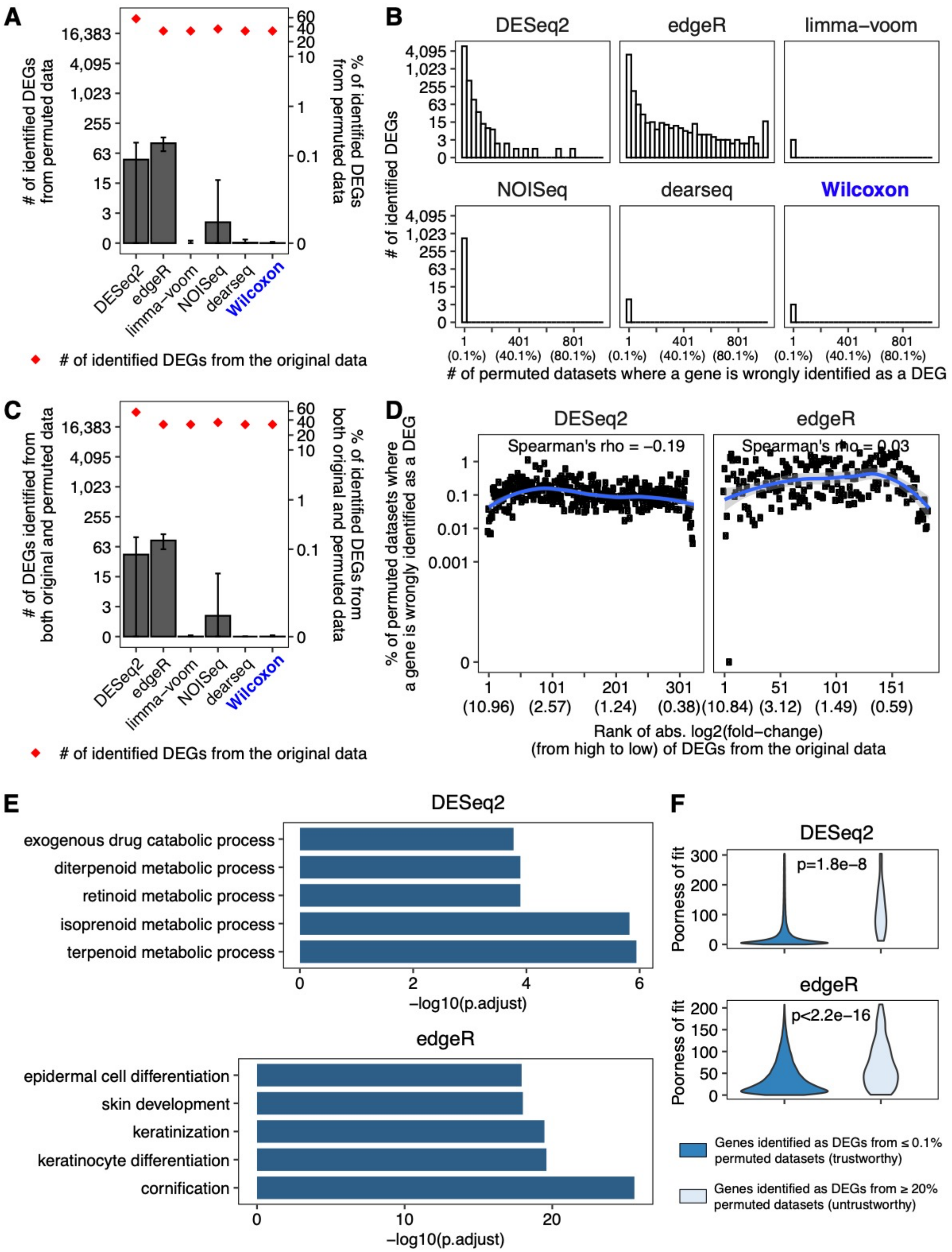
A
# of identified DEGs from permuted data
% of identified DEGs from permuted data
16,383
4,095
1,023
255
63
15
3
0
60
40
20
10
1
0.1
0
DESeq2
edgeR
limma−voom
NOISeq
dearseq
Wilcoxon
# of identified DEGs from the original data
B
DESeq2
edgeR
limma−voom
NOISeq
dearseq
Wilcoxon
# of identified DEGs
1 (0.1%)
401 (40.1%)
801 (80.1%)
# of permuted datasets where a gene is wrongly identified as a DEG
C
# of DEGs identified from both original and permuted data
% of identified DEGs from both original and permuted data
# of identified DEGs from the original data
D
Spearman's rho = −0.19
Spearman's rho = 0.03
% of permuted datasets where a gene is wrongly identified as a DEG
1
0.1
0.01
0.001
0
1 (10.96)
101 (2.57)
201 (1.24)
301 (0.38)
1 (10.84)
51 (3.12)
101 (1.49)
151 (0.59)
Rank of abs. log2(fold−change) (from high to low) of DEGs from the original data
E
DESeq2
exogenous drug catabolic process
diterpenoid metabolic process
retinoid metabolic process
isoprenoid metabolic process
terpenoid metabolic process
−log10(p.adjust)
edgeR
epidermal cell differentiation
skin development
keratinization
keratinocyte differentiation
cornification
−log10(p.adjust)
F
DESeq2
p=1.8e−8
Poorness of fit
edgeR
p<2.2e−16
Genes identified as DEGs from ≤ 0.1% permuted datasets (trustworthy)
Genes identified as DEGs from ≥ 20% permuted datasets (untrustworthy)

Fig. S11. Exaggerated false DEGs identified by DESeq2 and edgeR from whole blood vs. muscle GTEx RNA-seq datasets.

A. Barplot showing the average numbers of DEGs (left y-axis) and the proportion of DEGs out of all genes (right y-axis) identified from 1000 permuted datasets. The error bars represent the standard deviations of 1000 permutations. The red dots indicate the numbers of DEGs identified from the original dataset.

B. The distributions of the number of permuted datasets where a gene was mistakenly identified as a DEG. The percentages corresponding to the numbers are listed in parentheses below the numbers.

C. Barplot showing the average numbers of DEGs (left y-axis) and the proportion of DEGs out of all genes (right y-axis) identified from both the original dataset and any of the 1000 permuted datasets. The error bars represent the standard deviations of 1000 permutations. The red dots indicate the numbers of DEGs identified from the original dataset.

D. Percentage of permuted datasets where a DEG identified from the original dataset was also identified as a DEG. The genes are sorted by absolute log2(fold-change) in the original dataset in decreasing order and the average values of each 100 genes are shown. The absolute log2(fold-change) values corresponding to the ranks are listed in parentheses below the ranks. The line is fitted using the loess method, and the shaded areas represent 95% confidential intervals.

E. GO term enrichment for the DEGs identified from at least 10% permuted datasets. The top 5 enriched biological processes GO terms are shown. The analyses were performed using R package clusterProfiler. P.adjust represents the adjusted p-value using the Benjamini & Hochberg method.

F. Violin plots showing the poorness of fitting the negative binomial model to the genes identified by DESeq2 or edgeR as DEGs from ≥ 20% vs. ≤ 0.1% permuted datasets. The poorness of fit for each gene is defined as its negative $\log_{10}$(p-value) from the Pearson's chi-squared test for the negative binomial distribution. The p-value in each panel was calculated by the Wilcoxon rank-sum test to compare the two groups of genes' poorness-of-fit values.

Fig. S12

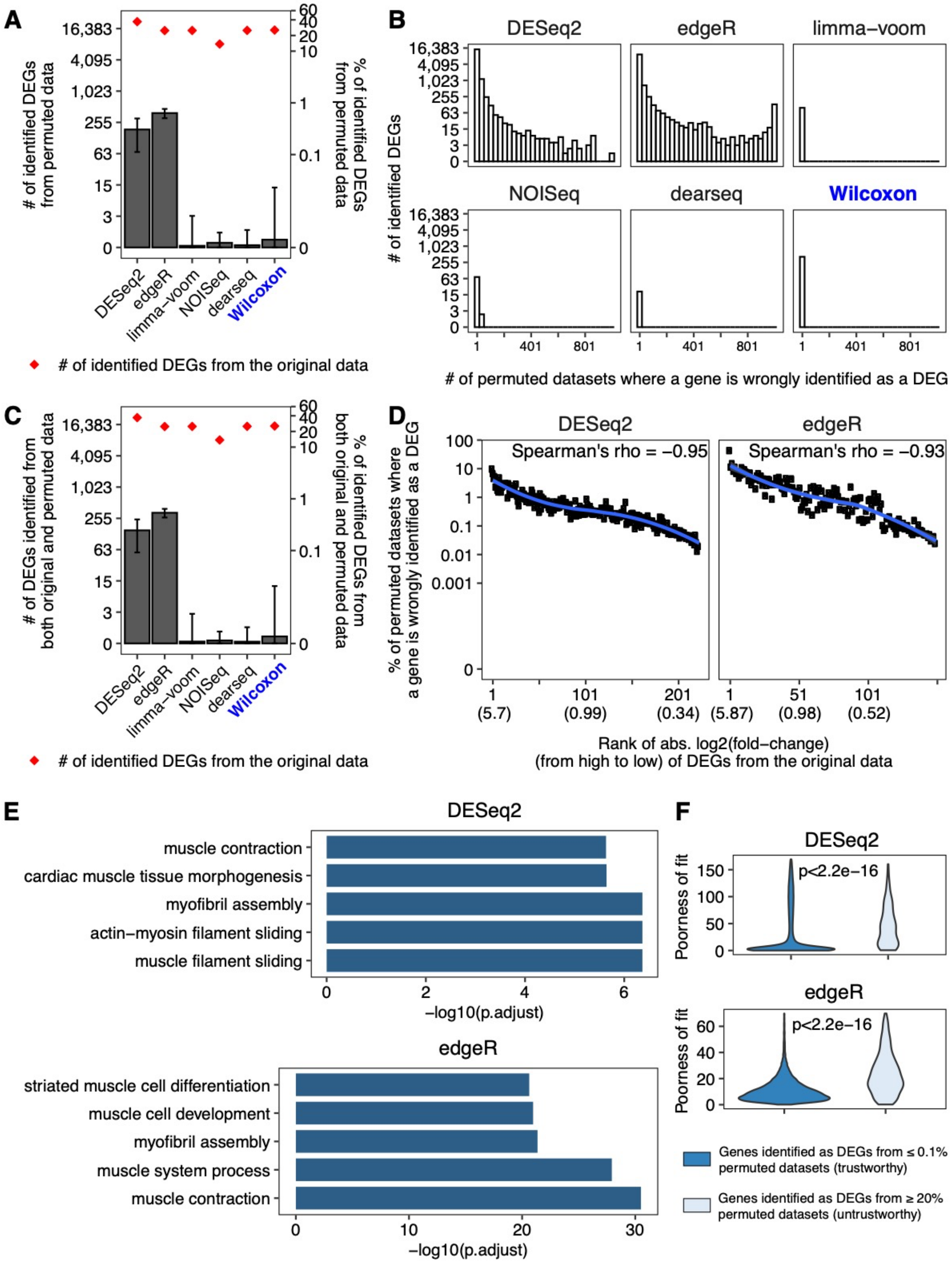

A
# of identified DEGs from permuted data
% of identified DEGs from permuted data
DESeq2
edgeR
limma−voom
NOISeq
dearseq
Wilcoxon
# of identified DEGs from the original data
B
DESeq2
edgeR
limma−voom
NOISeq
dearseq
Wilcoxon
# of identified DEGs
# of permuted datasets where a gene is wrongly identified as a DEG
C
# of DEGs identified from both original and permuted data
% of identified DEGs from both original and permuted data
# of identified DEGs from the original data
D
Spearman's rho = −0.95
Spearman's rho = −0.93
% of permuted datasets where a gene is wrongly identified as a DEG
Rank of abs. log2(fold−change) (from high to low) of DEGs from the original data
E
DESeq2
muscle contraction
cardiac muscle tissue morphogenesis
myofibril assembly
actin−myosin filament sliding
muscle filament sliding
−log10(p.adjust)
edgeR
striated muscle cell differentiation
muscle cell development
myofibril assembly
muscle system process
muscle contraction
−log10(p.adjust)
F
DESeq2
p<2.2e−16
Poorness of fit
edgeR
p<2.2e−16
Genes identified as DEGs from ≤ 0.1% permuted datasets (trustworthy)
Genes identified as DEGs from ≥ 20% permuted datasets (untrustworthy)

Fig. S12. Exaggerated false DEGs identified by DESeq2 and edgeR from BRCA (tumor vs. normal) TCGA RNA-seq datasets.

A. Barplot showing the average numbers of DEGs (left y-axis) and the proportion of DEGs out of all genes (right y-axis) identified from 1000 permuted datasets. The error bars represent the standard deviations of 1000 permutations. The red dots indicate the numbers of DEGs identified from the original dataset.

B. The distributions of the number of permuted datasets where a gene was mistakenly identified as a DEG. The percentages corresponding to the numbers are listed in parentheses below the numbers.

C. Barplot showing the average numbers of DEGs (left y-axis) and the proportion of DEGs out of all genes (right y-axis) identified from both the original dataset and any of the 1000 permuted datasets. The error bars represent the standard deviations of 1000 permutations. The red dots indicate the numbers of DEGs identified from the original dataset.

D. Percentage of permuted datasets where a DEG identified from the original dataset was also identified as a DEG. The genes are sorted by absolute log2(fold-change) in the original dataset in decreasing order and the average values of each 100 genes are shown. The absolute log2(fold-change) values corresponding to the ranks are listed in parentheses below the ranks. The line is fitted using the loess method, and the shaded areas represent 95% confidential intervals.

E. GO term enrichment for the DEGs identified from at least 10% permuted datasets. The top 5 enriched biological processes GO terms are shown. The analyses were performed using R package clusterProfiler. P.adjust represents the adjusted p-value using the Benjamini & Hochberg method.

F. Violin plots showing the poorness of fitting the negative binomial model to the genes identified by DESeq2 or edgeR as DEGs from ≥ 20% vs. ≤ 0.1% permuted datasets. The poorness of fit for each gene is defined as its negative $\log_{10}$(p-value) from the Pearson's chi-squared test for the negative binomial distribution. The p-value in each panel was calculated by the Wilcoxon rank-sum test to compare the two groups of genes' poorness-of-fit values.

Fig. S13

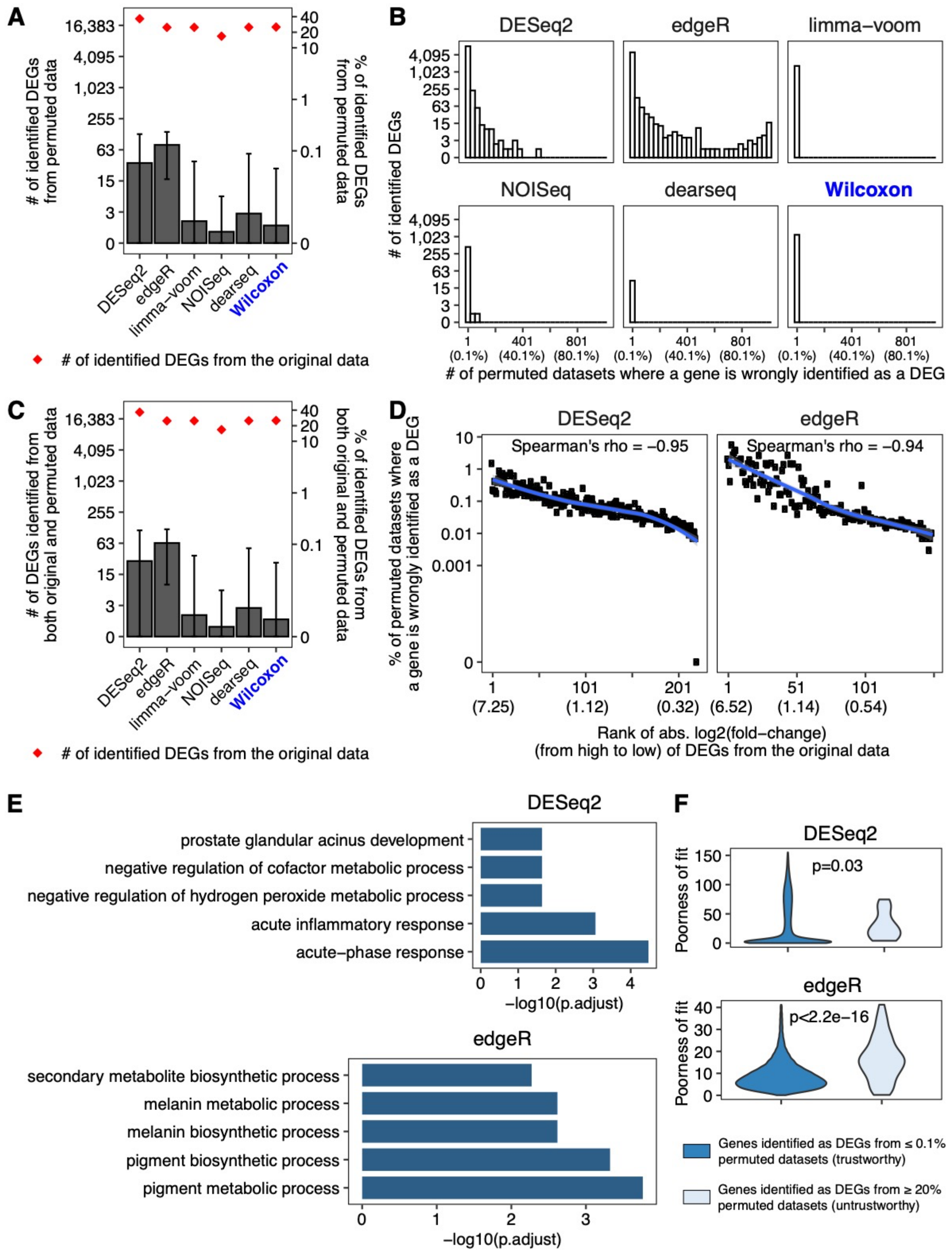

A
16,383
4,095
1,023
255
63
15
3
0
# of identified DEGs from permuted data
% of identified DEGs from permuted data
40
20
10
1
0.1
0
DESeq2
edgeR
limma−voom
NOISeq
dearseq
Wilcoxon
# of identified DEGs from the original data
B
DESeq2
edgeR
limma−voom
NOISeq
dearseq
Wilcoxon
# of identified DEGs
1 (0.1%)
401 (40.1%)
801 (80.1%)
# of permuted datasets where a gene is wrongly identified as a DEG
C
# of DEGs identified from both original and permuted data
% of identified DEGs from both original and permuted data
# of identified DEGs from the original data
D
DESeq2
edgeR
Spearman's rho = −0.95
Spearman's rho = −0.94
% of permuted datasets where a gene is wrongly identified as a DEG
10
1
0.1
0.01
0.001
0
1 (7.25)
101 (1.12)
201 (0.32)
1 (6.52)
51 (1.14)
101 (0.54)
Rank of abs. log2(fold−change) (from high to low) of DEGs from the original data
E
DESeq2
prostate glandular acinus development
negative regulation of cofactor metabolic process
negative regulation of hydrogen peroxide metabolic process
acute inflammatory response
acute−phase response
−log10(p.adjust)
edgeR
secondary metabolite biosynthetic process
melanin metabolic process
melanin biosynthetic process
pigment biosynthetic process
pigment metabolic process
−log10(p.adjust)
F
DESeq2
p=0.03
Poorness of fit
edgeR
p<2.2e−16
Genes identified as DEGs from ≤ 0.1% permuted datasets (trustworthy)
Genes identified as DEGs from ≥ 20% permuted datasets (untrustworthy)

Fig. S13. Exaggerated false DEGs identified by DESeq2 and edgeR from KIRC (tumor vs. normal) TCGA RNA-seq datasets.

A. Barplot showing the average numbers of DEGs (left y-axis) and the proportion of DEGs out of all genes (right y-axis) identified from 1000 permuted datasets. The error bars represent the standard deviations of 1000 permutations. The red dots indicate the numbers of DEGs identified from the original dataset.

B. The distributions of the number of permuted datasets where a gene was mistakenly identified as a DEG. The percentages corresponding to the numbers are listed in parentheses below the numbers.

C. Barplot showing the average numbers of DEGs (left y-axis) and the proportion of DEGs out of all genes (right y-axis) identified from both the original dataset and any of the 1000 permuted datasets. The error bars represent the standard deviations of 1000 permutations. The red dots indicate the numbers of DEGs identified from the original dataset.

D. Percentage of permuted datasets where a DEG identified from the original dataset was also identified as a DEG. The genes are sorted by absolute log2(fold-change) in the original dataset in decreasing order and the average values of each 100 genes are shown. The absolute log2(fold-change) values corresponding to the ranks are listed in parentheses below the ranks. The line is fitted using the loess method, and the shaded areas represent 95% confidential intervals.

E. GO term enrichment for the DEGs identified from at least 10% permuted datasets. The top 5 enriched biological processes GO terms are shown. The analyses were performed using R package clusterProfiler. P.adjust represents the adjusted p-value using the Benjamini & Hochberg method.

F. Violin plots showing the poorness of fitting the negative binomial model to the genes identified by DESeq2 or edgeR as DEGs from ≥ 20% vs. ≤ 0.1% permuted datasets. The poorness of fit for each gene is defined as its negative $\log_{10}$(p-value) from the Pearson's chi-squared test for the negative binomial distribution. The p-value in each panel was calculated by the Wilcoxon rank-sum test to compare the two groups of genes' poorness-of-fit values.

Fig. S14

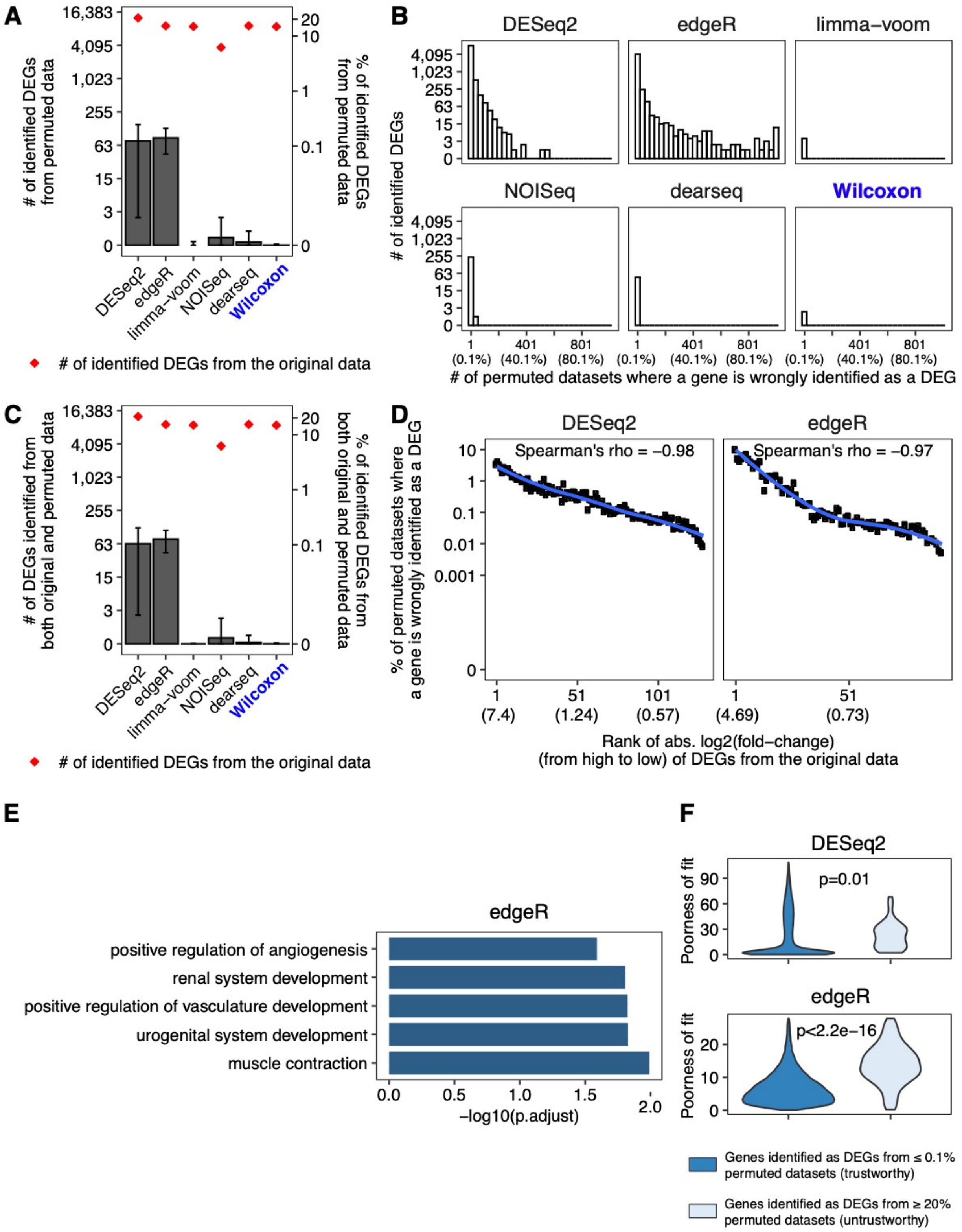
A
B
C
D
E
F
# of identified DEGs from permuted data
% of identified DEGs from permuted data
DESeq2
edgeR
limma−voom
NOISeq
dearseq
Wilcoxon
# of identified DEGs from the original data
# of permuted datasets where a gene is wrongly identified as a DEG
# of DEGs identified from both original and permuted data
% of identified DEGs from both original and permuted data
% of permuted datasets where a gene is wrongly identified as a DEG
Spearman's rho = −0.98
Spearman's rho = −0.97
Rank of abs. log2(fold−change) (from high to low) of DEGs from the original data
positive regulation of angiogenesis
renal system development
positive regulation of vasculature development
urogenital system development
muscle contraction
−log10(p.adjust)
Poorness of fit
p=0.01
p<2.2e−16
Genes identified as DEGs from ≤ 0.1% permuted datasets (trustworthy)
Genes identified as DEGs from ≥ 20% permuted datasets (untrustworthy)

Fig. S14. Exaggerated false DEGs identified by DESeq2 and edgeR from LIHC (tumor vs. normal) TCGA RNA-seq datasets.

A. Barplot showing the average numbers of DEGs (left y-axis) and the proportion of DEGs out of all genes (right y-axis) identified from 1000 permuted datasets. The error bars represent the standard deviations of 1000 permutations. The red dots indicate the numbers of DEGs identified from the original dataset.

B. The distributions of the number of permuted datasets where a gene was mistakenly identified as a DEG. The percentages corresponding to the numbers are listed in parentheses below the numbers.

C. Barplot showing the average numbers of DEGs (left y-axis) and the proportion of DEGs out of all genes (right y-axis) identified from both the original dataset and any of the 1000 permuted datasets. The error bars represent the standard deviations of 1000 permutations. The red dots indicate the numbers of DEGs identified from the original dataset.

D. Percentage of permuted datasets where a DEG identified from the original dataset was also identified as a DEG. The genes are sorted by absolute log2(fold-change) in the original dataset in decreasing order and the average values of each 100 genes are shown. The absolute log2(fold-change) values corresponding to the ranks are listed in parentheses below the ranks. The line is fitted using the loess method, and the shaded areas represent 95% confidential intervals.

E. GO term enrichment for the DEGs identified from at least 10% permuted datasets. The top 5 enriched biological processes GO terms are shown. The analyses were performed using R package clusterProfiler. P.adjust represents the adjusted p-value using the Benjamini & Hochberg method.

F. Violin plots showing the poorness of fitting the negative binomial model to the genes identified by DESeq2 or edgeR as DEGs from ≥ 20% vs. ≤ 0.1% permuted datasets. The poorness of fit for each gene is defined as its negative $\log_{10}$(p-value) from the Pearson's chi-squared test for the negative binomial distribution. The p-value in each panel was calculated by the Wilcoxon rank-sum test to compare the two groups of genes' poorness-of-fit values.

Fig. S15

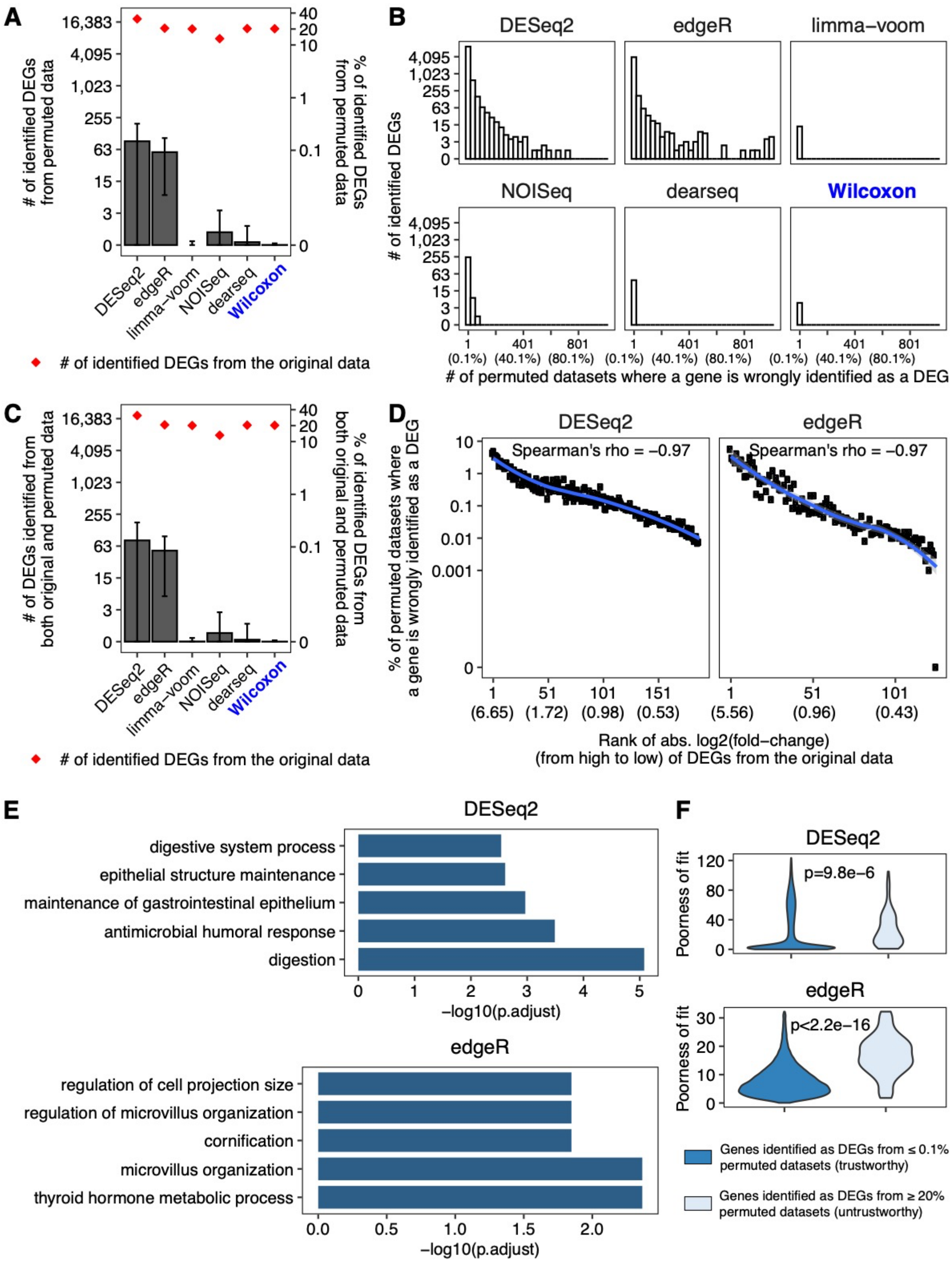
A
# of identified DEGs from permuted data
% of identified DEGs from permuted data
DESeq2
edgeR
limma−voom
NOISeq
dearseq
Wilcoxon
# of identified DEGs from the original data
B
# of identified DEGs
limma−voom
# of permuted datasets where a gene is wrongly identified as a DEG
C
# of DEGs identified from both original and permuted data
% of identified DEGs from both original and permuted data
D
Spearman's rho = −0.97
% of permuted datasets where a gene is wrongly identified as a DEG
Rank of abs. log2(fold−change) (from high to low) of DEGs from the original data
E
digestive system process
epithelial structure maintenance
maintenance of gastrointestinal epithelium
antimicrobial humoral response
digestion
−log10(p.adjust)
regulation of cell projection size
regulation of microvillus organization
cornification
microvillus organization
thyroid hormone metabolic process
F
Poorness of fit
p=9.8e−6
p<2.2e−16
Genes identified as DEGs from ≤ 0.1% permuted datasets (trustworthy)
Genes identified as DEGs from ≥ 20% permuted datasets (untrustworthy)

Fig. S15. Exaggerated false DEGs identified by DESeq2 and edgeR from LUAD (tumor vs. normal) TCGA RNA-seq datasets.

A. Barplot showing the average numbers of DEGs (left y-axis) and the proportion of DEGs out of all genes (right y-axis) identified from 1000 permuted datasets. The error bars represent the standard deviations of 1000 permutations. The red dots indicate the numbers of DEGs identified from the original dataset.

B. The distributions of the number of permuted datasets where a gene was mistakenly identified as a DEG. The percentages corresponding to the numbers are listed in parentheses below the numbers.

C. Barplot showing the average numbers of DEGs (left y-axis) and the proportion of DEGs out of all genes (right y-axis) identified from both the original dataset and any of the 1000 permuted datasets. The error bars represent the standard deviations of 1000 permutations. The red dots indicate the numbers of DEGs identified from the original dataset.

D. Percentage of permuted datasets where a DEG identified from the original dataset was also identified as a DEG. The genes are sorted by absolute log2(fold-change) in the original dataset in decreasing order and the average values of each 100 genes are shown. The absolute log2(fold-change) values corresponding to the ranks are listed in parentheses below the ranks. The line is fitted using the loess method, and the shaded areas represent 95% confidential intervals.

E. GO term enrichment for the DEGs identified from at least 10% permuted datasets. The top 5 enriched biological processes GO terms are shown. The analyses were performed using R package clusterProfiler. P.adjust represents the adjusted p-value using the Benjamini & Hochberg method.

F. Violin plots showing the poorness of fitting the negative binomial model to the genes identified by DESeq2 or edgeR as DEGs from ≥ 20% vs. ≤ 0.1% permuted datasets. The poorness of fit for each gene is defined as its negative $\log_{10}$(p-value) from the Pearson's chi-squared test for the negative binomial distribution. The p-value in each panel was calculated by the Wilcoxon rank-sum test to compare the two groups of genes' poorness-of-fit values.

Fig. S16

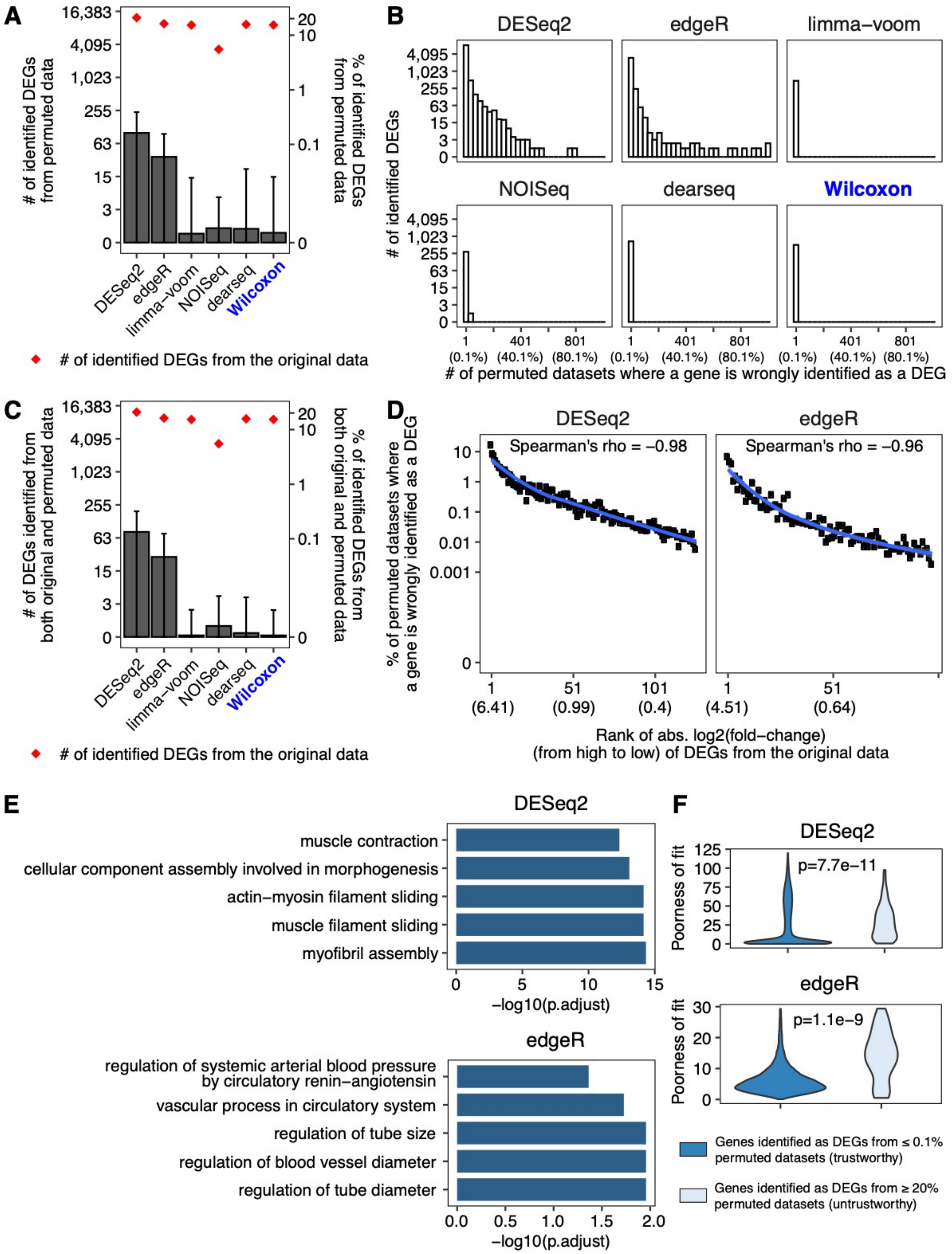

A
# of identified DEGs from permuted data
% of identified DEGs from permuted data
DESeq2
edgeR
limma−voom
NOISeq
dearseq
Wilcoxon
# of identified DEGs from the original data
B
DESeq2
edgeR
limma−voom
NOISeq
dearseq
Wilcoxon
# of identified DEGs
# of permuted datasets where a gene is wrongly identified as a DEG
C
# of DEGs identified from both original and permuted data
% of identified DEGs from both original and permuted data
# of identified DEGs from the original data
D
DESeq2
edgeR
Spearman's rho = −0.98
Spearman's rho = −0.96
% of permuted datasets where a gene is wrongly identified as a DEG
Rank of abs. log2(fold−change) (from high to low) of DEGs from the original data
E
DESeq2
muscle contraction
cellular component assembly involved in morphogenesis
actin−myosin filament sliding
muscle filament sliding
myofibril assembly
−log10(p.adjust)
edgeR
regulation of systemic arterial blood pressure by circulatory renin−angiotensin
vascular process in circulatory system
regulation of tube size
regulation of blood vessel diameter
regulation of tube diameter
−log10(p.adjust)
F
DESeq2
p=7.7e−11
Poorness of fit
edgeR
p=1.1e−9
Poorness of fit
Genes identified as DEGs from ≤ 0.1% permuted datasets (trustworthy)
Genes identified as DEGs from ≥ 20% permuted datasets (untrustworthy)

Fig. S16. Exaggerated false DEGs identified by DESeq2 and edgeR from PRAD (tumor vs. normal) TCGA RNA-seq datasets.

A. Barplot showing the average numbers of DEGs (left y-axis) and the proportion of DEGs out of all genes (right y-axis) identified from 1000 permuted datasets. The error bars represent the standard deviations of 1000 permutations. The red dots indicate the numbers of DEGs identified from the original dataset.

B. The distributions of the number of permuted datasets where a gene was mistakenly identified as a DEG. The percentages corresponding to the numbers are listed in parentheses below the numbers.

C. Barplot showing the average numbers of DEGs (left y-axis) and the proportion of DEGs out of all genes (right y-axis) identified from both the original dataset and any of the 1000 permuted datasets. The error bars represent the standard deviations of 1000 permutations. The red dots indicate the numbers of DEGs identified from the original dataset.

D. Percentage of permuted datasets where a DEG identified from the original dataset was also identified as a DEG. The genes are sorted by absolute log2(fold-change) in the original dataset in decreasing order and the average values of each 100 genes are shown. The absolute log2(fold-change) values corresponding to the ranks are listed in parentheses below the ranks. The line is fitted using the loess method, and the shaded areas represent 95% confidential intervals.

E. GO term enrichment for the DEGs identified from at least 10% permuted datasets. The top 5 enriched biological processes GO terms are shown. The analyses were performed using R package clusterProfiler. P.adjust represents the adjusted p-value using the Benjamini & Hochberg method.

F. Violin plots showing the poorness of fitting the negative binomial model to the genes identified by DESeq2 or edgeR as DEGs from ≥ 20% vs. ≤ 0.1% permuted datasets. The poorness of fit for each gene is defined as its negative $\log_{10}$(p-value) from the Pearson's chi-squared test for the negative binomial distribution. The p-value in each panel was calculated by the Wilcoxon rank-sum test to compare the two groups of genes' poorness-of-fit values.

Fig. S17

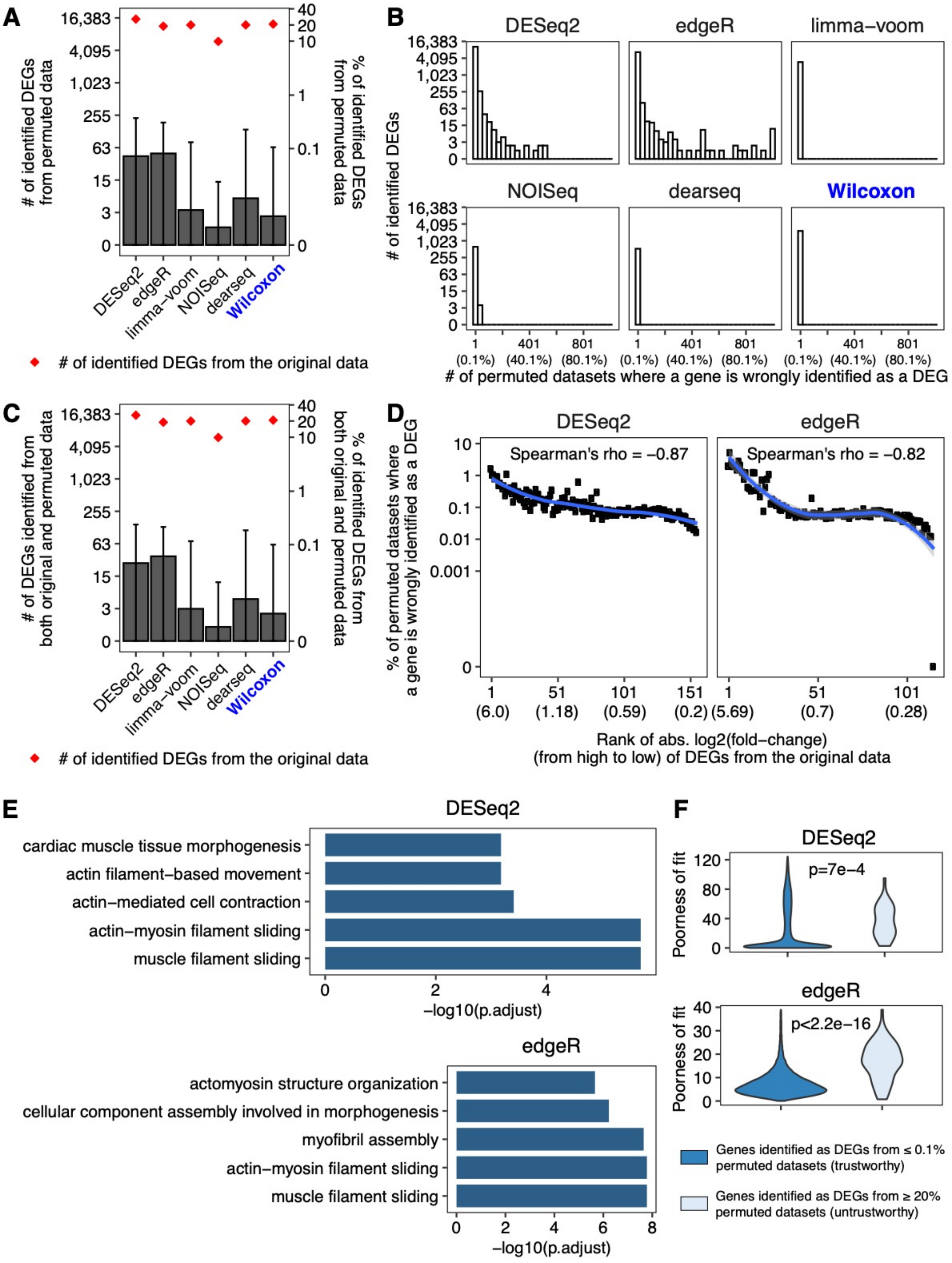
A
# of identified DEGs from permuted data
% of identified DEGs from permuted data
DESeq2
edgeR
limma−voom
NOISeq
dearseq
Wilcoxon
# of identified DEGs from the original data
B
DESeq2
edgeR
limma−voom
NOISeq
dearseq
Wilcoxon
# of identified DEGs
# of permuted datasets where a gene is wrongly identified as a DEG
C
# of DEGs identified from both original and permuted data
% of identified DEGs from both original and permuted data
# of identified DEGs from the original data
D
Spearman's rho = −0.87
Spearman's rho = −0.82
% of permuted datasets where a gene is wrongly identified as a DEG
Rank of abs. log2(fold−change) (from high to low) of DEGs from the original data
E
DESeq2
cardiac muscle tissue morphogenesis
actin filament−based movement
actin−mediated cell contraction
actin−myosin filament sliding
muscle filament sliding
−log10(p.adjust)
edgeR
actomyosin structure organization
cellular component assembly involved in morphogenesis
myofibril assembly
actin−myosin filament sliding
muscle filament sliding
−log10(p.adjust)
F
DESeq2
p=7e−4
Poorness of fit
edgeR
p<2.2e−16
Genes identified as DEGs from ≤ 0.1% permuted datasets (trustworthy)
Genes identified as DEGs from ≥ 20% permuted datasets (untrustworthy)

Fig. S17. Exaggerated false DEGs identified by DESeq2 and edgeR from THCA (tumor vs. normal) TCGA RNA-seq datasets.

A. Barplot showing the average numbers of DEGs (left y-axis) and the proportion of DEGs out of all genes (right y-axis) identified from 1000 permuted datasets. The error bars represent the standard deviations of 1000 permutations. The red dots indicate the numbers of DEGs identified from the original dataset.

B. The distributions of the number of permuted datasets where a gene was mistakenly identified as a DEG. The percentages corresponding to the numbers are listed in parentheses below the numbers.

C. Barplot showing the average numbers of DEGs (left y-axis) and the proportion of DEGs out of all genes (right y-axis) identified from both the original dataset and any of the 1000 permuted datasets. The error bars represent the standard deviations of 1000 permutations. The red dots indicate the numbers of DEGs identified from the original dataset.

D. Percentage of permuted datasets where a DEG identified from the original dataset was also identified as a DEG. The genes are sorted by absolute log2(fold-change) in the original dataset in decreasing order and the average values of each 100 genes are shown. The absolute log2(fold-change) values corresponding to the ranks are listed in parentheses below the ranks. The line is fitted using the loess method, and the shaded areas represent 95% confidential intervals.

E. GO term enrichment for the DEGs identified from at least 10% permuted datasets. The top 5 enriched biological processes GO terms are shown. The analyses were performed using R package clusterProfiler. P.adjust represents the adjusted p-value using the Benjamini & Hochberg method.

F. Violin plots showing the poorness of fitting the negative binomial model to the genes identified by DESeq2 or edgeR as DEGs from ≥ 20% vs. ≤ 0.1% permuted datasets. The poorness of fit for each gene is defined as its negative $\log_{10}$(p-value) from the Pearson's chi-squared test for the negative binomial distribution. The p-value in each panel was calculated by the Wilcoxon rank-sum test to compare the two groups of genes' poorness-of-fit values.

Fig. S18

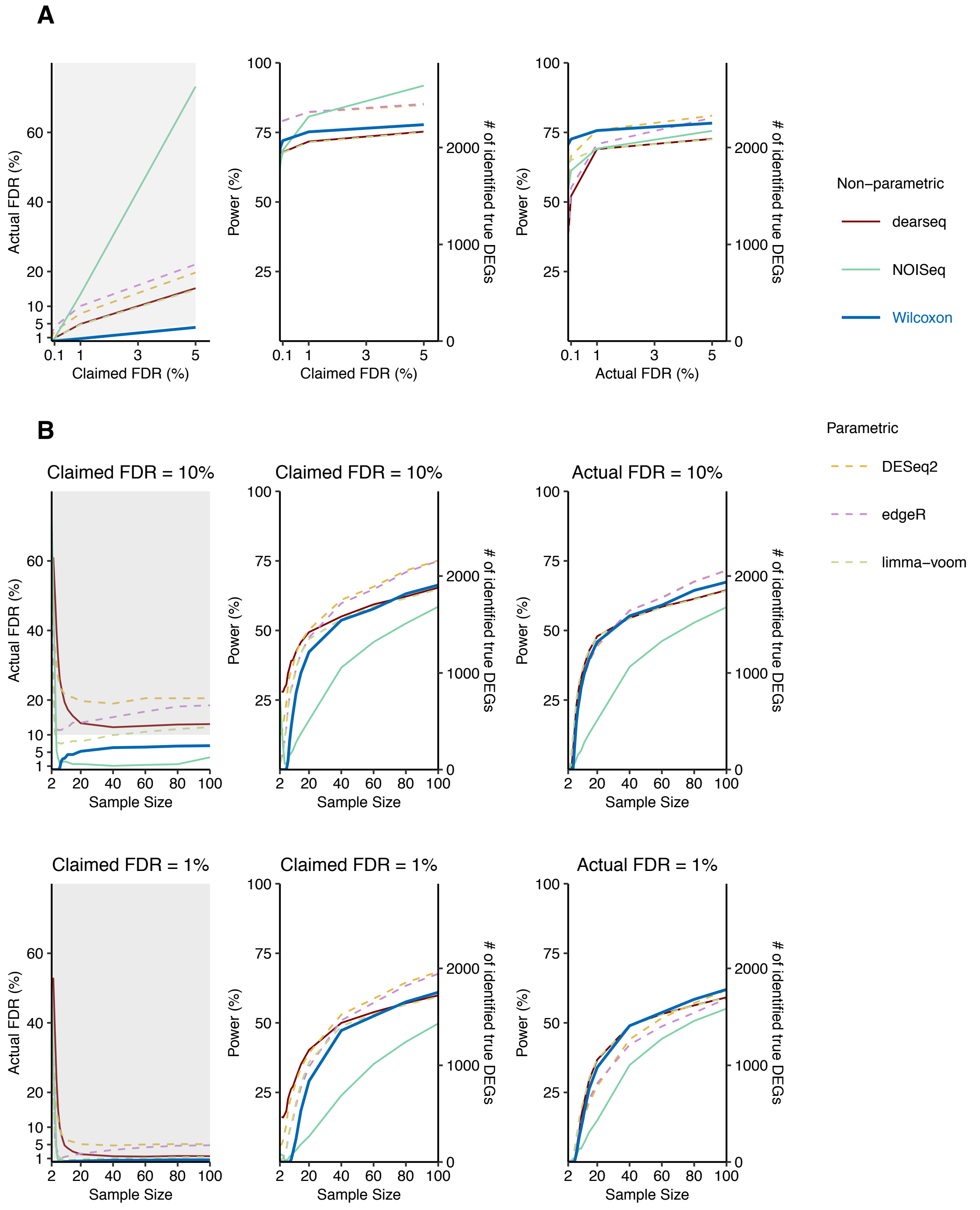

Fig. S18. The Wilcoxon rank-sum test has the best FDR control and power on heart left ventricle vs. atrial appendage GTEx datasets with semi-synthetic ground truths.

A. The FDR control (left panel), power (middle panel) given the claimed FDRs, and power given the actual FDRs (right panel) under a range of FDR thresholds from 0.001% to 5%.

B. The FDR control (left), power given the claimed FDRs (middle), and power given the actual FDRs (right) for a range of per-condition sample sizes from 2 to 100, under FDR thresholds 10% (top panels) and 1% (bottom panels). The claimed FDRs, actual FDRs, and power were all calculated as the averages of 50 randomly down-sampled datasets.

Fig. S19

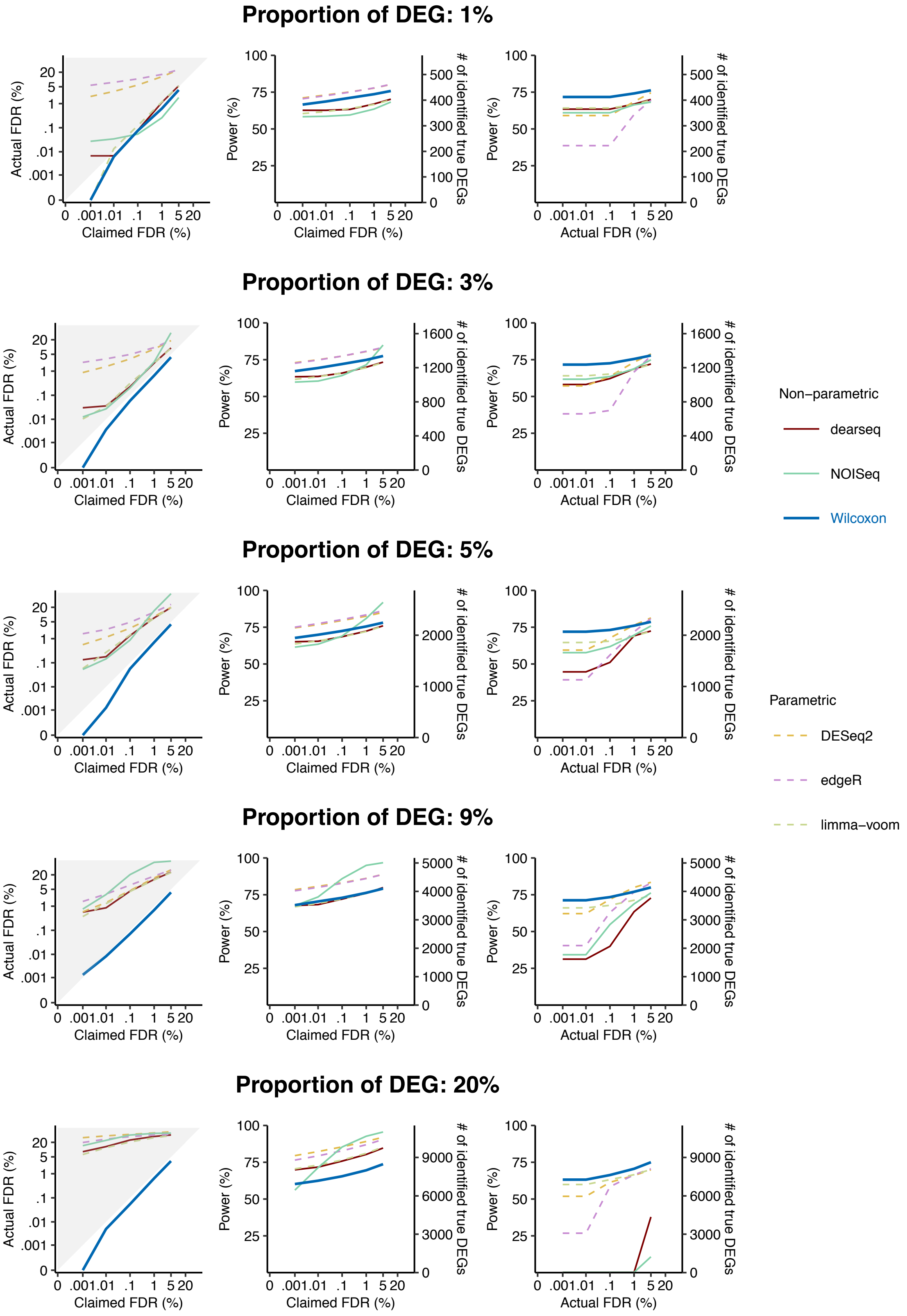

Proportion of DEG: 1%
Proportion of DEG: 3%
Proportion of DEG: 5%
Proportion of DEG: 9%
Proportion of DEG: 20%
Actual FDR (%)
Claimed FDR (%)
Power (%)
# of identified true DEGs
Actual FDR (%)
Non−parametric
dearseq
NOISeq
Wilcoxon
Parametric
DESeq2
edgeR
limma−voom

Fig. S19. The Wilcoxon rank-sum test has the best FDR control and power on heart left ventricle vs. atrial appendage GTEx datasets with semi-synthetic ground truths of varying proportions of DEGs.

The FDR control (left panel), power (middle panel) given the claimed FDRs, and power given the actual FDRs (right panel) under a range of FDR thresholds from 0.001% to 5% for semi-synthetic datasets with five proportions of DEGs (1%, 3%, 5%, 9%, and 20%).

Fig. S20

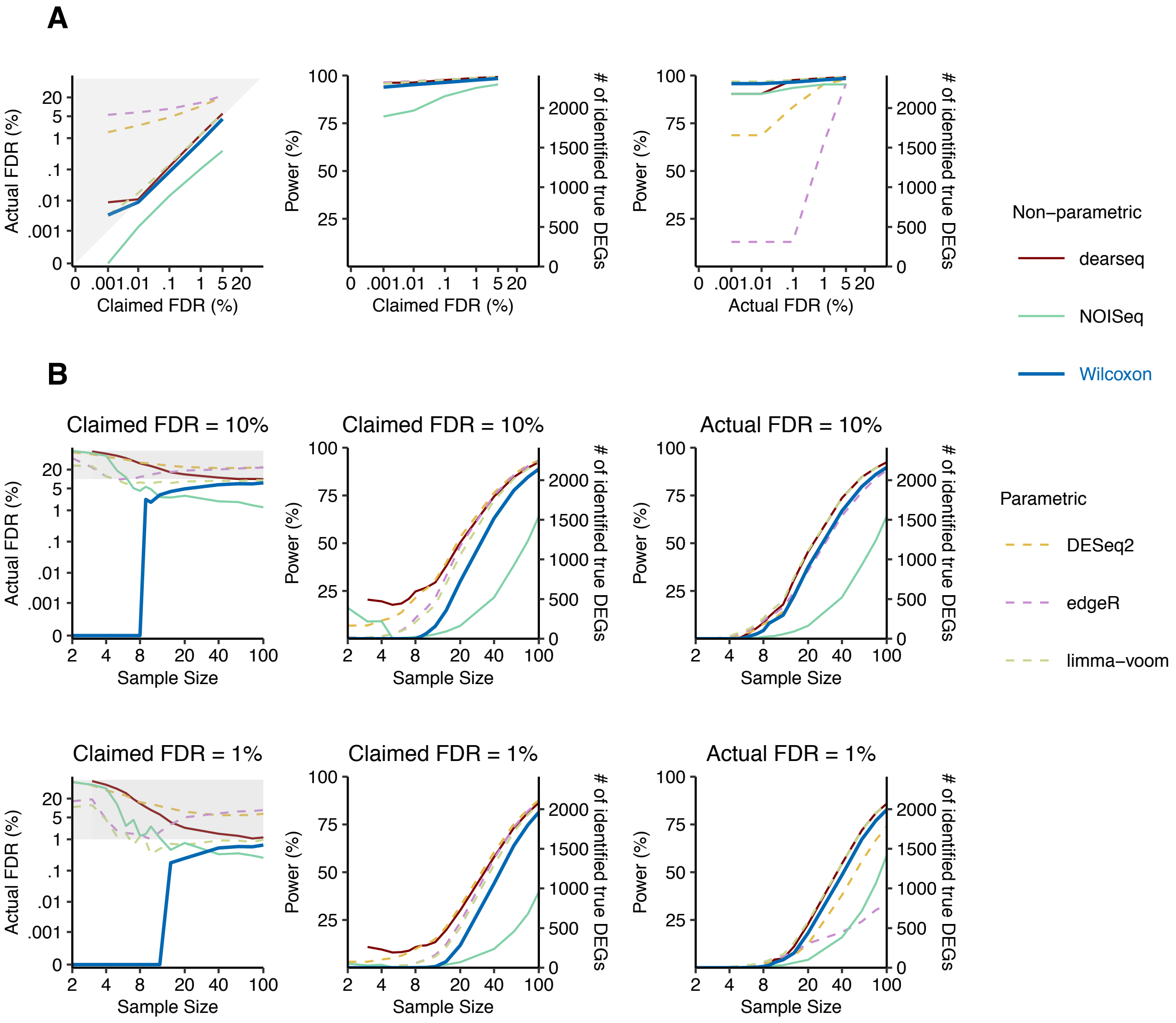

Fig. S20. The Wilcoxon rank-sum test has the best FDR control and power on adipose (subcutaneous vs. visceral) GTEx datasets with semi-synthetic ground truths.

A. The FDR control (left panel), power (middle panel) given the claimed FDRs, and power given the actual FDRs (right panel) under a range of FDR thresholds from 0.001% to 5%.

B. The FDR control (left), power given the claimed FDRs (middle), and power given the actual FDRs (right) for a range of per-condition sample sizes from 2 to 100, under FDR thresholds 10% (top panels) and 1% (bottom panels). The claimed FDRs, actual FDRs, and power were all calculated as the averages of 50 randomly down-sampled datasets.

Fig. S21

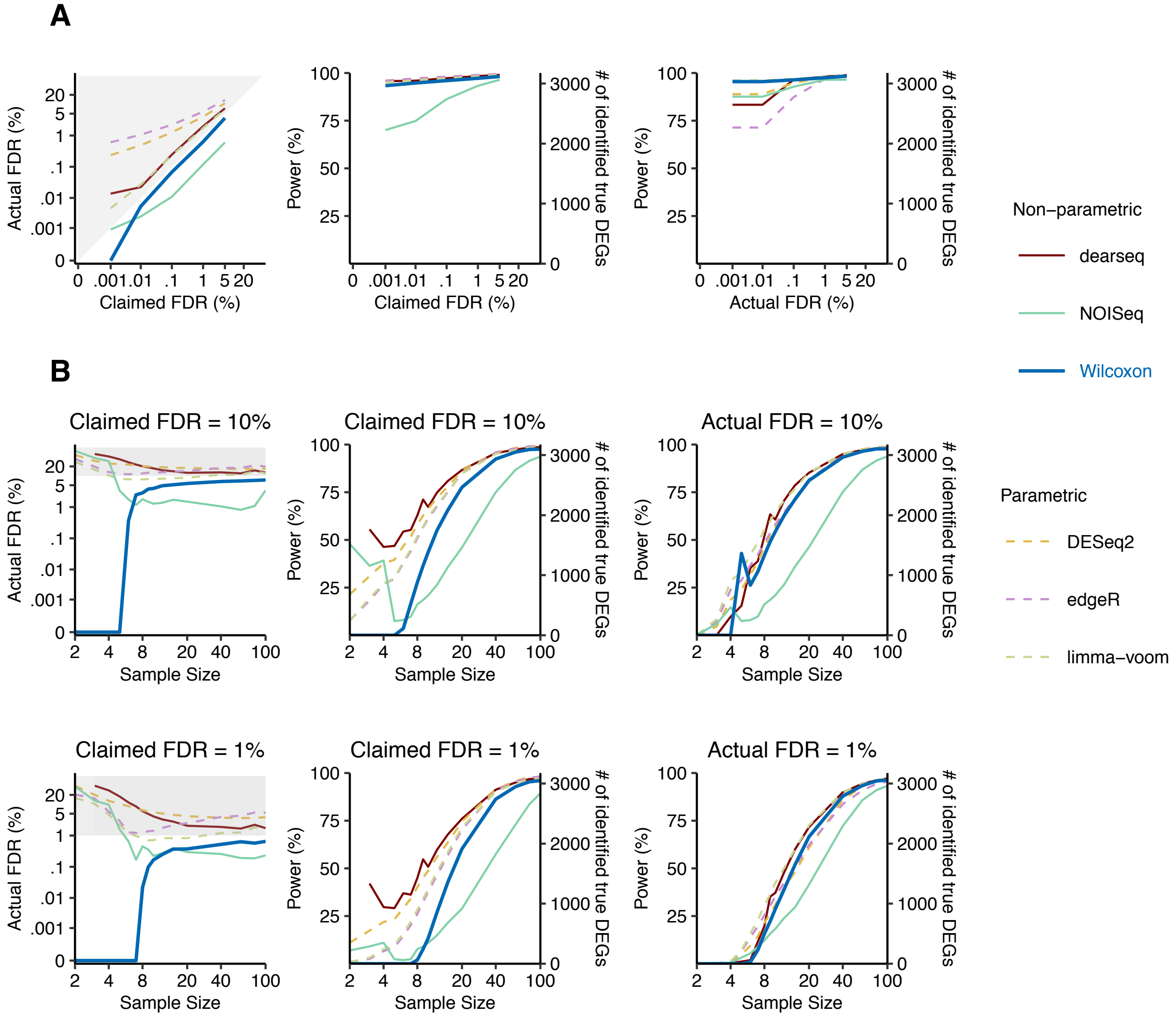

Fig. S21. The Wilcoxon rank-sum test has the best FDR control and power on brain (amygdala vs. spinal cord) GTEx datasets with semi-synthetic ground truths.

A. The FDR control (left panel), power (middle panel) given the claimed FDRs, and power given the actual FDRs (right panel) under a range of FDR thresholds from 0.001% to 5%.

B. The FDR control (left), power given the claimed FDRs (middle), and power given the actual FDRs (right) for a range of per-condition sample sizes from 2 to 100, under FDR thresholds 10% (top panels) and 1% (bottom panels). The claimed FDRs, actual FDRs, and power were all calculated as the averages of 50 randomly down-sampled datasets.

Fig. S22

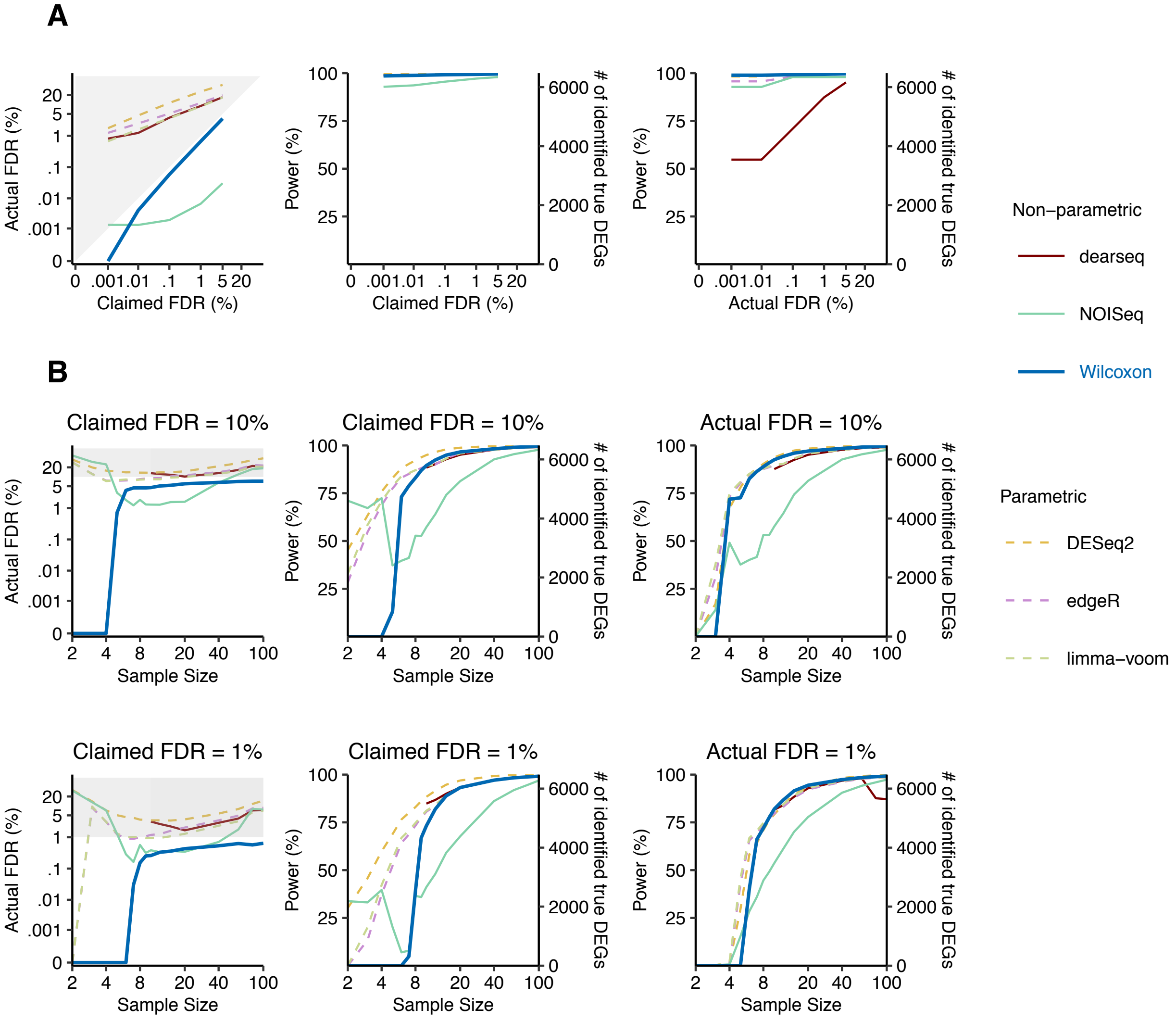

Fig. S22. The Wilcoxon rank-sum test has the best FDR control and power on EVB transformed lymphocytes vs. minor salivary gland GTEx datasets with semi-synthetic ground truths.

A. The FDR control (left panel), power (middle panel) given the claimed FDRs, and power given the actual FDRs (right panel) under a range of FDR thresholds from 0.001% to 5%.

B. The FDR control (left), power given the claimed FDRs (middle), and power given the actual FDRs (right) for a range of per-condition sample sizes from 2 to 100, under FDR thresholds 10%

(top panels) and 1% (bottom panels). The claimed FDRs, actual FDRs, and power were all calculated as the averages of 50 randomly down-sampled datasets.

Fig. S23

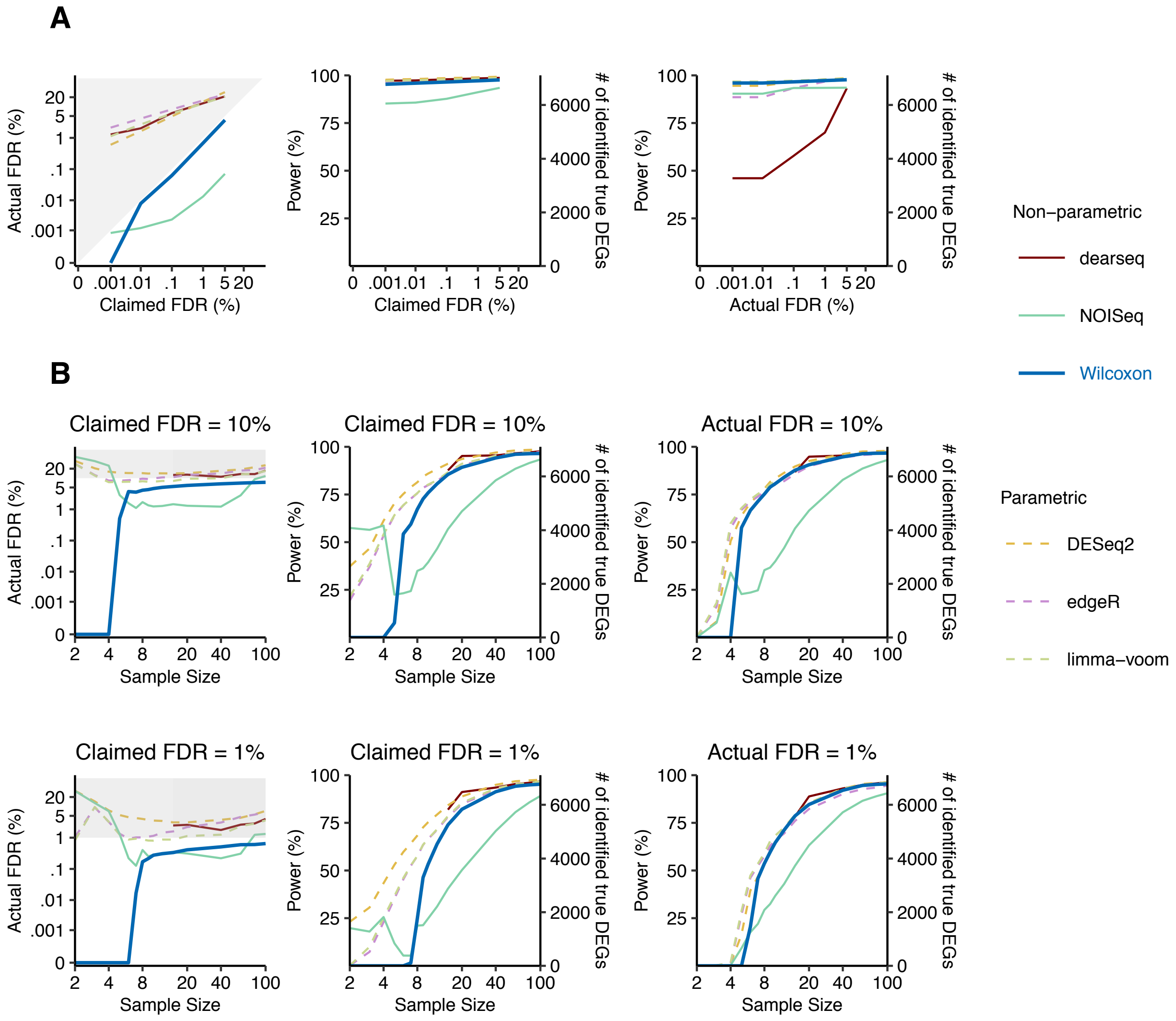

Fig. S23. The Wilcoxon rank-sum test has the best FDR control and power on prostate vs. brain cortex GTEx datasets with semi-synthetic ground truths.

A. The FDR control (left panel), power (middle panel) given the claimed FDRs, and power given the actual FDRs (right panel) under a range of FDR thresholds from 0.001% to 5%.

B. The FDR control (left), power given the claimed FDRs (middle), and power given the actual FDRs (right) for a range of per-condition sample sizes from 2 to 100, under FDR thresholds 10% (top panels) and 1% (bottom panels). The claimed FDRs, actual FDRs, and power were all calculated as the averages of 50 randomly down-sampled datasets.

Fig. S24

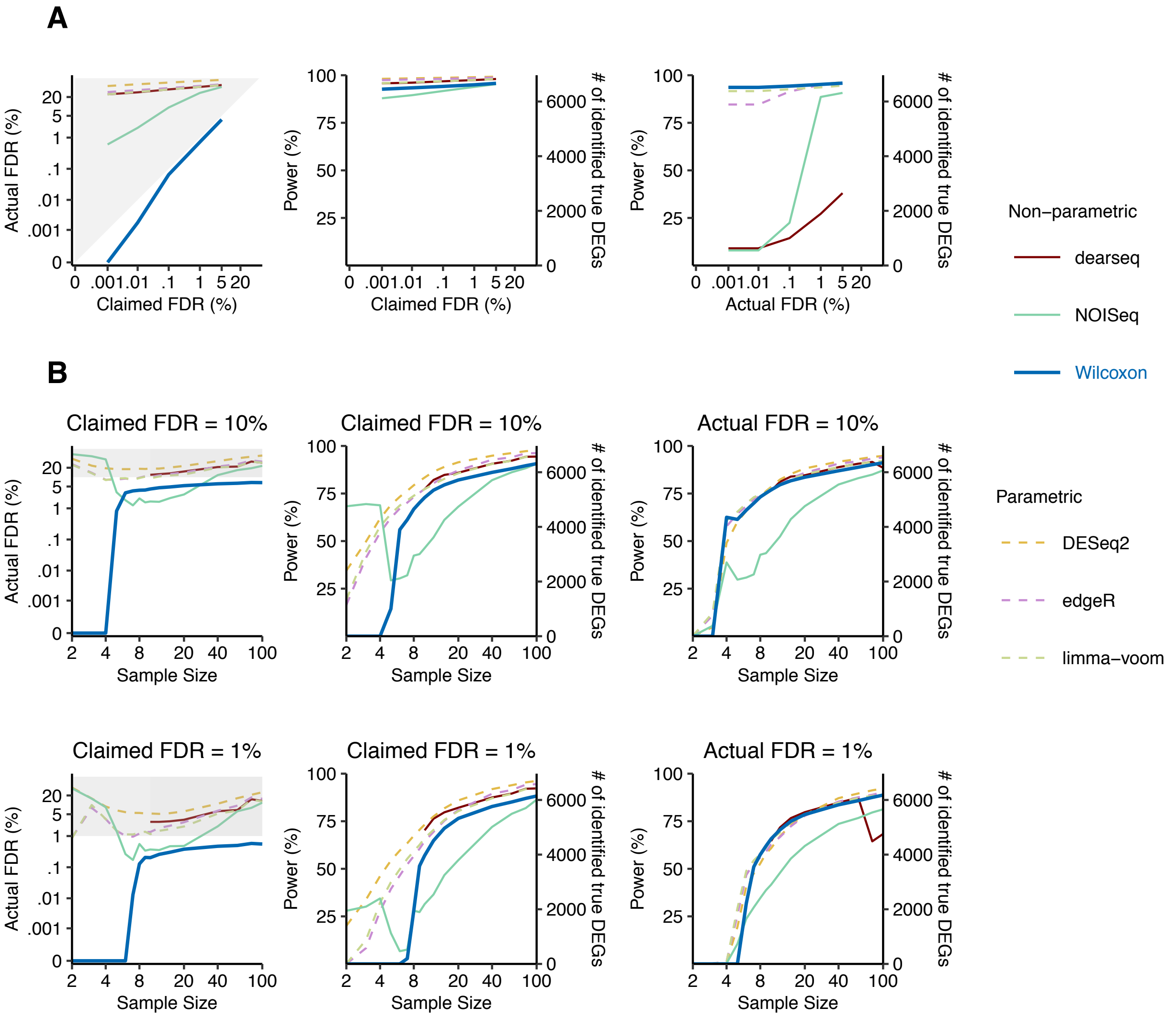

Fig. S24. The Wilcoxon rank-sum test has the best FDR control and power on whole blood vs. muscle GTEx datasets with semi-synthetic ground truths.

A. The FDR control (left panel), power (middle panel) given the claimed FDRs, and power given the actual FDRs (right panel) under a range of FDR thresholds from 0.001% to 5%.

B. The FDR control (left), power given the claimed FDRs (middle), and power given the actual FDRs (right) for a range of per-condition sample sizes from 2 to 100, under FDR thresholds 10% (top panels) and 1% (bottom panels). The claimed FDRs, actual FDRs, and power were all calculated as the averages of 50 randomly down-sampled datasets.

Fig. S25

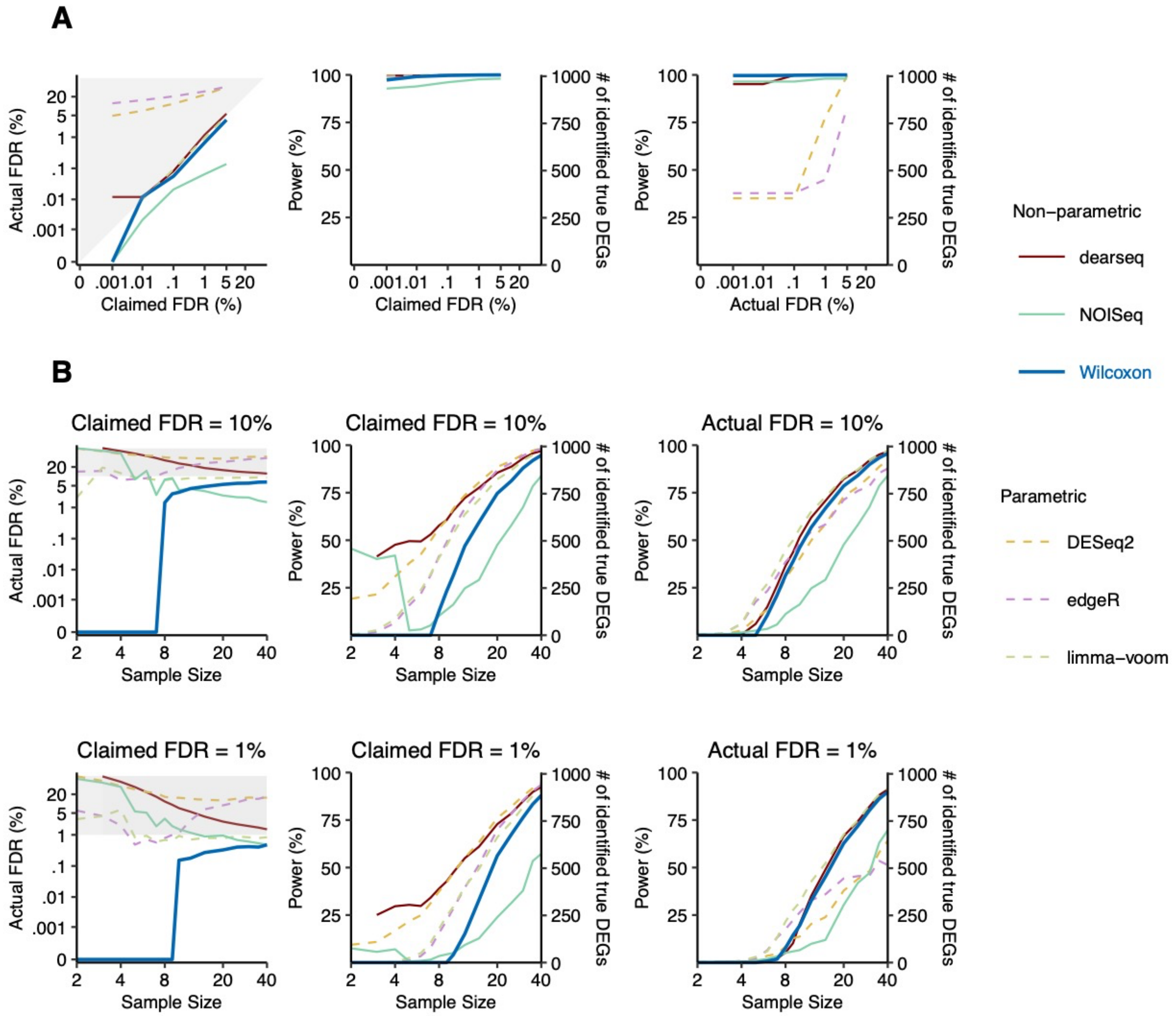

Fig. S25. The Wilcoxon rank-sum test has the best FDR control and power on BRCA TCGA datasets with semi-synthetic ground truths.

A. The FDR control (left panel), power (middle panel) given the claimed FDRs, and power given the actual FDRs (right panel) under a range of FDR thresholds from 0.001% to 5%.

B. The FDR control (left), power given the claimed FDRs (middle), and power given the actual FDRs (right) for a range of per-condition sample sizes from 2 to 100, under FDR thresholds 10% (top panels) and 1% (bottom panels). The claimed FDRs, actual FDRs, and power were all calculated as the averages of 50 randomly down-sampled datasets.

Fig. S26

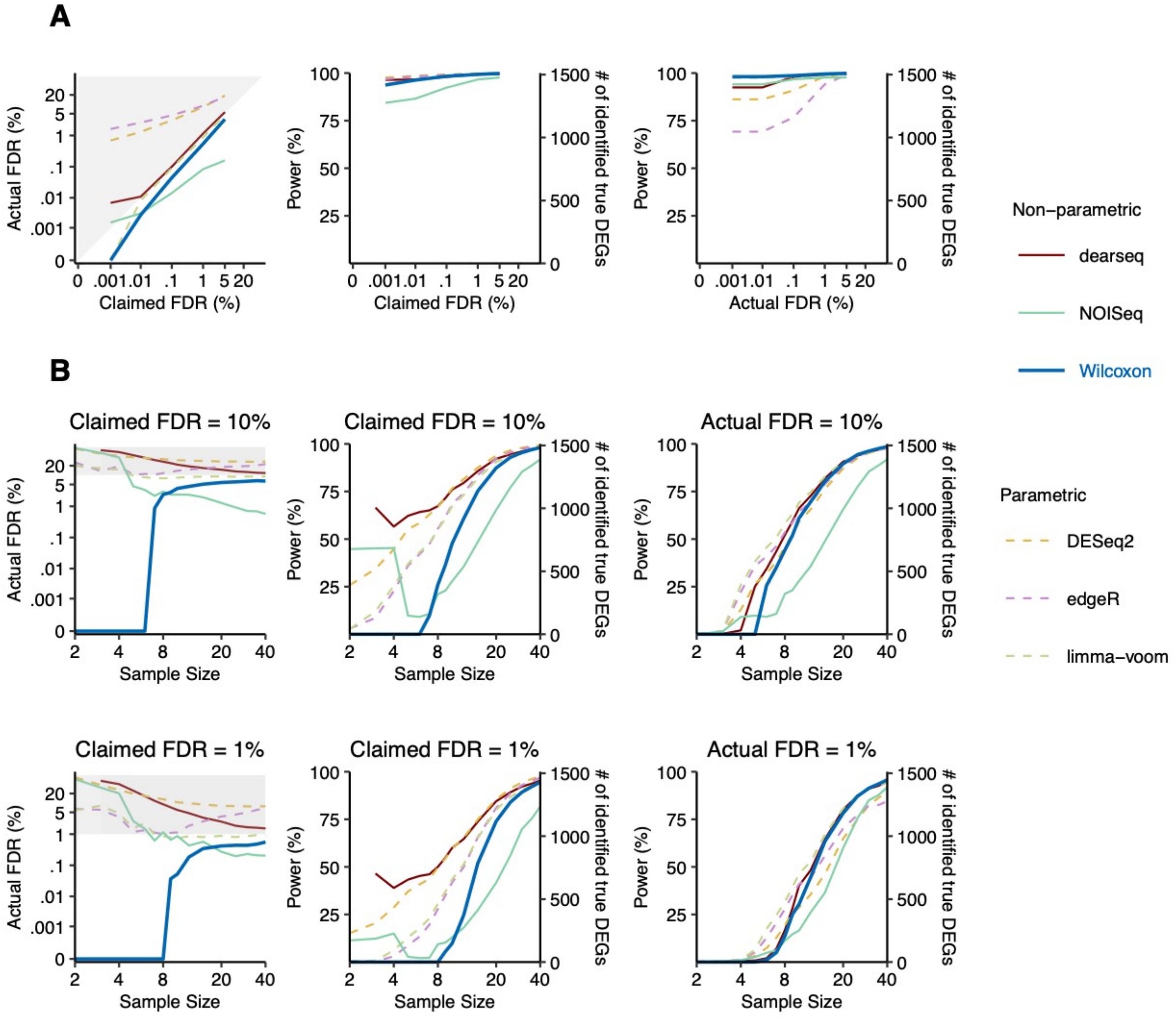

Fig. S26. The Wilcoxon rank-sum test has the best FDR control and power on KIRC TCGA datasets with semi-synthetic ground truths.

A. The FDR control (left panel), power (middle panel) given the claimed FDRs, and power given the actual FDRs (right panel) under a range of FDR thresholds from 0.001% to 5%.

B. The FDR control (left), power given the claimed FDRs (middle), and power given the actual FDRs (right) for a range of per-condition sample sizes from 2 to 100, under FDR thresholds 10% (top panels) and 1% (bottom panels). The claimed FDRs, actual FDRs, and power were all calculated as the averages of 50 randomly down-sampled datasets.

Fig. S27

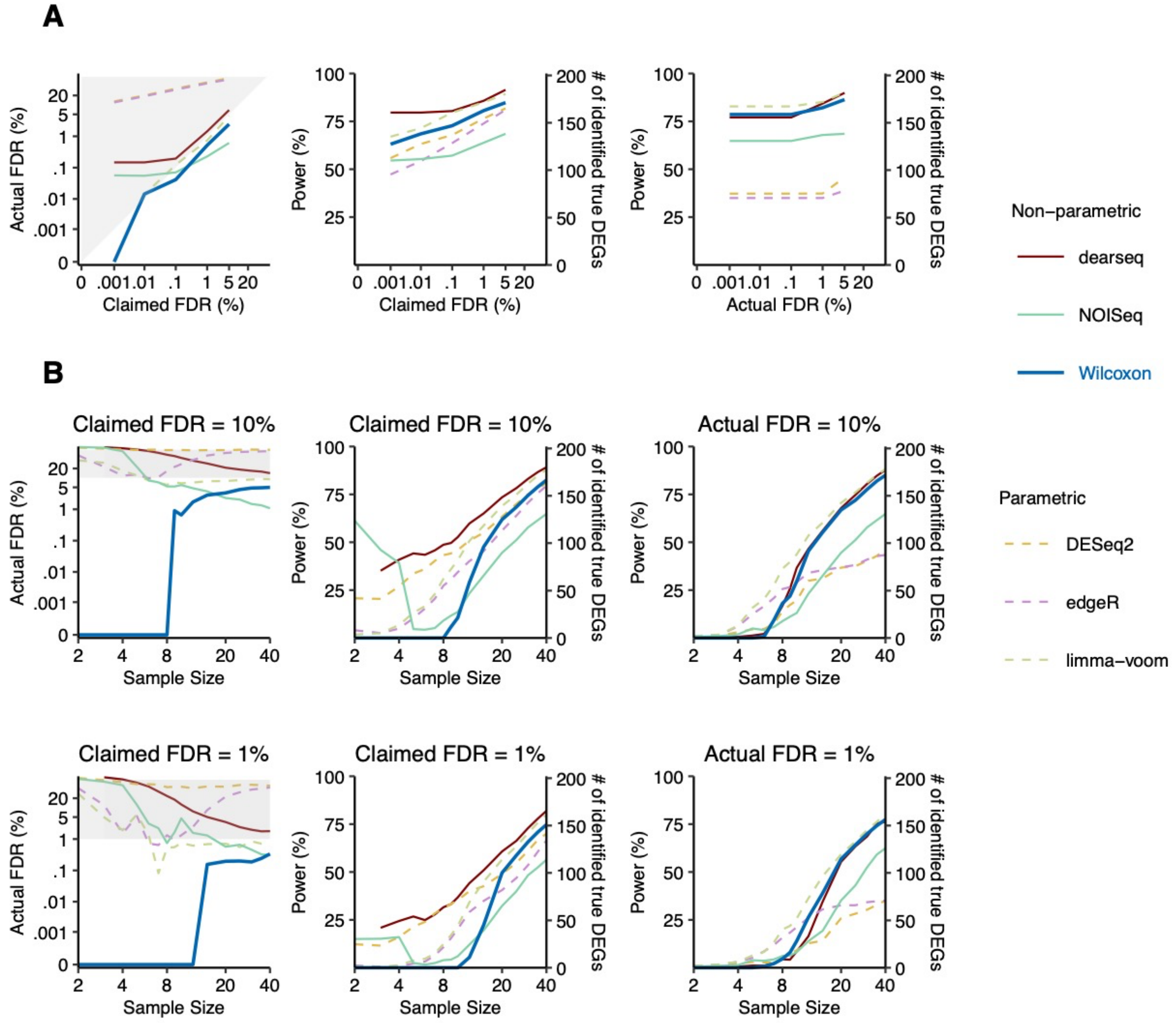

Fig. S27. The Wilcoxon rank-sum test has the best FDR control and power on LIHC TCGA datasets with semi-synthetic ground truths.

A. The FDR control (left panel), power (middle panel) given the claimed FDRs, and power given the actual FDRs (right panel) under a range of FDR thresholds from 0.001% to 5%.

B. The FDR control (left), power given the claimed FDRs (middle), and power given the actual FDRs (right) for a range of per-condition sample sizes from 2 to 100, under FDR thresholds 10% (top panels) and 1% (bottom panels). The claimed FDRs, actual FDRs, and power were all calculated as the averages of 50 randomly down-sampled datasets.

Fig. S28

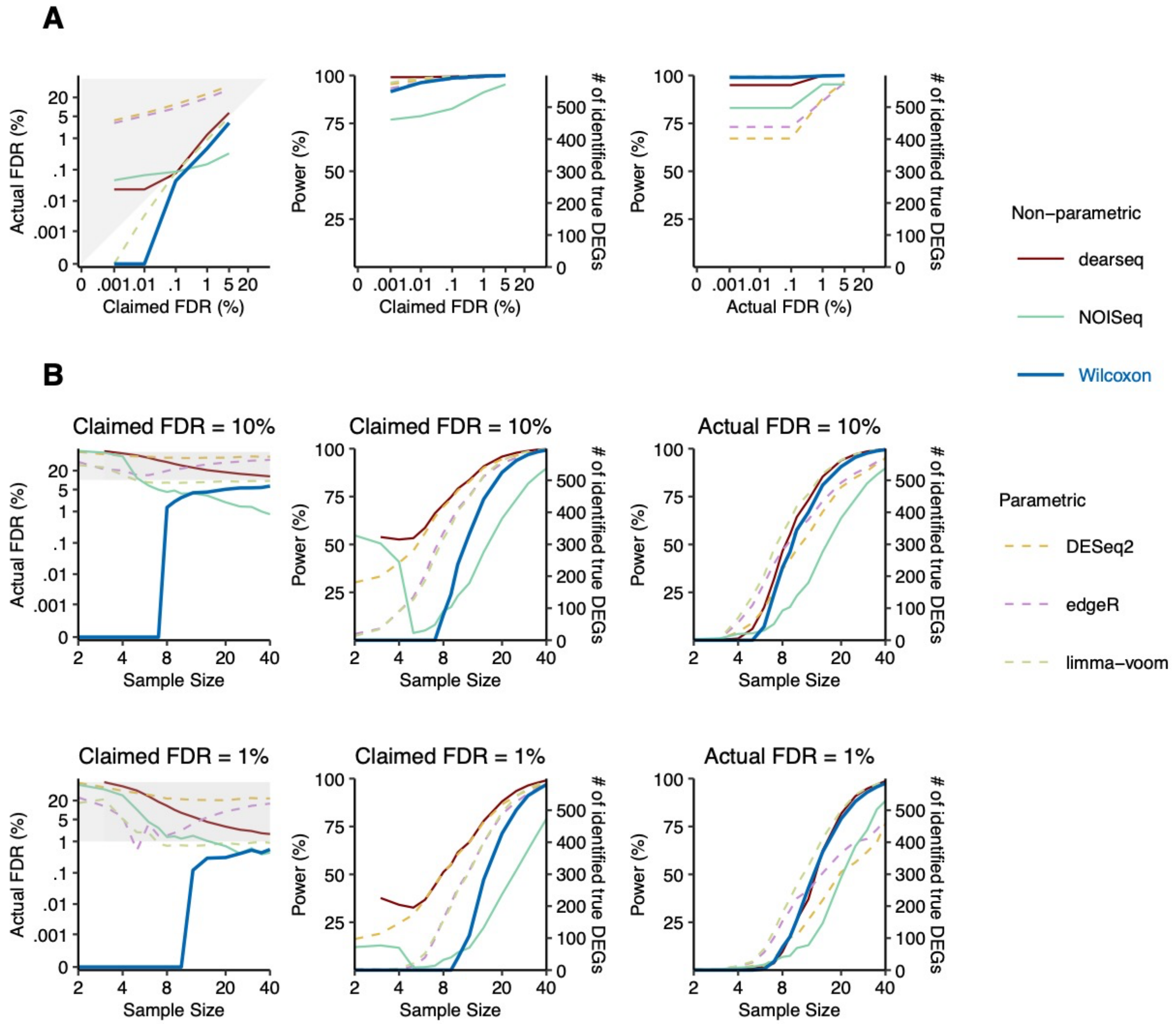

Fig. S28. The Wilcoxon rank-sum test has the best FDR control and power on LUAD TCGA datasets with semi-synthetic ground truths.

A. The FDR control (left panel), power (middle panel) given the claimed FDRs, and power given the actual FDRs (right panel) under a range of FDR thresholds from 0.001% to 5%.

B. The FDR control (left), power given the claimed FDRs (middle), and power given the actual FDRs (right) for a range of per-condition sample sizes from 2 to 100, under FDR thresholds 10% (top panels) and 1% (bottom panels). The claimed FDRs, actual FDRs, and power were all calculated as the averages of 50 randomly down-sampled datasets.

Fig. S29

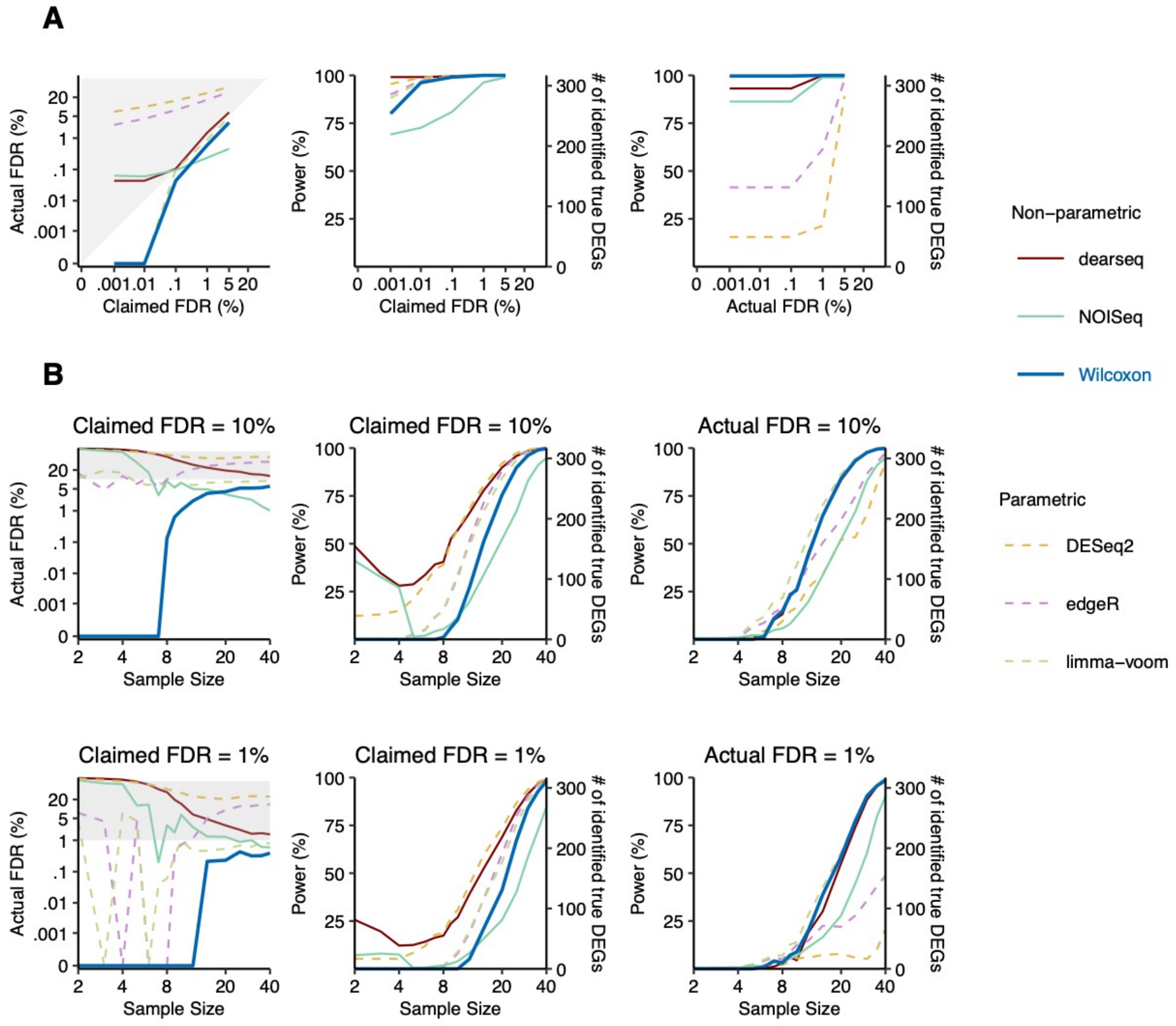

Fig. S29. The Wilcoxon rank-sum test has the best FDR control and power on PRAD TCGA datasets with semi-synthetic ground truths.

A. The FDR control (left panel), power (middle panel) given the claimed FDRs, and power given the actual FDRs (right panel) under a range of FDR thresholds from 0.001% to 5%.

B. The FDR control (left), power given the claimed FDRs (middle), and power given the actual FDRs (right) for a range of per-condition sample sizes from 2 to 100, under FDR thresholds 10% (top panels) and 1% (bottom panels). The claimed FDRs, actual FDRs, and power were all calculated as the averages of 50 randomly down-sampled datasets.

Fig. S30

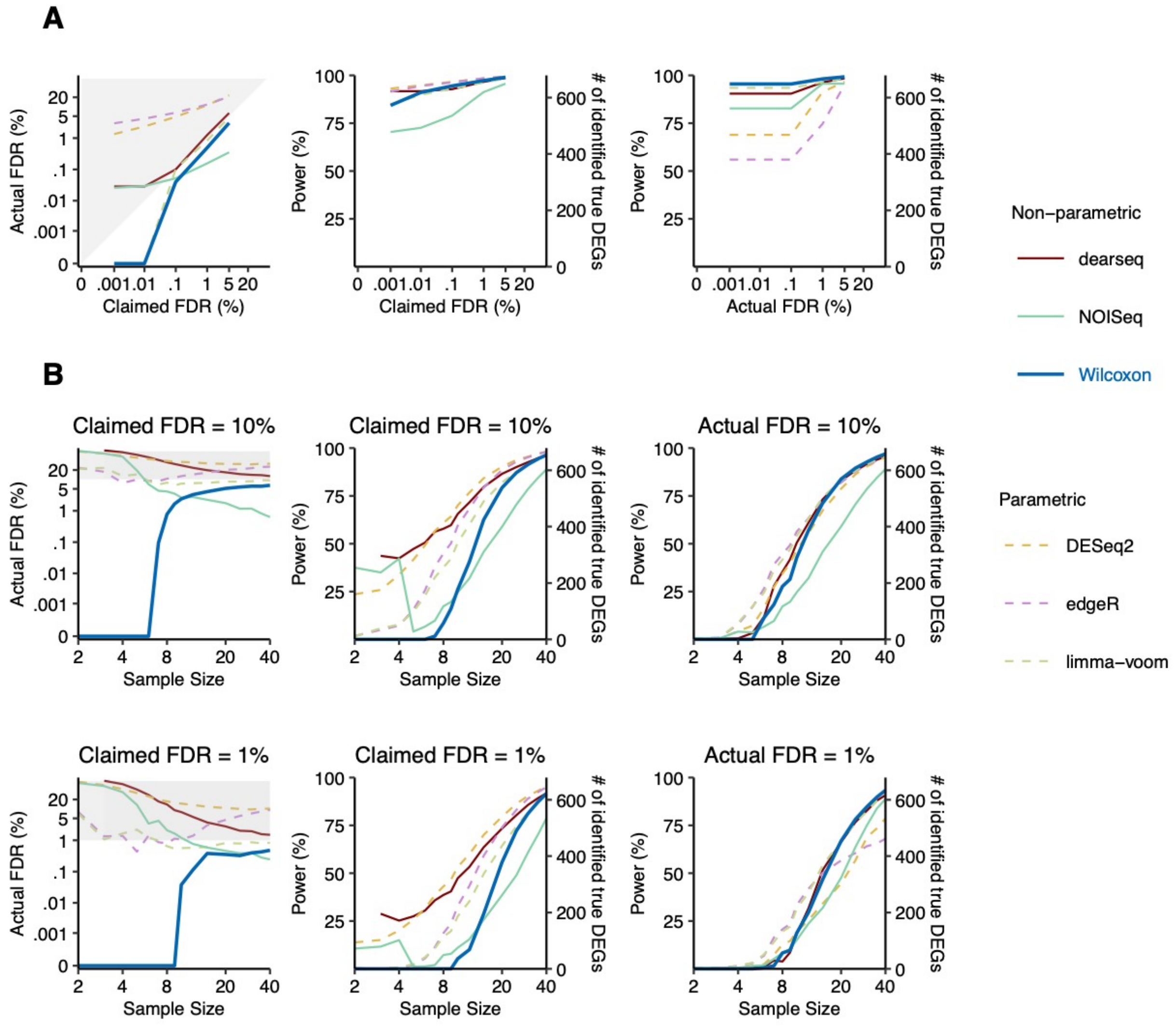

Fig. S30. The Wilcoxon rank-sum test has the best FDR control and power on THCA TCGA datasets with semi-synthetic ground truths.

A. The FDR control (left panel), power (middle panel) given the claimed FDRs, and power given the actual FDRs (right panel) under a range of FDR thresholds from 0.001% to 5%.

B. The FDR control (left), power given the claimed FDRs (middle), and power given the actual FDRs (right) for a range of per-condition sample sizes from 2 to 100, under FDR thresholds 10% (top panels) and 1% (bottom panels). The claimed FDRs, actual FDRs, and power were all calculated as the averages of 50 randomly down-sampled datasets.

Table S1. Summary of information for samples used in this study

Data source	Condition 1	Condition 2	Sample size (Condition 1 vs. Condition 2)	Accession
**Immunotherapy study**	Pre-therapy	On-therapy	51 vs. 58	GSE91061
**TCGA**	BRCA normal tissue[1]	BRCA tumor tissue	112 vs. 112	GDC Xena Hub
	KIRC normal tissue[2]	KIRC tumor tissue	72 vs. 72	
	THCA normal tissue[3]	THCA tumor tissue	58 vs. 58	
	LUAD normal tissue[4]	LUAD tumor tissue	57 vs. 57	
	PRAD normal tissue[5]	PRAD tumor tissue	52 vs. 52	
	LIHC normal tissue[6]	LIHC tumor tissue	50 vs. 50	
**GTEx**	Whole blood	Muscle - skeletal	670 vs. 706	GTEx Portal
	Adipose - subcutaneous	Adipose - visceral	581 vs. 469	
	Heart - atrial appendage	Heart - Left ventricle	372 vs. 386	
	Prostate	Brain - cortex	221 vs. 205	
	Cells - EVB transformed lymphocytes	Minor salivary gland	147 vs. 144	
	Brain - amygdala	Brain - spinal cord	129 vs. 126	

[1]BRCA: Breast invasive carcinoma

[2]KIRC: Kidney renal clear cell carcinoma

[3]THCA: Thyroid carcinoma

[4]LUAD: Lung adenocarcinoma

[5]PRAD: Prostate adenocarcinoma

[6]LIHC: Liver hepatocellular carcinoma
