# Supplementary material for: Exaggerated false positives by popular differential expression methods when analyzing human population samples
Source: Genome Biol. 2022 Mar 15;23:79. doi: 10.1186/s13059-022-02648-4 (PMC8922736; doi:10.1186/s13059-022-02648-4)
Supplement: Supplementary file 3 — Additional file 3. Review history. [file 13059_2022_2648_MOESM3_ESM.docx]

Review History

**Review History**

**First round of review**

**Reviewer 1**

**Were you able to assess all statistics in the manuscript, including the appropriateness of statistical tests used?**

Yes

**Comments to author:**

This paper investigates the false positive rates of popular RNAseq data sets, finding that these methods can have highly inflated FDR in their results. The paper is very well-written, a bit like a detective story, and the investigation is well-executed and convincing. A clear and simple recommendation is given to work with the Wilcoxon test instead.

The findings should be shocking to the users of the methods investigated, and I hope they are quickly picked up by the community.

I have only a few minor comments:

1. An alternative to the Wilcoxon test might be that the authors of edgeR and other methods provide a permutation version of their method. Based on the results presented by the authors, this version would most likely not outperform the Wilcoxon test, but it could be added as a recommendation.

2. Correcting Wilcoxon tests for covariates can be done using probabilistic index models [e.g. 1], see also the PIM package.

3. A mechanism explaining why the largest observed fold change may be a likely false positive is given in [2].

[1] De Neve, J., Thas, O., Ottoy, J. P., & Clement, L. (2013). An extension of the Wilcoxon-Mann-Whitney test for analyzing RT-qPCR data. Statistical Applications in Genetics and Molecular Biology, 12(3), 333-346.

[2] Ebrahimpoor, M., & Goeman, J. J. (2021). Inflated false discovery rate due to volcano plots: problem and solutions. Briefings in Bioinformatics.

**Reviewer 2**

**Were you able to assess all statistics in the manuscript, including the appropriateness of statistical tests used?**

**Yes**

**Comments to author:**

In this manuscript, the authors discussed the anticonservative behavior of the state of the art methods for differential expression analysis in RNASeq data. They evaluated the existing modern methods for RNASeq data analysis and compared them with classical nonparametric tests such as Wilcoxon rank-sum test. They used perturbed data sets as well as semi-synthetic simulated data sets to explore the power and FDR control of these methods. The Wilcoxon test was shown to be performing better in terms of both FDR control and power (given FDR) when the sample size is large.

Overall, the manuscript has its merits, but there are some concerns/suggestions. The concerns and the suggestions are given below in order of their importance.

In the abstract, the authors say: “We report a surprising phenomenon about identifying differentially expressed genes (DEGs) from population-level RNA-seq data: two popular bioinformatics methods, DESeq2 and edgeR, have unexpectedly high false discovery rates (FDRs).” I find their surprise somewhat surprising since thie anticonservative behavior of these methods has been reported earlier in the literature by several papers including the dearseq paper itself, Rocke et al (bioRxiv: https://www.biorxiv.org/content/10.1101/020784v1), Hawinkel et al (Briefings in Bioinformatics, 2017, although this is for microbiome data) etc. A bit more discussion on the existing evaluations of these methods should be included.

Figure 1A and 1C: I think it is better to use relative frequency in the vertical axis to give a better sense of the proportion of false positives.

It is not clear why the authors created 668 permuted data sets for Figure 1. Why this number?

Figure 2B, first column: I think the vertical axis should use a linear scale, which will help better understand exactly how anticonservative the different methods are.

While recommending the Wilcoxon rank-sum test, the authors should discuss that the Wilcoxon rank-sum test essentially tests a different null (and alternative) hypothesis compared to some of the methods. It may be okay to use it, but it is worth noting that not all the tests discussed have the same null and they may be sensitive to the different alternative hypotheses.

At the very end, the authors recommend Kruskal-Wallis test for more than two groups. Do the authors believe or have any evidence that similar to Wilcoxon test, the Kruskal-Wallis will also outperform the newer methods? Or do they consider this beyond the scope of this paper? They should clearly mention this.

I think they could have included in their evaluation some other common classical tests such as two-sample t-test or linear regression, possibly after transforming the variables. Although the normality assumption will not be satisfied for RNASeq data in small samples, these methods can potentially perform at least at the same level of the Wilcoxon test when the sample size is large, which is the focus of this paper. Including results from linear regression may be especially interesting since it is not limited to the 2-group comparison and can handle covariates. Including this additional method in the paper should not significantly increase the length of the paper.

Page 9, line 4: “however, for large-sample-size data, the Wilcoxon rank-sum test is the best choice for its solid FDR control and good power.” – Please discuss how large. Figure 2B indicates that some other methods perform better for moderately large sample sizes, and even for n=100. In particular, based on Figure 2B, dearseq does not seem to be too bad in terms of FDR control, and has power as good as Wilcoxon. There should be a clearer suggestion for the practitioner on at what point Wilcoxonn test should be used. The suggestion that talks about n=8 does not seem to be fair to dearseq based on Figure 2B.

“Large Sample Crisis?” in the title makes it catchy, but I think it can also be a somewhat confusing since these findings indicate the non-robustness of the modern differential analysis tests rather than a problem with large samples. If the model assumptions are not met, they are likely to perform poorly in small samples too, although, as discussed, they still could be a better option than the Wilcoxon test for small samples. I recommend changing this part of the title.

Page 5, line 56: “Surprisingly, DESeq2 and edgeR identified more DEGs from 84.88% and 78.89% of these permuted datasets than from the original dataset” – This sentence is confusing. Please rephrase.

**Reviewer 3**

**Were you able to assess all statistics in the manuscript, including the appropriateness of statistical tests used?**

**Yes**

**Comments to author:**

This manuscript addresses an important question on the performance of software for identifying DE genes using RNA-seq by comparing 6 methods on semi-synthetic datasets. In particular, the authors found that (1) popular model-based packages, such as DEseq2 and edgeR, severely underestimate FDRs on the permuted datasets, (2) there is a large deviation between DEGs identified by different methods, notably DEseq2 and edgeR, and (3) Wilcoxon rank sum test, a general-purpose non-parametric test, outperforms all DEG software when sample size is large (>8). These findings will provide useful guidance for practitioners on choosing DEG software for RNA-seq analyses. Some of the findings resonate with this reviewer's own observation with the software DEseq2 and edgeR, so it is nice to a systematic analysis on this aspect.

However, comparison of RNA-seq DEG software is not new and some of the findings discussed in this manuscript have been found before. For example, a google search shows that at least [1-2] (maybe more) have done some systematic comparisons between DEG software for RNA-seq analyses. A very well-cited paper [2] (400+ citation) noticed that non-parametric method (Samseq) outperforms DEseq, edgeR and limma (their Figure 2) when sample size is relatively large. Similar observations have been discussed in some RNA-seq analysis forum [3].

1. Yet, none of the previous comparisons were mentioned in this manuscript. A comparison with previous work is needed. In particular, it should be pointed out what is new in the current comparison and the gain of knowledge over the existing literature on the comparison of DEG methods. Are the findings different from the existing comparisons? What are the differences and why?

2. Based on this reviewer's own experience, the performance of different software depends on the proportion of DEG's. This is also discussed in [1]. It will be useful to see how the performance changes with the proportion of DEG's in this work too. Since this work is based on semi-synthetic data, it is easy to do systematic analyses that have different proportions of DEG.

3. In this work, the author attributed the inflated FDR in DEseq2 and edgeR to lack of fit of the negative binomial model. While this can be a possible reason, is this the only reason? If so, why the FDR is better calibrated for these methods when the sample size is larger? Can the authors look into the genes that are falsely identified in more details?

4. Why were the spurious DEGs enriched in immune-related gene ontology (GO) terms in the permuted immunothreapy data datasets? Any possibility that the labels were not mixed well enough?

5. Though Wilcoxon performs well when sample size is >8, it is lack of power when sample size is below 8. So the claim in the abstract ("Based on these results, for population-level RNA-seq studies, we recommend the Wilcoxon rank-sum test.") seems to be too broad. What is the recommendation for sample size <8?

6. Though several sets of datasets were used for demonstration, it is unclear what are the differences between the characteristics of these datasets. What is the rationale to choose them? Can they represent a good coverage of different situations that practitioners would encounter in practice?

[1] Corchete, L.A., Rojas, E.A., Alonso-López, D. et al. Systematic comparison and assessment of RNA-seq procedures for gene expression quantitative analysis. Sci Rep 10, 19737 (2020). https://doi.org/10.1038/s41598-020-76881-x

[2] Fatemeh Seyednasrollah, Asta Laiho, Laura L. Elo, Comparison of software packages for detecting differential expression in RNA-seq studies, Briefings in Bioinformatics, Volume 16, Issue 1, January 2015, Pages 59-70, https://doi.org/10.1093/bib/bbt086

[3] SEQanswers: the next generation sequencing community http://seqanswers.com/forums/showthread.php?t=20242

**Authors Response**

**Point-by-point responses to the reviewers’ comments:**

Responses to Reviewer #1

This paper investigates the false positive rates of popular RNAseq data sets, finding that these

methods can have highly inflated FDR in their results. The paper is very well-written, a bit like a

detective story, and the investigation is well-executed and convincing. A clear and simple

recommendation is given to work with the Wilcoxon test instead.

The findings should be shocking to the users of the methods investigated, and I hope they are

quickly picked up by the community.

*Response: We thank the reviewer for acknowledging the significance of our findings and for*

*praising our writing.*

I have only a few minor comments:

1. An alternative to the Wilcoxon test might be that the authors of edgeR and other methods

provide a permutation version of their method. Based on the results presented by the authors, this

version would most likely not outperform the Wilcoxon test, but it could be added as a

recommendation.

*Response: We thank the reviewer for this suggestion. However, we have checked the methods*

*other than the Wilcoxon rank-sum test and found that none of them has implemented a*

*permutation version. Although we could in theory implement a permutation test on our own*

*based on each method's test statistic, this will be too computationally intensive because obtaining*

*p-values at the 0.001 resolution would require a minimum of 1000 permutations. Since multiple*

*testing correction for thousands of genes requires high-resolution p-values (and the 0.001*

*resolution is often insufficient), we do not think that implementing a permutation version of*

*edgeR and other methods would offer practical solutions to users. Hence, we did not make this*

*recommendation.*

2. Correcting Wilcoxon tests for covariates can be done using probabilistic index models [e.g. 1],

see also the PIM package.

[1] De Neve, J., Thas, O., Ottoy, J. P., & Clement, L. (2013). An extension of the WilcoxonMann-Whitney test for analyzing RT-qPCR data. Statistical Applications in Genetics and

Molecular Biology, 12(3), 333-346

*Response: We thank the reviewer for this suggestion. We have added the following sentence to*

*the last paragraph of our manuscript.*

*"Hence, to use the Wilcoxon rank-sum test for DEG identification, researchers can normalize*

*RNA-seq samples to remove batch effects or use the probabilistic index models to adjust the*

*Wilcoxon rank-sum test for covariates."*

3. A mechanism explaining why the largest observed fold change may be a likely false positive

is given in [2].

[2] Ebrahimpoor, M., & Goeman, J. J. (2021). Inflated false discovery rate due to volcano plots:

problem and solutions. Briefings in Bioinformatics.

*Response: We thank the reviewer for suggesting this paper, whose authors discussed the false*

*positives resulted from a “volcano plot selection” procedure: discovering DE genes as those with*

*both small adjusted p-values and large estimated effect sizes in a volcano plot. The authors used*

*simulation experiments and RNA-seq data to illustrate that this procedure could result in*

*substantially inflated type I error rates. We have added the following sentence to our revised*

*manuscript to discuss this issue.*

*"This finding is consistent with a recent paper, which also reported that selecting the genes with*

*the largest estimated differences between the two conditions would inflate the FDR."*

Responses to Reviewer #2

In this manuscript, the authors discussed the anticonservative behavior of the state of the art

methods for differential expression analysis in RNASeq data. They evaluated the existing

modern methods for RNASeq data analysis and compared them with classical nonparametric

tests such as Wilcoxon rank-sum test. They used perturbed data sets as well as semi-synthetic

simulated data sets to explore the power and FDR control of these methods. The Wilcoxon test

was shown to be performing better in terms of both FDR control and power (given FDR) when

the sample size is large.

Overall, the manuscript has its merits, but there are some concerns/suggestions. The concerns

and the suggestions are given below in order of their importance.

*Response: We thank the reviewer for summarizing our main findings and acknowledging the*

*merits of our study.*

1. In the abstract, the authors say: "We report a surprising phenomenon about identifying

differentially expressed genes (DEGs) from population-level RNA-seq data: two popular

bioinformatics methods, DESeq2 and edgeR, have unexpectedly high false discovery rates

(FDRs)." I find their surprise somewhat surprising since the anticonservative behavior of these

methods has been reported earlier in the literature by several papers including the dearseq paper

itself, Rocke et al (bioRxiv: https://www.biorxiv.org/content/10.1101/020784v1), Hawinkel et al

(Briefings in Bioinformatics, 2017, although this is for microbiome data) etc. A bit more

discussion on the existing evaluations of these methods should be included.

*Response: We are glad that the reviewer is aware of the inflated FDR issue of DESeq2 and*

*edgeR; however, we are afraid that many biologists who analyze RNA-seq data may not know*

*this issue, as evidenced by Reviewer 1’s comment that*

*“The findings should be shocking to the users of the methods investigated, and I hope they are*

*quickly picked up by the community.”*

*We acknowledge that several papers including the dearest paper have criticized the inflated FDR*

*issue of DESeq2 and edgeR, but this issue has not been paid enough attention in the field (as we*

*wrote in our manuscript that consortia like GTEx and TCGA still used DESeq2 and edgeR for*

*large-sample DE analysis). A possible reason is that many readers are not easily convinced by*

*the criticism of existing methods in a paper that proposes a new method (e.g., the dearseq*

*method); another possible reason is that biologists are hardly convinced by model-based*

*simulation results that revealed the inflated FDRs of DESeq2 and edgeR (as in the existing*

*benchmark papers and the dearseq paper), because they do not know if the models used for*

*simulation mimic the real biology.*

*Hence, our paper is unique in that it solely uses important real datasets (including the*

*immunotherapy dataset as the opening case) to reveal the FDR inflation issue as a concrete crisis,*

*without relying on any additional model assumptions. Please note that our use of semi-synthetic*

*datasets is only for power evaluation and irrelevant to the FDR calibration.*

*The reason why we could use real datasets for FDR calibration is that our datasets have large*

*sample sizes, allowing us to generate many enough permuted datasets for FDR estimation. In*

*contrast, previous studies focused on small sample sizes, a scenario where FDR cannot be*

*reliably estimated by permutation because the number of possible permutation is too small; that*

*is why they all needed to use model-based simulation for FDR estimation, leaving the doubt if*

*their simulated data actually resembled real data.*

*Moreover, we would like to point out that, although the dearseq paper raised the inflated FDR*

*concern about DESeq2 and edgeR, the dearseq method still has the inflated FDR issue, as we*

*found in our benchmarking results.*

*Following the reviewer's suggestion, we have added a supplementary table to summarize the*

*existing benchmark literature (Supplementary Table 2). We have also added the following*

*paragraph to our revised manuscript to highlight the novelty of our study*

*"Although several studies had evaluated the performance of various DEG identification methods before our studies, they had been restricted to using simulated datasets with small sample sizes (Supplementary Table 2). Unlike all previous studies, our study evaluated the FDRs of DEG identification methods by permuting real RNA-seq datasets, without relying on any model assumptions. The reason why we could use real datasets for FDR evaluation is that our datasets have large sample sizes, allowing us to generate many enough permuted datasets for FDR estimation. In contrast, previous studies had focused on small sample sizes, a scenario where FDR cannot be reliably estimated by permutation because the number of possible permutation is too small; that is why they all had to use model-based simulation for FDR estimation, leaving the doubt if their simulated data actually resembled real data. Another innovation of our study is the recommendation of the classical Wilcoxon rank-sum test, which has been ignored by all existing benchmark studies (Supplementary Table 2). For the first time, we found that the Wilcoxon rank-sum test consistently controlled the FDR and achieved good power for DEG identification from large-sample-size RNA-seq data. Although the recently developed dearseq method had claimed it to have overcome the inflated FDR issue of DESeq2 and edgeR on large-sample-size data, our evaluation shows that dearseq still had the issue and did not outperform the Wilcoxon rank-sum test."*

2. Figure 1A and 1C: I think it is better to use relative frequency in the vertical axis to give a

better sense of the proportion of false positives.

*Response: Since no DEGs are expected to be identified from permuted datasets, all the DEGs*

*identified from a sufficiently permuted dataset (please see our next response) should be false*

*positives. We do not think it is informative to show the proportion (i.e., relative frequency; the*

*number of false positives among the identified DEGs in a dataset) of false positives because the*

*proportion would be 100% for a permuted dataset if any DEGs are identified. That is the reason*

*why we use the numbers of DEGs identified on permuted datasets (all such DEGs are false*

*positives) to compare DE methods, and a good method should have small numbers across*

*permutations.*

3. It is not clear why the authors created 668 permuted data sets for Figure 1. Why this number?

*Response: We apologize for the confusion. We have added the following sentences to the*

*revised manuscript to explain how we generated the sufficiently permuted datasets to ensure that*

*DEGs identified from these datasets are false positives.*

*"We first generated 1000 negative-control datasets by randomly permuting the two-condition*

*labels (pre-nivolumab and on-nivolumab) of the 109 RNA-seq samples in this immunotherapy*

*dataset. To address the issue that some permuted datasets might not have the two-condition*

*labels mixed sufficiently, we then filtered the permuted datasets and only retained 668 datasets*

*whose fractions of samples having original condition labels were between the 1st and 3rd*

*quartiles of the fractions of all 1000 permuted datasets (Methods; in the two extreme cases, the*

*fraction is 1 for the original dataset and 0 for the permuted dataset where the two-condition*

*labels are swapped, and the samples are not mixed at all; so fractions close to 0 or 1 indicate poor*

*mixing of condition labels)"*

4. Figure 2B, first column: I think the vertical axis should use a linear scale, which will help

better understand exactly how anticonservative the different methods are.

*Response: We thank the reviewer for this helpful suggestion. We kept the log-scale plot as the*

*main figure to emphasize the differences among DEG identification methods, so that the plot*

*would not be dominated by the methods having the worst FDRs. Following the reviewer’s*

*suggestion, however, we have generated an alternative figure with a linear-scale y axis to help*

*readers better understand the severe anticonservativeness of some methods (new Supplementary*

*Figure 18; pasted below).*

*Supplementary Fig. 18. The Wilcoxon rank-sum test has the best FDR control and power on heart left ventricle vs. atrial appendage GTEx datasets with semi-synthetic ground truths.*

*A. The FDR control (left panel), power (middle panel) given the claimed FDRs, and power given the actual FDRs (right panel) under a range of FDR thresholds from 0.001% to 5%.*

*B. The FDR control (left), power given the claimed FDRs (middle), and power given the actual FDRs (right) for a range of per-condition sample sizes from 2 to 100, under FDR thresholds 10% (top panels) and 1% (bottom panels). The claimed FDRs, actual FDRs, and power were all calculated as the averages of 50 randomly down-sampled datasets.*

5. While recommending the Wilcoxon rank-sum test, the authors should discuss that the

Wilcoxon rank-sum test essentially tests a different null (and alternative) hypothesis compared to

some of the methods. It may be okay to use it, but it is worth noting that not all the tests

discussed have the same null and they may be sensitive to the different alternative hypotheses.

*Response: We thank the reviewer for this suggestion. We have added the following sentences to*

*our manuscript to discuss the difference in null hypothesis between the Wilcoxon rank-sum test*

*and other methods.*

"*Considering that a likely cause of the model violation is the existence of outliers, we examined all the genes that were mistakenly identified as DEGs in at least ten permuted datasets, and we detected the existence of outliers in all these genes’ measurements relative to the assumed negative binomial model (Supplementary Fig. 5). It is well known that estimating the mean is not informative in the existence of outliers. However, in parametric methods like edgeR and DESeq2, the null hypothesis is that a gene has the same mean under the two conditions. Hence, it is expected that the testing result would be severely affected by the existence of outliers. In contrast, the Wilcoxon rank-sum test is more robust to outliers due to its different null hypothesis: a gene’s measurement under one condition has equal chances of being less or greater than its observation under the other condition. That is, the Wilcoxon rank-sum test concerns more about the ranks than the magnitudes of measurements, making it robust to outliers. Unlike the t test, the Wilcoxon rank-sum test has no requirement on the data distributions. It concerns the null hypothesis that a random observation in one sample has equal chances of being less or greater than a random observation in the other sample, an informative null hypothesis to test against regardless of the skewness of data distributions. Admittedly, the DE genes found by the Wilcoxon rank-sum test may have distribution differences other than the mean difference between the two conditions, but we do not regard this as a notable drawback of the Wilcoxon rank-sum test. The reason is that genes may still be of biological interest if they have the same mean but different spread under the two conditions."*

6. At the very end, the authors recommend Kruskal-Wallis test for more than two groups. Do the

authors believe or have any evidence that similar to Wilcoxon test, the Kruskal-Wallis will also

outperform the newer methods? Or do they consider this beyond the scope of this paper? They

should clearly mention this.

*Responses: We thank the reviewer for pointing out this issue. As we consider the comparison of*

*more than two groups beyond the scope of this paper, we have removed this recommendation*

*from our revised manuscript. We have emphasized that our study only focused on two-group*

*comparisons in the revised manuscript.*

7. I think they could have included in their evaluation some other common classical tests such as

two-sample t-test or linear regression, possibly after transforming the variables. Although the

normality assumption will not be satisfied for RNASeq data in small samples, these methods can

potentially perform at least at the same level of the Wilcoxon test when the sample size is large,

which is the focus of this paper. Including results from linear regression may be especially

interesting since it is not limited to the 2-group comparison and can handle covariates. Including

this additional method in the paper should not significantly increase the length of the paper.

*Responses: We thank the reviewer for this suggestion. We did not include the two-sample t test*

*or the Welch's correction as an alternative to the Wilcoxon rank-sum test for three reasons. First,*

*if a gene's two sets of observations under the two conditions (i.e., two samples) are from nonGaussian distributions (which is the case for RNA-seq data), the t test is only valid when the two*

*sample sizes are large and the central limit theory holds (then the gene's two sample means are*

*approximately Gaussian distributed); however, the central limit theorem only holds when the two*

*samples are from distributions with finite second moments, excluding the possibility that*

*samples may come from heavy-tailed distributions with infinite second moments*

*(https://en.wikipedia.org/wiki/Stable_distribution#A_generalized_central_limit_theorem).*

*Second, the t test is not invariant to non-linear monotone transformations on the observations; for*

*example, the two samples may have the same population mean on the original scale (i.e., the null*

*hypothesis is true) but different population means on the log scale (i.e., the null hypothesis is*

*false); there is no consensus on what transformation is optimal for RNA-seq data. In contrast, the*

*Wilcoxon rank-sum test is invariant to monotone transformations. Third, the t test only concerns*

*the mean difference between the two samples' distributions, but the mean parameter is not*

*informative if a distribution is heavily skewed, and estimating the mean parameter is not robust*

*to the existence of outliers. Unlike the t test, the Wilcoxon rank-sum test has no requirement on*

*the data distributions. It concerns the null hypothesis that a random observation in one sample*

*has equal chances of being less or greater than a random observation in the other sample, an*

*informative null hypothesis to test against regardless of the skewness of data distributions.*

*Admittedly, the DE genes found by the Wilcoxon rank-sum test may have distribution*

*differences other than the mean difference between the two conditions, but we do not regard this*

*as a notable drawback of the Wilcoxon rank-sum test. The reason is that genes may still be of*

*biological interest if they have the same mean but different spread under the two conditions.*

*We have added the above three reasons to the revised manuscript.*

*Since we only focused on two-group comparisons in this study, we have removed all discussion*

*about comparing more than two groups from our revised manuscript.*

8. Page 9, line 4: "however, for large-sample-size data, the Wilcoxon rank-sum test is the best

choice for its solid FDR control and good power." – Please discuss how large. Figure 2B

indicates that some other methods perform better for moderately large sample sizes, and even for

𝑛 = 100. In particular, based on Figure 2B, dearseq does not seem to be too bad in terms of

FDR control, and has power as good as Wilcoxon. There should be a clearer suggestion for the

practitioner on at what point Wilcoxonn test should be used. The suggestion that talks about

𝑛 = 8 does not seem to be fair to dearseq based on Figure 2B.

*Response: We note that Figure 2C is a fairer comparison than Figure 2B because Figure 2C has*

*methods’ actual FDRs in the x axis. Although dearseq did not seem too bad in terms of FDR*

*control, it was still worse than the Wilcoxon rank-sum test (in terms of power) and failed to*

*control the FDR. This phenomenon is clearer with our improved illustration, where failed FDR*

*control falls in the gray area. Based on our comprehensive evaluation results, we recommend*

*practitioners to use the Wilcoxon rank-sum test if their datasets have more than 8 samples per*

*condition.*

9. "Large Sample Crisis?" in the title makes it catchy, but I think it can also be a somewhat

confusing since these findings indicate the non-robustness of the modern differential analysis

tests rather than a problem with large samples. If the model assumptions are not met, they are

likely to perform poorly in small samples too, although, as discussed, they still could be a better

option than the Wilcoxon test for small samples. I recommend changing this part of the title.

*Response: We thank the reviewer for the suggestion. We added “or not” to our title to avoid the*

*confusion: “A large-sample crisis or not? Exaggerated false positives reported by popular*

*differential expression methods.”*

*In fact, although sample size is surely not the reason for model misspecification (when a*

*parametric model is misspecified, the model would have poor performance regardless of the*

*sample size), we could not have observed the inflated FDRs by permuting real datasets if the*

*sample size is small (see our response to the reviewer’s Comment 1). Hence, the phenomenon*

*we observed appeared to be a “large-sample crisis”, but it was not. As Reviewer 1 said, we tried*

*to write a detective-like story, and we would like to use this title to pique biologists’ interest*

*because, as we wrote in our response to the reviewer’s Comment 1, many biologists did not well*

*receive the inflated FDR messages in previous papers.*

10. Page 5, line 56: "Surprisingly, DESeq2 and edgeR identified more DEGs from 84.88% and

78.89% of these permuted datasets than from the original dataset" – This sentence is confusing.

Please rephrase.

*Response: We apologize for the confusing description. We have changed this sentence to*

*"Surprisingly, DESeq2 and edgeR had 84.88% and 78.89% chances, respectively, to identity more*

*DEGs from the permuted datasets than from the original dataset."*

Responses to Reviewer #3

This manuscript addresses an important question on the performance of software for identifying

DE genes using RNA-seq by comparing 6 methods on semi-synthetic datasets. In particular, the

authors found that (1) popular model-based packages, such as DEseq2 and edgeR, severely

underestimate FDRs on the permuted datasets, (2) there is a large deviation between DEGs

identified by different methods, notably DEseq2 and edgeR, and (3) Wilcoxon rank sum test, a

general-purpose non-parametric test, outperforms all DEG software when sample size is large

(>8). These findings will provide useful guidance for practitioners on choosing DEG software

for RNA-seq analyses. Some of the findings resonate with this reviewer's own observation with

the software DEseq2 and edgeR, so it is nice to a systematic analysis on this aspect.

*Response: We thank the reviewer for summarizing our main findings and acknowledging the*

*significance of our study.*

However, comparison of RNA-seq DEG software is not new and some of the findings discussed

in this manuscript have been found before. For example, a google search shows that at least [1-2]

(maybe more) have done some systematic comparisons between DEG software for RNA-seq

analyses. A very well-cited paper [2] (400+ citation) noticed that non-parametric method

(Samseq) outperforms DEseq, edgeR and limma (their Figure 2) when sample size is relatively

large. Similar observations have been discussed in some RNA-seq analysis forum [3].

1. Yet, none of the previous comparisons were mentioned in this manuscript. A comparison with

previous work is needed. In particular, it should be pointed out what is new in the current

comparison and the gain of knowledge over the existing literature on the comparison of DEG

methods. Are the findings different from the existing comparisons? What are the differences and

why?

[1] Corchete, L.A., Rojas, E.A., Alonso-López, D. et al. Systematic comparison and assessment

of RNA-seq procedures for gene expression quantitative analysis. Sci Rep 10, 19737

(2020). https://doi.org/10.1038/s41598-020-76881-x

[2] Fatemeh Seyednasrollah, Asta Laiho, Laura L. Elo, Comparison of software packages for

detecting differential expression in RNA-seq studies, Briefings in Bioinformatics, Volume 16,

Issue 1, January 2015, Pages 59-70, https://doi.org/10.1093/bib/bbt086

[3] SEQanswers: the next generation sequencing

community <http://seqanswers.com/forums/showthread.php?t=20242>

*Response: We are glad that the reviewer is aware of the inflated FDR issue of DESeq2 and*

*edgeR; however, we are afraid that many biologists who analyze RNA-seq data may not know*

*this issue, as evidenced by Reviewer 1’s comment that*

*“The findings should be shocking to the users of the methods investigated, and I hope they are*

*quickly picked up by the community.”*

*We acknowledge that several papers have criticized the inflated FDR issue of DESeq2 and*

*edgeR, but this issue has not been paid enough attention in the field (as we wrote in our*

*manuscript that consortia like GTEx and TCGA still used DESeq2 and edgeR for large-sample*

*DE analysis). A possible reason is that biologists are hardly convinced by model-based simulation results that revealed the inflated FDRs of DESeq2 and edgeR (as in the existing*

*benchmark papers), because they do not know if the models used for simulation mimic the real*

*biology.*

*Hence, our paper is unique in that it solely uses important real datasets (including the*

*immunotherapy dataset as the opening case) to reveal the FDR inflation issue as a concrete crisis,*

*without relying on any additional model assumptions. Please note that our use of semi-synthetic*

*datasets is only for power evaluation and irrelevant to the FDR calibration.*

*The reason why we could use real datasets for FDR calibration is that our datasets have large*

*sample sizes, allowing us to generate many enough permuted datasets for FDR estimation. In*

*contrast, previous studies focused on small sample sizes, a scenario where FDR cannot be*

*reliably estimated by permutation because the number of possible permutation is too small; that*

*is why they all needed to use model-based simulation for FDR estimation, leaving the doubt if*

*their simulated data actually resembled real data.*

*Following the reviewer's suggestion, we have added a supplementary table to summarize the*

*existing benchmark literature (Supplementary Table 2). We have also added the following*

*paragraph to our revised manuscript to highlight the novelty of our study.*

*"Although several studies had evaluated the performance of various DEG identification methods*

*before our study, they had been restricted to using simulated datasets with small sample sizes*

*(Supplementary Table 2). Unlike all previous studies, our study evaluated the FDRs of DEG*

*identification methods by permuting real RNA-seq datasets, without relying on any model*

*assumptions. The reason why we could use real datasets for FDR evaluation is that our datasets*

*have large sample sizes, allowing us to generate many enough permuted datasets for FDR*

*estimation. In contrast, previous studies had focused on small sample sizes, a scenario where*

*FDR cannot be reliably estimated by permutation because the number of possible permutation is*

*too small; that is why they all had to use model-based simulation for FDR estimation, leaving the*

*doubt if their simulated data actually resembled real data.*

*Another innovation of our study is the recommendation of the classical Wilcoxon rank-sum test,*

*which had been ignored by all existing benchmark studies (Supplementary Table 2). For the*

*first time, we found that the Wilcoxon rank-sum test consistently controlled the FDR and*

*achieved good power for DEG identification from large-sample-size RNA-seq data. Although*

*the recently developed dearseq method had claimed it to have overcome the inflated FDR issue*

*of DESeq2 and edgeR on large-sample-size data, our evaluation shows that dearseq still had the*

*issue and did not outperform the Wilcoxon rank-sum test."*

2. Based on this reviewer's own experience, the performance of different software depends on the

proportion of DEG's. This is also discussed in [1]. It will be useful to see how the performance

changes with the proportion of DEG's in this work too. Since this work is based on semisynthetic data, it is easy to do systematic analyses that have different proportions of DEG.

12

[1] Corchete, L.A., Rojas, E.A., Alonso-López, D. et al. Systematic comparison and assessment

of RNA-seq procedures for gene expression quantitative analysis. Sci Rep 10, 19737

(2020). https://doi.org/10.1038/s41598-020-76881-x

*Response: We thank the reviewer for this constructive suggestion. In the revised manuscript, we*

*generated semi-synthetic datasets with four proportions of DEGs (1%, 3%, 5%, and 9%) and*

*evaluated the performance of DEG identification methods accordingly. The results show that the*

*Wilcoxon rank-sum test consistently controlled the FDR and achieved the highest power*

*(conditional on the actual FDRs) across all proportions of DEGs. In contrast, other DEG*

*identification methods consistently failed to control the FDR across all proportions (new*

*Supplementary Fig. 19; pasted below). We have added the following sentences to our revised*

*manuscript to describe these new results.*

*"Considering that the proportion of DEGs might affect the performance of DEG identification*

*methods, we next generated semi-synthetic datasets with four proportions of DEGs (1%, 3%,*

*5%, and 9%) and evaluated the performance of DEG identification methods accordingly. The*

*results show that the Wilcoxon rank-sum test consistently controlled the FDR and achieved the*

*highest power (conditional on the actual FDRs) across all proportions of DEGs (Supplementary*

*Fig. 19). In contrast, other methods consistently failed to control the FDR across all proportions."*

*Supplementary Fig. 19. The Wilcoxon rank-sum test has the best FDR control and power*

*on heart left ventricle vs. atrial appendage GTEx datasets with semi-synthetic ground*

*truths of varying proportions of DEGs.*

*The FDR control (left panel), power (middle panel) given the claimed FDRs, and power given*

*the actual FDRs (right panel) under a range of FDR thresholds from 0.001% to 5% for synthetic*

*datasets with four proportions of DEGs (1%, 3%, 5%, and 9%).*

3. In this work, the author attributed the inflated FDR in DEseq2 and edgeR to lack of fit of the

negative binomial model. While this can be a possible reason, is this the only reason? If so, why

the FDR is better calibrated for these methods when the sample size is larger? Can the authors

look into the genes that are falsely identified in more details?

*Response: We thank the reviewer for this comment and totally agree that there may be other*

*causes for the inflated FDR issue of DESeq2 and edgeR. The inappropriate normalization and*

*dispersion estimation may also attribute to the inflated FDR problem, and they would lead to the*

*poor fit of the negative binomial model we observed from the goodness of fit test results*

*(Supplementary Fig. 3). We also looked into the genes that were falsely identified as DEGs in*

*at least 10 permuted datasets and found one thing these genes have in common: they all have*

*outlier observations under at least one condition (new Supplementary Fig. 5). Specifically, for*

*each gene, a normalized gene expression value is called an outlier if it is greater than Q3+3IQR,*

*where Q3 is the 75% quantile of this gene and IQR is the inter-quantile range. Based on these*

*results, we have added the following paragraph to our manuscript.*

*"Considering that a likely cause of the model violation is the existence of outliers, we examined*

*all the genes that were mistakenly identified as DEGs in at least ten permuted datasets, and we*

*detected the existence of outliers in these genes’ measurements relative to the assumed negative*

*binomial model (Supplementary Fig. 5). It is well known that estimating the mean is not*

*informative in the existence of outliers. However, in parametric methods like edgeR and*

*DESeq2, the null hypothesis of is that a gene has the same mean under the two conditions.*

*Hence, it is expected that the testing result would be severely affected by the existence of*

*outliers. In contrast, the Wilcoxon rank-sum test is more robust to outliers due to its different null*

*hypothesis: a gene’s measurement under one condition has equal chances of being less or greater*

*than its observation under the other condition. That is, the Wilcoxon rank-sum test concerns*

*more about the ranks than the magnitudes of measurements, making it robust to outliers."*

*Supplementary Fig. 5. Histograms showing the distances between the outliers and the third*

*quantile of normalized gene expression values reported by edgeR (upper panel) or DESeq2*

*(lower panel). The results are for the genes that were falsely identified by each method as DEGs*

*in at least 10 permuted datasets.*

*We investigated how sample sizes influence the performance of DEG identification methods*

*(Fig. 2B), and the results show that the FDRs of DESeq2 and edgeR are inflated for almost all*

*sample sizes. The problem with the Wilcoxon rank-sum test is that it does not have power when*

*the sample size is smaller than 8. When the sample size is small, one advantage of DESeq2 and*

*edgeR is that they use the empirical Bayes approach (by borrowing information across genes) to*

*reduce the variance of their estimated gene-specific variance, at the cost of making their*

*estimates biased. However, when the sample size is large, the variance of the estimates becomes*

*small, and the bias dominates, so it is no longer meaningful to use the empirical Bayes approach*

*to reduce variance by introducing bias. Hence, in the large-sample-size scenario, DESeq2 and*

*edgeR have no advantages over the Wilcoxon rank-sum test.*

4. Why were the spurious DEGs enriched in immune-related gene ontology (GO) terms in the

permuted immunotherapy data datasets? Any possibility that the labels were not mixed well

enough?

*Response: To avoid the issue that some permuted datasets might not have the two condition*

*labels mixed well enough, we only preserved the permuted datasets if their fractions of samples*

*with original condition labels were between the 1st and 3rd quartiles of the fractions of all 1000*

*permuted datasets. (In the two extreme cases, the fraction is 1 for the original dataset and 0 for*

*the permuted dataset where the two conditions are swapped, and the samples are not mixed at all;*

*so fractions close to 0 or 1 indicate poor mixing of condition labels.) That is, we ensured that the*

*condition levels were mixed well enough in the permuted datasets we used.*

*To understand why the spurious DEGs were enriched in immune-related GO terms, we evaluated*

*how the negative binomial model (used in edgeR and DESeq2) fit to the two groups of genes on*

*the original dataset: (1) the immune-related spurious DEGs and (2) the genes that were never*

*falsely identified as DEGs from the permuted datasets. The results show that the model fitting*

*was significantly worse for the immune-related spurious DEGs, explaining why these genes were*

*falsely identified as DEGs from the original dataset (new Supplementary Fig. 4).*

*Supplementary Fig. 4. Boxplots showing the poorness of fitting the negative binomial model to the immune-related spurious DEGs identified by DESeq2 or edgeR (“Immunerelated DEGs from permuted datasets” in red) vs. the genes that were never falsely identified as DEGs from the permuted datasets (“Non-DEGs from permuted datasets” in green). Immune-related spurious DEGs are the genes that were falsely identified as DEGs from at least 10% of the permuted datasets and were associated with the immune-related GO terms in Fig. 1E. The poorness of fit of each gene is defined as the negative log10(p-value) from the goodness-of-fit test for the negative binomial distribution based on estimated parameters by DESeq2 or edgeR. The p-value in each panel was calculated by the Wilcoxon rank-sum test to compare the two groups of genes' poorness-of-fit values.*

5. Though Wilcoxon performs well when sample size is >8, it is lack of power when sample size

is below 8. So the claim in the abstract ("Based on these results, for population-level RNA-seq

studies, we recommend the Wilcoxon rank-sum test.") seems to be too broad. What is the

recommendation for sample size <8?

*Response: We apologize for the confusion. What we meant by “population-level RNA-seq*

*studies” are the studies with large sample sizes. To avoid the confusion, we have changed the*

*abstract sentence to*

*"Based on these results, for population-level RNA-seq studies with large sample sizes, we*

*recommend the Wilcoxon rank-sum test."*

*Since this study focus on large samples, our recommendation of the Wilcoxon rank-sum test is*

*for the population-level RNA-seq studies. For small-sample-size studies, we recommend our*

*recent paper that proposed a p-value-free approach for FDR control (Ge, X., Chen, Y.E., Song,*

*D. et al. Clipper: p-value-free FDR control on high-throughput data from two conditions.*

*Genome Biol 22, 288 (2021). https://doi.org/10.1186/s13059-021-02506-9).*

6. Though several sets of datasets were used for demonstration, it is unclear what are the

differences between the characteristics of these datasets. What is the rationale to choose them?

Can they represent a good coverage of different situations that practitioners would encounter in

practice?

*Response: We thank the reviewer for this important question. Since we focused on large*

*samples, we chose RNA-seq datasets from spotlight population-level studies (where samples are*

*from healthy individuals or patients) including GTEx, TCGA, and an immunotherapy study. We*

*did not select datasets by any criteria other than the sample size.*

*In particular, GTEx and TCGA are two consortia that generated large-scale RNA-seq datasets.*

*On GTEx datasets, differential expression analysis can be performed between two tissues or cell*

*types. On TCGA datasets, differential expression analysis can be performed between two disease*

*statuses or biological conditions. The immunotherapy dataset represents an important research*

*area where differential expression is performed to understand the effectiveness of*

*immunotherapy treatment.*

*We have described these datasets’ characteristics and our rationale for choosing them in the*

*revised manuscript. We think that importance of these datasets will help draw biologists’*

*attention to the importance of FDR control in differential expression analysis.*

**Second round of review**

**Reviewer 1**

A large-sample crisis or not? Exaggerated false positives by popular differential

expression methods

I thank the authors for addressing my queries and those of the other reviewers in the previous review round. Some of the clarifications have raised some follow-up concerns, however. I have two major comments that must be addressed.

Major comments.

1. I am happy the issue of 668 permutations was clarified. However, the discarding of "poorly mixed" permutations is not valid methodology and may result in biased estimation of FDR. See e.g. Southworth et al (2009) for a clarification. Please redo ALL calculations using ALL 1000 permutations.

2. In the middle of the same page there is a discussion of the number of genes identified in at least one of the permutations. This number can be made arbitrarily high simply by generating enough permutations, and is therefore not informative. The only informative statements use the average number of rejections over permutations. All statements in this section should be reformulated accordingly, including the statement in the abstract, if that also refers to detections in at least one permutation (this is not clear there). The GO enrichment analysis of the genes found at least once can stay; it is indicative only.

Southworth, L. K., Kim, S. K., & Owen, A. B. (2009). Properties of balanced permutations. Journal of Computational Biology, 16(4), 625-638.

**Reviewer 2**

The authors' responses are mostly satisfactory. I have two minor comments:

Authors' response of comment #2: I meant to say "proportion of false positives out of total number of genes" as opposed to "proportion of false positives out of all positives". So, it won't be 100% whenever a positive is detected from a permuted data. The idea is that it is informative for the reader to see what proportion of null cases are identified as positives, in other words, it gives a sense about the size of the tests. It is true that we are not directly putting bounds on size, and controlling the FDR instead, but I think it is still useful to know what proportion of null genes are falsely discovered by the methods. However, I can see that some may disagree about this.

Authors' response of comment #8: There is no Figure 2C. Did the authors mean FIgure 2A? If not, they should correctly label Figure 2C.

**Reviewer 3**

The authors have addressed several questions satisfactorily. Here are some remaining issues.

1. As pointed out by this reviewer (Round 1 comment 1) and reviewer 2 (Round 1 comment 1), the anticonservative behavior of DEseq2, edgeR, etc. has been reported earlier in the literature by several papers, so the findings in this manuscript are not that surprising. The authors should give a more complete account of the previous report of this anticonservative behavior in the literature at the place when they report their findings. Currently, the authors added a paragraph close to the end of the revised manuscript (page 11) to describe the differences between their analyses and the previous comparison studies. Though pointing out the differences is important, it is also very important to give a fair and complete account of the previous report of the similar findings.

2. To respond my round 1 comment 2, the authors added a simulation to evaluate the impact of different proportions of DEGs (1, 3, 5, 9% DEGs).

(a) Though the simulation is helpful, the range of the DEG proportions is quite narrow. When one compares samples from different developmental stages or different tissues, the proportion of DEGs can be much larger. It will be helpful to see a simulation with a higher proportion of DEGs.

(b) In the title of each subfigure in the supplementary figure 19, it says "Proportion of DEG: 1% (10% of true DEG)". What does the part in the parentheses mean?

3. In the response to my round 1 comment 3:

(a) In the Supplementary Figure 5, what does "Distance (IQR)" mean? Does it use IQR as a unit in these two figures, i.e. 10 means 10 IQR from the 3rd quartile?

(b) In multiple places when IQR is mentioned, it is written as "quantile". It should be "quartile" in IQR and also in 1st or 3rd quartile, not quantile.

Minor:

1. The phrase "many enough permuted datasets" is awkward in the sentence "The reason why we could use real datasets for FDR evaluation is that our datasets have large sample sizes, allowing us to generate many enough permuted datasets for FDR estimation."

Please rephrase.

2. The part "dearseq method had claimed it to have overcome" is awkward in the sentence. "Although the recently developed dearseq method had claimed it to have overcome the inflated FDR issue of DESeq2 and edgeR on large-sample-size data, our evaluation shows that dearseq still had the issue and did not outperform the Wilcoxon rank-sum test. "

Please rephrase.

An English check for the entire article will be beneficial.

**Authors Response**

**Point-by-point responses to the reviewers’ comments:**

*Responses to Reviewer #1:*

I thank the authors for addressing my queries and those of the other reviewers in the previous

review round. Some of the clarifications have raised some follow-up concerns, however. I have

two major comments that must be addressed.

Major comments.

1. I am happy the issue of 668 permutations was clarified. However, the discarding of "poorly

mixed" permutations is not valid methodology and may result in biased estimation of FDR. See

e.g. Southworth et al (2009) for a clarification. Please redo ALL calculations using ALL 1000

permutations.

Southworth, L. K., Kim, S. K., & Owen, A. B. (2009). Properties of balanced permutations.

Journal of Computational Biology, 16(4), 625-638.

*Response: We thank the reviewer for this comment. We totally agree that discarding “poorly*

*mixed” permutations will lead to ill-posed p-values in the permutation test, which is the main*

*conclusion of the Southworth et al. paper. However, we would like to clarify that we did not use*

*the permuted datasets for the permutation test. Instead, we used the well-mixed permuted*

*datasets to find a conservative estimate of the FDR (because DE genes called from permuted*

*datasets are false positives, and fewer DE genes are expected to be called from well-mixed*

*datasets). We found that even with this conservative FDR estimate, popular methods like*

*DESeq2 and edgeR still have severe FDR inflation.*

*Nevertheless, we agree with the reviewer that it is unnecessary and ad-hoc to discard poorlymixed permuted datasets. Therefore, we reran the whole analysis using all 1000 permuted datasets following the reviewer’s suggestion, and the new results are consistent with our old results, so our conclusions remain valid.*

2. In the middle of the same page there is a discussion of the number of genes identified in at

least one of the permutations. This number can be made arbitrarily high simply by generating

enough permutations, and is therefore not informative. The only informative statements use the

average number of rejections over permutations. All statements in this section should be

reformulated accordingly, including the statement in the abstract, if that also refers to detections

in at least one permutation (this is not clear there). The GO enrichment analysis of the genes

found at least once can stay; it is indicative only.

*Response: We thank the reviewer for raising this important point. We have revised our*

*calculation and statements below.*

*"In particular, DESeq2 and edgeR mistakenly identified 30 and 233 genes as DEGs, respectively,*

*from at least 50% of the permuted datasets. Even more, among the DEGs that DEseq2 and edgeR*

*identified from the original dataset, 22 and 194 DEGs were identified from at least 50% of*

*permuted datasets, suggesting that these DEGs were spurious."*

Responses to Reviewer #2:

The authors' responses are mostly satisfactory. I have two minor comments:

Authors' response of comment #2: I meant to say "proportion of false positives out of total

number of genes" as opposed to "proportion of false positives out of all positives". So, it won't be

100% whenever a positive is detected from a permuted data. The idea is that it is informative for

the reader to see what proportion of null cases are identified as positives, in other words, it gives

a sense about the size of the tests. It is true that we are not directly putting bounds on size, and

controlling the FDR instead, but I think it is still useful to know what proportion of null genes

are falsely discovered by the methods. However, I can see that some may disagree about this.

*Response: Following the reviewer’s suggestion, we have added the proportion of false positives*

*out of the total number of genes to a new y-axis on the right of Figure 1A & C and relevant*

*panels in the supplementary figures.*

Authors' response of comment #8: There is no Figure 2C. Did the authors mean FIgure 2A? If not, they should correctly label Figure 2C.

*Response: Yes, it should be Figure 2A and there is no Figure 2C. We apologize for this typo and thank the reviewer for pointing this out.*

Responses to Reviewer #3:

The authors have addressed several questions satisfactorily. Here are some remaining issues.

1. As pointed out by this reviewer (Round 1 comment 1) and reviewer 2 (Round 1 comment

1), the anticonservative behavior of DEseq2, edgeR, etc. has been reported earlier in the

literature by several papers, so the findings in this manuscript are not that surprising. The authors

should give a more complete account of the previous report of this anticonservative behavior in

the literature at the place when they report their findings. Currently, the authors added a

paragraph close to the end of the revised manuscript (page 11) to describe the differences

between their analyses and the previous comparison studies. Though pointing out the differences

is important, it is also very important to give a fair and complete account of the previous report

of the similar findings.

*Response: Following the reviewer’s suggestion, we have added a new paragraph to*

*acknowledge the literature on Page 2 of our revised manuscript.*

*"From the literature, we found that several studies had reported the anti-conservative behavior of*

*DESeq2 and edgeR9-11; however, they were restricted to using simulated datasets with small*

*sample sizes. Hence, for our large-sample-size scenario, their findings did not provide a direct*

*answer to our question. Nevertheless, large sample sizes allowed us to generate permuted*

*datasets to evaluate the FDRs without relying on model assumptions. "*

2. To respond my round 1 comment 2, the authors added a simulation to evaluate the impact

of different proportions of DEGs (1, 3, 5, 9% DEGs).

(a) Though the simulation is helpful, the range of the DEG proportions is quite narrow. When

one compares samples from different developmental stages or different tissues, the proportion of

DEGs can be much larger. It will be helpful to see a simulation with a higher proportion of

DEGs.

*Response: We thank the reviewer for this suggestion. We have included a simulation with DEG*

*proportion of 20% (Supplementary Figure 19; pasted below).*

Supplementary Fig. 19. The Wilcoxon rank-sum test has the best FDR control and power on heart left ventricle vs. atrial appendage GTEx datasets with semi-synthetic ground truths of varying proportions of DEGs. The FDR control (left panel), power (middle panel) given the claimed FDRs, and power given the actual FDRs (right panel) under a range of FDR thresholds from 0.001% to 5% for semisynthetic datasets with five proportions of DEGs (1%, 3%, 5%, 9%, and 20%).

(b) In the title of each subfigure in the supplementary figure 19, it says “Proportion of DEG:

1% (10% of true DEG)”. What does the part in the parentheses mean?

*Response: We apologize for this confusing description. The text in the parentheses was about*

*our definition of true DEGs in our semi-synthetic datasets. To avoid confusion, we have removed*

*it in the revised manuscript.*

3. In the response to my round 1 comment 3:

(a) In the Supplementary Figure 5, what does "Distance (IQR)" mean? Does it use IQR as a

unit in these two figures, i.e. 10 means 10 IQR from the 3rd quartile?

*Response: Yes, we use IQR as a unit in these two figures. To make it clear, we have changed*

*“Distance (IQR)” to “Distance/IQR” and also changed the figure legend to*

*"The x-axis is (the distance between the largest outlier and the 3rd quartile of each gene) / (the*

*inter-quartile range (IQR) of each gene)."*

(b) In multiple places when IQR is mentioned, it is written as “quantile”. It should be

“quartile” in IQR and also in 1st or 3rd quartile, not quantile.

*Response: We thank the reviewer for pointing out this typo. We have corrected it throughout our*

*revised manuscript.*

Minor:

1. The phrase “many enough permuted datasets” is awkward in the sentence “The reason why

we could use real datasets for FDR evaluation is that our datasets have large sample sizes,

allowing us to generate many enough permuted datasets for FDR estimation.”

Please rephrase.

*Response: We have rephrased the sentence to*

*"We could use real datasets for FDR evaluation because our datasets have large sample sizes,*

*allowing us to generate a large number of permuted datasets to ensure accurate FDR estimation."*

2. The part “dearseq method had claimed it to have overcome” is awkward in the sentence.

“Although the recently developed dearseq method had claimed it to have overcome the inflated

FDR issue of DESeq2 and edgeR on large-sample-size data, our evaluation shows that dearseq

still had the issue and did not outperform the Wilcoxon rank-sum test. “

Please rephrase

*Response: We have rephrased the sentence to*

*"Although the recently developed dearseq method was claimed to overcome the inflated FDR*

*issue of DESeq2 and edgeR, our evaluation shows that dearseq still had the issue and did not*

*outperform the Wilcoxon rank-sum test."*

An English check for the entire article will be beneficial.

*Response: We thank the reviewer for this suggestion. We have consulted professional language*

*editing and revised the manuscript accordingly.*

**Round three reviews**

**Reviewer 1:**

My comments were fully addressed.
